# Temperature increase prevails over acidification in gene expression modulation of amastigote differentiation in *Leishmania infantum*

**DOI:** 10.1186/1471-2164-11-31

**Published:** 2010-01-14

**Authors:** Pedro J Alcolea, Ana Alonso, Manuel J Gómez, Alicia Sánchez-Gorostiaga, Mercedes Moreno-Paz, Eduardo González-Pastor, Alfredo Toraño, Víctor Parro, Vicente Larraga

**Affiliations:** 1 Departamento de Microbiología Molecular y Biología de las Infecciones, Centro de Investigaciones Biológicas, Consejo Superior de Investigaciones Científicas (CSIC), calle Ramiro de Maeztu, 9, 28040, Madrid, Spain; 2 Laboratorio de Ecología Molecular and Unidad de Secuenciación y Bioinformática, Centro de Astrobiología, Instituto Nacional de Técnica Aeroespacial "Esteban Terradas" (INTA) and CSIC, carretera de Ajalvir, Km 4, 28850, Torrejón de Ardoz, Spain; 3 Servicio de Inmunología, Centro Nacional de Microbiología, Virología e Inmunología Sanitarias, Instituto de Salud Carlos III (ISCIII), carretera Majadahonda-Pozuelo, Km 2, Majadahonda, Spain

## Abstract

**Background:**

The extracellular promastigote and the intracellular amastigote stages alternate in the digenetic life cycle of the trypanosomatid parasite *Leishmania*. Amastigotes develop inside parasitophorous vacuoles of mammalian phagocytes, where they tolerate extreme environmental conditions. Temperature increase and pH decrease are crucial factors in the multifactorial differentiation process of promastigotes to amastigotes. Although expression profiling approaches for axenic, cell culture- and lesion-derived amastigotes have already been reported, the specific influence of temperature increase and acidification of the environment on developmental regulation of genes has not been previously studied. For the first time, we have used custom *L. infantum *genomic DNA microarrays to compare the isolated and the combined effects of both factors on the transcriptome.

**Results:**

Immunofluorescence analysis of promastigote-specific glycoprotein gp46 and expression modulation analysis of the amastigote-specific A2 gene have revealed that concomitant exposure to temperature increase and acidification leads to amastigote-like forms. The temperature-induced gene expression profile in the absence of pH variation resembles the profile obtained under combined exposure to both factors unlike that obtained for exposure to acidification alone. In fact, the subsequent fold change-based global iterative hierarchical clustering analysis supports these findings.

**Conclusions:**

The specific influence of temperature and pH on the differential regulation of genes described in this study and the evidence provided by clustering analysis is consistent with the predominant role of temperature increase over extracellular pH decrease in the amastigote differentiation process, which provides new insights into *Leishmania *physiology.

## Background

The life cycle of the trypanosomatid parasite *Leishmania *is digenetic because it is developed in two distinct hosts. Promastigote is the extracellular stage and differentiates inside the gut of female phlebotominae sand-fly vectors, which then transmit the parasite to the definitive mammalian host during blood meal intakes [1]. Once inside the dermis, some promastigotes interact with phagocytes and are internalised in parasitophorous vacuoles (phagolysosomes), where they differentiate into the intracellular amastigote stage and multiply [2,3]. Amastigotes are released and infect other phagocytes when the host cell collapses. Remarkable features of the new harsh environment are acidic pH (4.5-5.5) and the physiological temperature of the mammalian host (32-37°C).

Phagolysosomal conditions can be mimicked *in vitro *to grow axenic cultures of the amastigote stage. However, there is not agreement about the equivalence of these forms to amastigotes obtained from their natural environment. In fact, axenic amastigotes are considered as amastigote-like forms (AL) by several authors (e.g. [4,5]), as they show slightly different features from those of amastigotes obtained from host cells. *In vitro *research supported that concomitant exposure to elevated temperatures and acidic pH triggers differentiation of promastigotes to amastigotes [6,7]. Specifically, this could be achieved by combining pH 5.5 and 37°C in the presence of 5-7% CO_2 _[6] or at pH 4.5 and 37°C [8] in a host-free medium. *Leishmania *promastigotes also cope with temperature increase in the absence of pH variation and vice versa [9]. The isolated effects of each factor also induce developmentally regulated changes in the shape and gene expression of promastigotes, but neither of these environmental conditions alone leads to a complete differentiation of promastigotes to amastigotes. Moreover, there is no agreement about the effect of temperature increase. On the one hand, it has been reported that this factor stimulates the entry of promastigotes into stationary phase [10], whereas Shapira *et al*. [9] on the other hand, observed a different effect with both light and scanning electron microscopy: cell shape was round resembling amastigotes but the flagellum still clearly emerged from the cellular body. Regarding the effect of extracellular pH decrease in the absence of temperature variation, it has been stated that generation time increases and a specific protein of the amastigote stage is expressed under these conditions [11] and that acidification itself leads to the differentiation of promastigotes to metacyclic forms in 48 h; these cells then differentiate to amastigotes but only when the temperature is increased [12].

A descriptive differentiation sequence of promastigotes to amastigotes has been proposed: (1) differentiation signal, 0-4 h; (2) disappearance of cell motility, G1 arrest and aggregation, 5-9 h; (3) change of shape, 10-24 h; and (4) completion of subsequent differentiation processes, 25-120 h. The adaptations necessary for survival in the new harsh conditions inside the host cell are mainly due to gene expression modulation. The expression profiles of several genes during this complex differentiation process have been studied. For instance, the A2 gene is up-regulated in the first step, as well as an amastigote-specific proline transporter in the last step. In contrast, 3'-nucleotidase/nuclease (3'NT/Nase) is down-regulated and pentavalent antimonial resistance decreases, presumably due to sodium stibogluconate-resistance protein (SbGRP) expression down-regulation in the same step (reviewed in [6,7,11,13,14]). In addition, partial gene expression profiling of *L. major, L. mexicana, L. infantum *and *L. donovani *amastigotes (axenic and lesion-derived) with respect to promastigotes has been reported [15,19]. However, the effects on the transcriptome of particular factors that influence differentiation *in vivo *(mainly temperature increase and pH decrease) have not been studied to date. So in this study we have analysed, for the first time, the concomitant (TPS) and the isolated effects of temperature and pH shift (respectively, TS and PS) relative to control promastigote culture conditions (CC) on the transcriptome of *L. infantum *by custom genomic DNA microarrays.

TPS-treated promastigotes differentiate to AL with regard to the up-regulation of the amastigote-specific A2 gene and the absence of promastigote-specific glycoprotein 46 (gp46) expression as verified by indirect immunofluorescence assay (IFA). In addition, the up-regulation of several amastin genes and the down-regulation of 3'NT/Nase and SbGRP genes under TPS and TS is in agreement with previous data (reviewed in [13]). None of these genes have been found to be differentially regulated under PS. As a consequence, TPS-treated cells are AL and TS-treated ones are also progressing towards amastigote differentiation but PS-treated cells do not seem to undergo the same differentiation process. After performing IFA, transcriptome analysis was carried out and a large set of genes differentially regulated by the effect of both factors was found. A broader analysis of their influence on differentiation at the gene expression modulation level by multi-experimental Serial Analysis of Microarrays (SAM) and iterative hierarchical clustering analysis (HCL-ST) of genes with respect to their expression modulation has led us to conclude that temperature increase has a greater influence than pH decrease on the differentiation process of promastigotes to amastigotes.

## Results and Discussion

### Cell growth, gp46-IFA and microarray hybridisation analysis and validation

**Figure 1:**
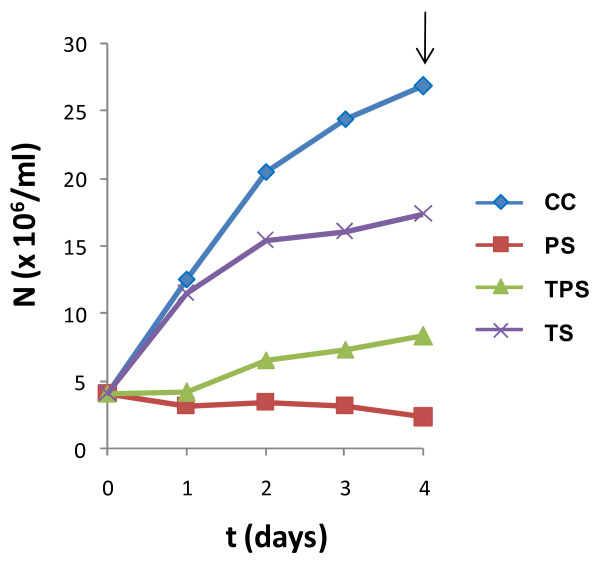
**Average growth curves of control and temperature/pH-treated *L. infantum *promastigotes**. Three replicates of the cultures were performed for each of the conditions assayed. RNA samples were extracted and processed for transcriptome analysis on day 4. Growth arrest is induced by pH decrease.

Growth curves of promastigotes cultured under CC (from the mid-logarithmic to the early stationary phase), TPS, TS and PS conditions are represented in Figure 1. Proliferation decrease is more noticeable under PS conditions than under TPS and TS. Therefore, pH decrease inhibits proliferation of parasites at both 37°C and 27°C, which is in agreement with previous findings [11]. Consequently, TPS-treated promastigotes show more pronounced proliferation detention than TS due to the effect of acidification (Figure 1). Taken together, these data are consistent with cell proliferation arrest during the differentiation process leading up to the amastigote stage, after which mature amastigotes are able to multiply.

**Figure 2:**
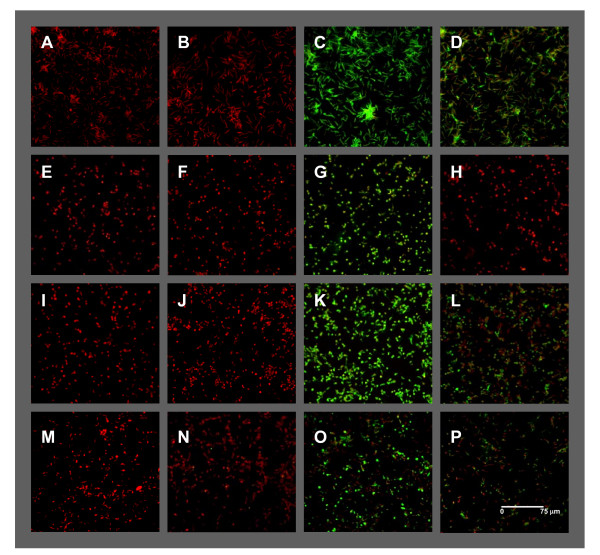
**gp46 IFA**. Samples of all the experimental conditions described in this article were collected on day 4 for IFA analysis. (A-D) CC; (E-H) TPS; (I-L) TS; and (M-P) PS. Incubations were performed with: PBS as negative control for the FITC-conjugated anti-mouse IgG secondary antibody (A, E, I, M); monoclonal anti-rabbit complement factor H primary antibody negative control (B, F, J, N); SIM110 monoclonal anti-SLA as positive control (C, G, K, O); and monoclonal anti-gp46 (D, H, L, P). As a summary, gp46 is expressed under CC, TS and PS but not in TPS-treated AL.

Surface glycoprotein gp46 is known to be promastigote-specific. In fact, it is also called promastigote surface antigen 2 (PSA2) [20]. This glycoprotein has not been detected in amastigotes, although transcripts have been detected at this stage [21]. We have used a monoclonal antibody against gp46 in IFA to assess its expression under CC, TPS, TS and PS conditions, and the absence of gp46 expression can only be observed in the case of TPS (Figure 2). These findings provide evidence for an AL stage after 4 days of TPS exposure. Consequently, TPS-treated cells undergo a more intensive differentiation process leading up to AL than TS and PS-treated cells.

**Figure 3:**
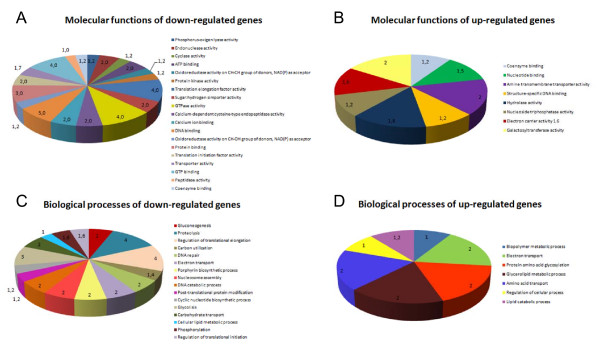
**Multilevel sector charts of α-scores for GO molecular functions annotated on differentially regulated genes under TPS**. (A) Molecular function GO terms annotated on down-regulated genes under TPS. (B) Molecular function GO terms annotated on up-regulated genes under TPS. (C) Biological process GO terms annotated on down-regulated genes under TPS. (D). Biological process GO terms annotated on up-regulated genes under TPS.

**Figure 4:**
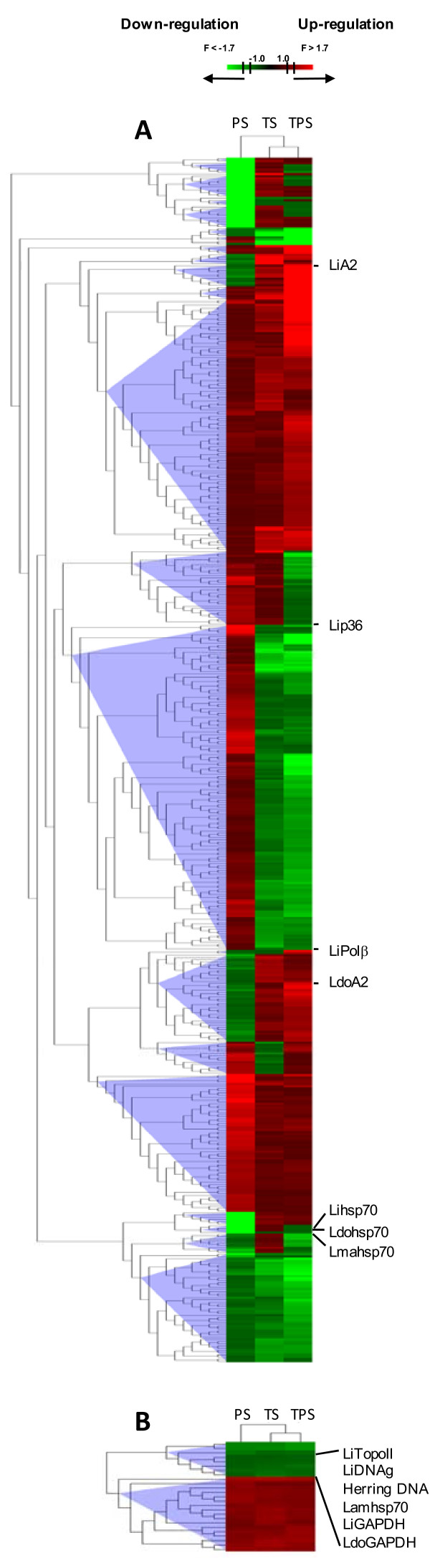
**HCL-ST of genes differentially modulated under TPS, TS and/or PS**. After performing SAM for all experimental groups, HCL-ST analysis was performed independently for (A) genes with (B) and without significant differences between groups according to SAM. Support Tree algorithm with a jackknifing resampling option and 100 iterations for the construction and clustering of gene expression matrix were applied in HCL-ST. Clones in (A) were grouped into 26 clusters and clones in (B) in two clusters depending on differential regulation. This analysis confirms that expression profile similarity is higher between TPS and TS than between TPS or TS and PS. Control spots LiA2, LdoA2, Lip36, Lipolb, Lihsp70, Ldohsp70 and Lmahsp70 show significant differences in gene expression between the experimental groups (A2 gene is up-regulated under TPS and hsp70 under PS) and LiTopoII, LiDNAg, Lamhsp70, LiGAPDH, LdoGAPDH and herring DNA do not. Clones with significant differences between the experimental conditions are identified in Additional file 6.

Total RNA was extracted and its integrity and absence of DNA contamination were checked by capillary electrophoresis in samples obtained on day 4 (Additional file 1). After mRNA amplification, cDNA was synthesised and labelled with Cy3 for CC and with Cy5 for each of the conditions assayed. DNA microarray hybridisations with these cDNA samples (TPS vs. CC, TS vs. CC and PS vs. CC) were carried out in triplicate. Subsequently, local background was substracted and raw data were normalized and t-test performed for three replicates. A total of 225 spots for TPS, 102 for TS and 117 for PS vs. CC were selected as they fulfilled the following selection criteria: (i) F ≥ 1.7 (Cy5/Cy3 ratio if Cy5 > Cy3) or ≤ -1.7 (-Cy3/Cy5 ratio if Cy3 > Cy5), (ii) total relative fluorescence intensity value > 5000 FU and (iii) *p *< 0.05 (Additional file 2). Clones corresponding to selected spots had their insert ends sequenced and were mapped against the *L. infantum *genome to identify overlapping genes. Normalized and contrasted microarray hybridisation results of those clones that contain known annotated genes are described in Tables 1 and 2 for TPS, 3 and 4 for TS and 5 for PS. Hypothetical and unknown genes found to be regulated differentially are described in (Additional file 3: Table S1, S2, S3, S4, S5 and S6), as well as clones that map against minicircle sequences. Gene expression data obtained by microarray hybridisation assays were validated by relative quantitative real time PCR (qRT-PCR) in 15% clones (12% genes excluding redundancy expected in a shotgun microarray strategy). Molecular function GO annotations are indicated in Tables 1, 2, 3, 4 and 5 in order to relate differentially regulated genes with direct acyclic graphs (DAGs) (Additional file 4) and molecular function multilevel sector charts (Figure 3). Once the individual effect of each factor on the transcriptome was analysed, a multi-experiment comparison (SAM) was performed to determine if there were statistically significant differences between PS, TS and TPS expression profiles for each of the differentially regulated genes found. Finally, an HCL-ST analysis including control spots allowed us to determine the relative distance between the experimental groups: TS is closer than PS to the TPS profile (Figure 4).

**Table 1:** Up-regulated genes after 37°C/pH4.5 treatment (day 4) in *L. infantum*.

*Clone*	*F*	*Log*_2_*F ± SD*	*p*	*GenBank*	*e-value*		*Def*.	*Id*.	*Annotated Gene Function (GO terms in Figure S3., additional *file 4)	*qRT-PCR*	
				*GSS*	*Fw*	*Rv*				+/-	F ± SD
Lin11D7	4.78	2.3 ± 0.2	0.002	GS598854	6e-118	0	b	**LinJ31_V3.0460**	**Amastin, putative (uTPS0)**	+	7.8 ± 0.2
Lin19D1	1.88	0.9 ± 0.3	0.028	GS598855	3e-18	0	b	**LinJ08_V3.0680**	**Amastin-like protein (uTPS0)**	N.D.	
								**LinJ08_V3.0690**	**Amastin-like protein (uTPS0)**	N.D.	
Lin22E12	2.79	1.5 ± 0.1	0.001	GS598856	0	0	b	LinJ31_V3.1850	Amino acid permease (uTPS13)	N.D.	
Lin33G2	4.01	2.0 ± 0.8	0.044	GS598857	6e-118	6e-118	b	**LinJ34_V3.4370**	**Amastin-like surface protein, putative (uTPS0)**	N.D.	
Lin34G1	1.81	0.9 ± 0.1	0.008	GS598858	0	0	a	LinJ16_V3.0790	Chitinase (uTPS18, uTPS8)	N.D.	
Lin36B8	1.99	1.0 ± 0.2	0.015	GS598859	0	0	b	LinJ30_V3.3230	3-hydroxy-3-methyglutaryl-CoA reductase, putative (uTPS21, uTPS12)	N.D.	
Lin50G2	3.38	1.8 ± 0.1	0.000	GS598860	0	4e-153	b	**LinJ34_V3.2660**	**Amastin-like surface protein (uTPS0)**	+	1.8 ± 0.0
Lin54G3	1.84	0.9 ± 0.3	0.027	GS598861	0	0	b	LinJ24_V3.1230	Hypothetical protein, conserved	N.D.	
								**LinJ24_V3.1240**	**Translation factor SUI1, putative (uTPS0)**	+	3.1 ± 0.1
Lin62D3	1.92	0.9 ± 0.4	0.040	GS598862	0	0	b	LinJ05_V3.0340	Hypothetical protein, conserved	N.D.	
								LinJ05_V3.0350	Trypanothione reductase (uTPS10, uTPS14, uTPS20)	+	3.8 ± 0.4
Lin62D10	1.76	0.8 ± 0.3	0.051	GS598863	0	0	b	LinJ17_V3.1150	Esterase-like protein (uTPS5)	+	18.5 ± 1.5
Lin66A8	3.59	1.8 ± 0.4	0.013	GS598864	0	0	b	LinJ22_V3.0470	Hypothetical protein, conserved	N.D.	
								**LinJ22_V3.0480**	**Ubiquitin-conjugating enzyme-like protein (uTPS0)**	+	2.3 ± 0.2
Lin66F8	1.92	0.9 ± 0.2	0.017	GS598865	0	3e-132	a	LinJ33_V3.2470	Succinyl-CoA:3-ketoacid-CoA transferase, mitochondrial precursor, putative (3-oxoacid-CoA transferase)	-	-1.3 ± 0.3
								LinJ33_V3.2480	Hypothetical protein, conserved/RABreg (uTPS17)	N.D.	
Lin77H8	5.63	2.5 ± 0.5	0.016	GS598866	0	0	b	**LinJ08_V3.0690**	**Amastin-like protein (uTPS0)**	N.D.	
Lin86H7	3.06	1.6 ± 0.2	0.004	GS598867	0	2e-101	b	**LinJ08_V3.0700**	**Amastin-like protein (uTPS0)**	+	9.5 ± 0.3
Lin87H2	4.20	2.1 ± 0.3	0.008	GS598868	3e-15	3e-33	b	**LinJ08_V3.0690**	**Amastin-like protein (uTPS0)**	N.D.	
Lin89D9	1.71	0.8 ± 0.3	0.043	GS598869	0	0	b	**LinJ21_V3.0770**	**ATP-binding cassette sub-family E, putative (uTPS9, uTPS11, uTPS24, uTPS28)**	N.D.	
Lin90B6	1.71	0.8 ± 0.3	0.040	GS598870	0	0	b	**LinJ30_V3.0640**	**Ribosome biogenesis regulatory protein (RRS1), putative (uTPS0)**	+	16.4 ± 0.2
								LinJ30_V3.0650	Histidyl-tRNA synthetase, putative	N.D.	
								LinJ30_V3.0660	Hypothetical protein, conserved	N.D.	
Lin90H2	1.76	0.8 ± 0.1	0.005	GS598871	0	0	b	LinJ30_V3.2200	RNA-binding protein (uTPS3, uTPS6, uTPS15, uTPS16)	N.D.	
Lin91B12	5.24	2.4 ± 0.3	0.004	GS598872	0	0	b	**LinJ34_V3.2660**	**Amastin-like surface protein (uTPS0)**	+	1.8 ± 0.0
Lin92H5	2.48	1.3 ± 0.5	0.041	GS598873	0	0	b	LinJ28_V3.2060	Zinc transporter, putative (uTPS23)	+	45.7 ± 0.5
								LinJ28_V3.2070	Hypothetical protein, conserved	N.D.	
Lin93E3	1.83	0.9 ± 0.4	0.033		0	0	b	LinJ10_V3.0410	Pteridine transporter ft3, putative (uTPS0)	N.D.	
Lin104C10	6.68	2.7 ± 0.1	0.001	GS598874	0	0	b	**LinJ08_V3.1320**	**Amastin-like protein (uTPS0)**	N.D.	
Lin106A1	2.43	1.3 ± 0.0	0.000	GS598875	0	0	c	LinJ06_V3.1200	Hypothetical protein, conserved	N.D.	
								**LinJ31_V3.0590**	**Amino acid transporter aATP11, putative (uTPS13)**	+	2.6 ± 0.0
Lin113C3	1.87	0.9 ± 0.1	0.040	GS598876	3e-74	0	a	**LinJ14_V3.1440**	**Pteridine transporter (uTPS0)**	+	1.9 ± 0.0
								**LinJ14_V3.1450**	**Myo-inositol-1-phosphate synthase (uTPS0)**	+	2.1 ± 0.3
Lin118A11	2.90	1.5 ± 0.3	0.010	GS598877	0	7e-28	c	LinJ30_V3.0630	Nitrate reductase, putative (uTPS0)	+	3.7 ± 0.3
								LinJ36_V3.2480	Glyceraldehyde-3-phosphate dehydrogenase, putative	N.D.	
Lin119F3	3.40	1.8 ± 0.2	0.005	GS598878	0	0	b	LinJ25_V3.2570	Phosphoglycan beta-1,3-galactosyltransferase 6 (uTPS22)	N.D.	
Lin123D6	2.91	1.5 ± 0.1	0.002	GS598879	-	0	c	**LinJ34_V3.2660**	**Amastin-like surface protein (uTPS0)**	+	1.8 ± 0.0
Lin137A10	1.98	1.0 ± 0.3	0.039	GS598880	0	0	b	LinJ24_V3.1230	Hypothetical protein, conserved	N.D.	
								**LinJ24_V3.1240**	**Translation factor SUI1, putative (uTPS0)**	+	3.1 ± 0.1
Lin142H9	1.74	0.8 ± 0.1	0.004	GS598881	0	0	b	**LinJ31_V3.0460**	**Amastin, putative (uTPS0)**	+	7.8 ± 0.2
Lin146E3	2.35	1.2 ± 0.4	0.038	GS598882	0	0	b	**LinJ31_V3.0590**	**Amino acid transporter aATP11, putative (uTPS13)**	+	2.6 ± 0.0
Lin156B2	1.82	0.9 ± 0.2	0.025	GS598883	0	0	b	LinJ33_V3.2960	Hypothetical protein, conserved/Transcription regulator (uTPS 1, uTPS4, uTPS7)	N.D.	
Lin162A9	4.29	2.1 ± 0.2	0.004	GS598884	0	0	b	LinJ22_V3.0470	Hypothetical protein, conserved	N.D.	
								**LinJ22_V3.0480**	**Ubiquitin-conjugating enzyme-like protein (uTPS0)**	+	2.3 ± 0.2
Lin165E2	3.48	1.8 ± 0.2	0.004	GS598885	0	0	b	**LinJ22_V3.0680**	**3'a2rel-related protein (uTPS0)**	+	4.0 ± 0.4
Lin183A3	1.75	0.8 ± 0.1	0.010	GS598886	0	0	b	LinJ24_V3.2250	Hypothetical protein, conserved/GPDE (uTPS26)	N.D.	
Lin185A12	4.53	2.2 ± 0.2	0.002	GS598887	0	0	b	**LinJ34_V3.2660**	**Amastin-like surface protein, putative (uTPS0)**	+	1.8 ± 0.0
Lin188H2	3.20	1.7 ± 0.6	0.042	GS598888	0	0	c	**LinJ08_V3.0680**	**Amastin-like protein (uTPS0)**	N.D.	
Lin194E2	3.22	1.7 ± 0.4	0.023	GS598889	7e-56	4e-153	b	**LinJ08_V3.0710**	**Amastin-like protein (uTPS0)**	+	9.5 ± 0.3
Lin197A12	1.95	1.0 ± 0.2	0.016	GS598890	0	0	a	LinJ31_V3.2540	Lipase, putative (uTPS19)	N.D.	
Lin201F8	2.00	1.0 ± 0.4	0.041	GS598891	0	0	b	LinJ31_V3.3330	Phosphoglycan beta-1,3-galactosyltransferase 5 (uTPS22)	N.D.	
Lin206B6	5.40	2.4 ± 0.5	0.012	GS598892	7e-133	0	b	**LinJ22_V3.0680**	**3'a2rel-related protein **(uTPS0)	+	4.0 ± 0.4
Lin210C4	2.77	1.5 ± 0.1	0.003	GS598893	0	0	b	**LinJ08_V3.0690**	**Amastin-like protein (uTPS0)**	N.D.	
Lin223F2	1.73	0.8 ± 0.3	0.044	GS598894	0	0	b	LinJ13_V3.0330	Unknown/Tubulin associated GTPase (uTPS2, uTPS25, uTPS27)	N.D.	
Lin224G2	2.20	1.1 ± 0.2	0.023	GS598895	0	0	b	**LinJ08_V3.0720**	**Amastin-like protein (uTPS0)0**	N.D.	
Lin235G8	3.16	1.7 ± 0.2	0.003	GS598896	0	0	b	**LinJ08_V3.1320**	**Amastin-like protein (uTPS0)**	N.D.	
Lin245E2	2.61	1.4 ± 0.4	0.040	GS598897	0	0	b	**LinJ22_V3.0680**	**3'a2rel-related protein (uTPS0)**	+	4.0 ± 0.4
Lin267D9	2.06	1.0 ± 0.2	0.010	GS598898	9e-111	0	b	LinJ16_V3.0590	Carbamoyl-phosphate synthetase, putative (uTPS0)	+	2.9 ± 0.4
								LinJ16_V3.0600	Histone H3, putative	-	1.3 ± 0.2
Lin274G6	5.77	2.5 ± 0.2	0.003	GS598899	0	0	b	**LinJ08_V3.0680**	**Amastin-like protein (uTPS0)**	N.D.	
								**LinJ08_V3.0690**	**Amastin-like protein (uTPS0)**	N.D.	
Lin275A8	2.72	1.4 ± 0.2	0.006	GS598900	0	4e-168	b	**LinJ08_V3.0720**	**Amastin-like protein (uTPS0)**	N.D.	
Lin276B6	1.76	0.8 ± 0.3	0.041	GS598901	0	0	b	LinJ31_V3.2540	Lipase, putative (uTPS19)	N.D.	
Lin294A11	4.86	2.3 ± 0.1	0.001	GS598902	0	0	b	**LinJ08_V3.1320**	**Amastin-like protein (uTPS0)**	N.D.	
Lin295D9	4.40	2.1 ± 0.1	0.000	GS598903	0	0	b	**LinJ34_V3.1720**	**Amastin-like surface protein, putative (uTPS0)**	N.D.	
Lin310F2	2.34	1.2 ± 0.5	0.046	GS598904	0	0	b	LinJ23_V3.1220	Hydrophilic surface protein (HASPB) (uTPS0)	N.D.	
cLinA2	6.45	2.7 ± 0.1	0.000	S69693	-	-	-	-	*L. infantum *A2 gene -- DNA microarray control spot		
cLdoA2	2.51	1.3 ± 0.3	0.021	-	-	-	-	-	*L. donovani *A2 gene -- DNA microarray control spot		

This table contains clones that map against up-regulated genes (not hypothetical or unknown) with the combined effect of temperature increase and pH decrease (TPS). The features described are: clone number; F; base-two logarithmic scale F and SD values; *p*; GenBank GSS accession numbers; e-values; Def. according to mapping outcomes *a*, *b *or *c *(see brief explanation in the text); Id.; annotated gene function (codes for Additional file 4: Figure S3,); qRT-PCR. When a given clone overlaps with more than one annotation, stage-specific regulation is only demonstrated if the qRT-PCR result is positive (+). Genes in bold are also up-regulated under TS.

**Table 2:** Down-regulated genes after 37°C/pH4.5 treatment (day 4) in *L. infantum*.

*Clone*	*F*	*Log*_2_*F ± SD*	*P*	*GenBank GSS*	*e-value*		*Def*.	*Id*.	*Annotated Gene Function (GO terms in Figure S4., additional file 4)*	*qRT-PCR*	
					*Fw*	*Rv*				+/-	F ± SD
Lin1G8	-1.80	-0.8 ± 0.1	0.009	GS598905	5e-35	8e-31	B	LinJ22_V3.1340	Serine/threonine protein phosphatase, putative (dTPS6)	N.D.	
Lin4F4	-2.27	-1.2 ± 0.3	0.016	GS598906	0	0	a	LinJ31_V3.0430	Cysteine peptidase, Clan CA, family C2, putative (dTPS0)	+	-3.3 ± 0.1
Lin9B9	-1.71	-0.8 ± 0.2	0.015	GS598907	5e-26	0	a	LinJ36_V3.1010	Dynein heavy chain, putative (dTPS0)	N.D.	
Lin15D6	-2.05	-1.0 ± 0.2	0.042	GS598908	0	0	a	LinJ31_V3.0610	Amino acid transporter aATP11, putative (dTPS0)	N.D.	
Lin22B1*	-2.03	-1.0 ± 0.2	0.016	GS598909	0	0	a	LinJ23_V3.1400	Coronin, putative (dTPS0)	+	-4.9 ± 0.7
Lin21H10	-1.90	-0.9 ± 0.1	0.001	GS598910	0	0	b	LinJ26_V3.1670	Sphingolipid delta-4 desaturase, putative (dTPS22)	N.D.	
Lin24E10*	-1.80	-0.8 ± 0.3	0.038	GS598911	0	0	b	LinJ22_V3.1310	I/6 autoantigen-like protein (dTPS0)	+	-6.7 ± 0.9
Lin27B2	-1.80	-0.8 ± 0.3	0.034	GS598912	0	-	c	LinJ35_V3.1230	Short chain dehydrogenase, putative (dTPS1, dTPS7)	+	-3.2 ± 0.7
Lin28C5	-1.81	-0.9 ± 0.1	0.005	GS598913	0	2e-154	b	LinJ26_V3.1670	Sphingolipid delta-4 desaturase, putative (dTPS22)	N.D.	
Lin31H9*	-1.94	-1.0 ± 0.1	0.006	GS598914	0	0	b	LinJ26_V3.1000	Dynein heavy chain, putative (dTPS0)	+	-6.2 ± 0.8
Lin36A9*	-2.15	-1.1 ± 0.2	0.009	GS598915	0	0	b	LinJ26_V3.1000	Dynein heavy chain, putative (dTPS0)	+	-6.2 ± 0.8
Lin40G12*	-1.92	-0.9 ± 0.2	0.013	GS598916	2e-161	0	b	**LinJ23_V3.1560**	**Lathosterol oxidase-like protein (dTPS28, dTPS30)**	+	-14.3 ± 1.7
Lin47D8	-4.00	-2.0 ± 0.5	0.023	GS598917	0	0	a	**LinJ06_V3.1330**	**Coproporphyrinogen III oxidase, putative (dTPS33)**	+	-5.8 ± 0.1
								LinJ06_V3.1340	Protoporphyrinogen oxidase-like protein (dTPS13, dTPS32)	+	-2.2 ± 0.4
Lin49B7	-4.38	-2.1 ± 0.0	0.000	GS598918	0	4e-64	a	**LinJ34_V3.4160**	**Phosphatidylinositol-3-kinase (tor2)-like protein (dTPS0)**	N.D.	
Lin50H7	-2.32	-1.2 ± 0.1	0.005	GS598919	7e-164	0	b	LinJ28_V3.2380	2,3-bisphosphoglycerate-independent phosphoglycerate mutase-like protein (dTPS9, dTPS35)	+	-3.4 ± 0.3
								LinJ28_V3.2390	Cyclin dependent kinase-binding protein, putative (dTPS0)	+	-127.4 ± 7.4
Lin60B1*	-3.84	-1.9 ± 0.1	0.001	GS598920	0	0	c	**LinJ31_V3.2370**	**3'-nucleotidase/nuclease, putative (dTPS4, dTPS29)**	+	-4.6 ± 0.4
Lin60E5	-1.80	-0.8 ± 0.3	0.035	GS598921	0	0	b	LinJ26_V3.0970	Hypothetical protein, conserved/HPB (dTPS5, dTPS16)	N.D.	
Lin63F3	-2.95	-1.6 ± 0.4	0.018	GS598922	0	0	b	**LinJ36_V3.6550**	**Glucose transporter lmgt2, putative (dTPS47)**	+	-8.1 ± 1.1
								**LinJ36_V3.6560**	**Glucose transporter, putative (dTPS47)**	+	-6.3 ± 1.4
Lin66F10	-2.18	-1.1 ± 0.0	0.000	GS598923	0	0	b	**LinJ36_V3.6550**	**Glucose transporter lmgt2, putative (dTPS47)**	+	-8.1 ± 1.1
								**LinJ36_V3.6560**	**Glucose transporter, putative (dTPS47)**	+	-6.3 ± 1.4
Lin80B3	-2.06	-1.0 ± 0.3	0.024	GS598924	0	0	b	LinJ28_V3.3250	Glucose-6-phosphate-N-acetyltransferase, putative (dTPS46)	N.D.	
Lin82C6	-1.74	-0.8 ± 0.3	0.038	GS598925	0	-	c	LinJ31_V3.0440	Cysteine peptidase, Clan CA, family C2, putative (dTPS0)	+	-3.3 ± 0.1
Lin84E8	-2.26	-1.2 ± 0.2	0.007	GS598926	0	0	a	**LinJ31_V3.2370**	**3'-nucleotidase/nuclease, putative (dTPS4, dTPS29)**	+	-4.6 ± 0.4
								**LinJ31_V3.2380**	**3'-nucleotidase/nuclease precursor, putative (dTPS4, dTPS29)**	+	-2.7 ± 0.6
Lin86H3	-2.17	-1.1 ± 0.1	0.004	GS598927	0	8e-130	b	LinJ31_V3.0950	Sodium stibogluconate-resistance protein, putative (dTPS)	N.D.	
Lin89F9	-2.93	-1.6 ± 0.4	0.026	GS598928	0	0	b	**LinJ31_V3.2370**	**3'-nucleotidase/nuclease, putative (dTPS4, dTPS29)**	+	-4.6 ± 0.4
								**LinJ31_V3.2380**	**3'nucleotidase/nuclease precursor, putative (dTPS4, dTPS29)**	+	-2.7 ± 0.6
								LinJ31_V3.2390	Helicase-like protein	N.D.	
Lin92D7*	-2.27	-1.2 ± 0.2	0.005	GS598929	0	0	b	**LinJ31_V3.2370**	**3'-nucleotidase/nuclease, putative (dTPS4, dTPS29)**	+	-4.6 ± 0.4
								**LinJ31_V3.2380**	**3'-nucleotidase/nuclease precursor, putative (dTPS4, dTPS29)**	+	-2.7 ± 0.6
Lin92G9	-2.29	-1.2 ± 0.1	0.004	GS598930	0	0	a	**LinJ06_V3.1320**	**Pteridine transporter, putative (dTPS0)**	+	-2.1 ± 0.3
								**LinJ06_V3.1330**	**Coproporphyrinogen III oxidase, putative (dTPS33)**	+	-5.8 ± 0.1
Lin97D1	-11.57	-3.5 ± 0.2	0.001	GS598931	0	0	a	**LinJ06_V3.1320**	**Pteridine transporter, putative (dTPS0)**	+	-2.1 ± 0.3
Lin97F6	-1.80	-0.8 ± 0.3	0.029	GS598932	0	0	b	LinJ26_V3.0460	Hypothetical protein, conserved	N.D.	
Lin98C7*	-2.12	-1.1 ± 0.5	0.044	GS598933	0	0	b	**LinJ31_V3.2370**	**3'-nucleotidase/nuclease, putative (dTPS4, dTPS29)**	+	-4.6 ± 0.4
								**LinJ31_V3.2380**	**3'nucleotidase/nuclease precursor, putative (dTPS4, dTPS29)**	+	-2.7 ± 0.6
Lin98F9	-2.43	-1.3 ± 0.3	0.016	GS598934	2e-190	1e-101	b	LinJ32_V3.3120	Minichromosome maintenance (MMC) complex subunit, putative (dTPS8, dTPS38, dTPS48)	N.D.	
Lin98G10*	-2.13	-1.1 ± 0.2	0.015	GS598935	0	0	b	**LinJ30_V3.2780**	**Superoxide dismutase, putative (dTPS0)**	+	-3.7 ± 0.1
Lin101B5	-2.62	-1.4 ± 0.3	0.011	GS598936	0	0	b	**LinJ09_V3.0650**	**Serine peptidase family S51, peptidase E, putative (dTPS19)**	N.D.	
Lin105A3	-2.22	-1.1 ± 0.1	0.005	GS598937	0	0	b	LinJ36_V3.1320	Fructose-1,6-bisphosphate aldolase (dTPS37)	N.D.	
Lin105B9	-6.13	-2.6 ± 0.2	0.001	GS598938	0	0	a	**LinJ06_V3.1320**	**Pteridine transporter, putative (dTPS0)**	+	-2.1 ± 0.3
Lin109F4	-3.16	-1.7 ± 0.5	0.024	GS598939	0	0	a	**LinJ06_V3.1330**	**Coproporphyrinogen III oxidase, putative (dTPS33)**	+	-5.8 ± 0.1
								LinJ06_V3.1340	Protoporphyrinogen oxidase-like protein (dTPS13, dTPS32)	+	-2.2 ± 0.4
Lin111C2	-3.14	-1.7 ± 0.1	0.001	GS598940	0	0	b	**LinJ09_V3.0650**	**Serine peptidase family S51, peptidase E, putative (dTPS19)**	N.D.	
Lin111F3	-2.09	-1.1 ± 0.4	0.035	GS598941	0	0	a	LinJ31_V3.2210	Prostaglandin F2α synthetase (dTPS31)	N.D.	
Lin125H5	-1.95	-1.0 ± 0.2	0.022	GS598942	0	0	b	LinJ36_V3.1590	Serine/threonine protein kinase, putative (dTPS43)	N.D.	
Lin144F11	-2.28	-1.2 ± 0.1	0.004	GS598943	0	0	a	LinJ31_V3.2210	Prostaglandin F2α synthetase (dTPS31)	N.D.	
Lin144H10	-1.85	-0.9 ± 0.1	0.007	GS598944	0	0	a	LinJ22_V3.1300	Cyclophilin, putative (dTPS0)	+	-4.1 ± 0.6
								LinJ19_V3.1310	I/6-autoantigen-like protein (dTPS0)	N.D.	
Lin144H11	-2.32	-1.2 ± 0.1	0.003	GS598945	0	0	b	LinJ36_V3.2700	Membrane-bound acid phosphatase precursor (dTPS42)	N.D.	
Lin150E4	-1.86	-0.9 ± 0.4	0.048	GS598946	0	0	b	**LinJ13_V3.1060**	**Calmodulin, putative (dTPS16)**	N.D.	
Lin153D1	-1.80	-0.8 ± 0.1	0.002	GS598947	0	0	b	LinJ27_V3.0530	Amino acid permease, putative (dTPS26)	N.D.	
Lin155H12	-2.52	-1.3 ± 0.4	0.024	GS598948	0	0	a	LinJ36_V3.0250	**Peptidyl-prolyl cis-trans isomerase, putative (dTPS18, dTPS25)**	N.D.	
Lin157D8	-2.78	-1.5 ± 0.2	0.005	GS598949	0	0	b	**LinJ31_V3.2370**	**3'-nucleotidase/nuclease, putative (dTPS4, dTPS29)**	+	-4.6 ± 0.4
Lin158A10	-2.40	-1.3 ± 0.2	0.008	GS598950	0	0	b	LinJ23_V3.0870	Hypothetical protein, conserved	N.D.	
Lin177E10	-1.72	-0.8 ± 0.3	0.042	GS598951	7e-167	0	b	LinJ16_V3.0600	Histone H3, putative (dTPS8)	N.D.	
								LinJ16_V3.0610	Histone H3, putative (dTPS8)	N.D.	
Lin182F2	-2.18	-1.1 ± 0.3	0.022	GS598952	0	0	b	**LinJ25_V3.0740**	**Eukaryotic initiation factor 5a, putative (dTPS15)**	N.D.	
								**LinJ25_V3.0750**	**Eukaryotic initiation factor 5a, putative (dTPS15)**	N.D.	
Lin187C10	-4.02	-2.0 ± 0.3	0.003	GS598953	0	0	b	**LinJ06_V3.1320**	**Pteridine transporter, putative (dTPS0)**	+	-2.1 ± 0.3
Lin193E6	-2.00	-1.0 ± 0.4	0.040	GS598954	0	0	b	LinJ23_V3.1230	SHERP (dTPS0)	N.D.	
Lin194A4	-1.70	-0.8 ± 0.2	0.016	GS598955	0	1e-11	b	LinJ22_V3.1270	Aquaporin, putative (dTPS3)	N.D.	
Lin197D2*	-3.32	-1.7 ± 0.3	0.007	GS598956	0	0	b	LinJ07_V3.0150	Acyl-CoA dehydrogenase, mitochondrial precursor, putative (dTPS21, dTPS27)	+	-2.4 ± 0.2
								LinJ07_V3.0170	Maoc family protein	-	-1.1 ± 0.3
Lin206C10*	-1.99	-1.0 ± 0.3	0.032	GS598957	6e-69	1e-82	b	LinJ07_V3.0940	Cytochrome b5-like protein (dTPS0)	+	-1.7 ± 0.0
Lin206H7*	-1.90	-0.9 ± 0.2	0.015	GS598958	0	0	b	LinJ31_V3.1240	Vacuolar-type proton translocating pyrophosphatase 1, putative (dTPS0)	+	-3.4 ± 0.7
Lin208F2*	-2.10	-1.1 ± 0.3	0.026	GS598959	2e-86	3e-64	b	LinJ31_V3.1240	Vacuolar-type proton-translocating pyrophosphatase 1, putative	+	-3.4 ± 0.7
Lin208H10	-2.68	-1.4 ± 0.4	0.010	GS598960	0	0	a	LinJ18_V3.1070	Cysteine peptidase, Clan CA, family C2, putative (dTPS0)	+	-3.3 ± 0.1
								LinJ18_V3.1080	Vacuolar protein sorting complex subunit, putative (dTPS 0)	-	-1.2 ± 0.3
Lin219A10*	-1.89	-0.9 ± 0.1	0.004	GS598961	0	0	b	LinJ19_V3.0710	Glycosomal malate dehydrogenase (dTPS1, dTPS30)	+	-2.3 ± 0.0
Lin228D4*	-1.86	-0.9 ± 0.2	0.014	GS598962	0	0	a	LinJ19_V3.0090	Fibrillarin, putative (dTPS0)	+	-4.1 ± 0.8
Lin229E6	-9.63	-3.3 ± 0.2	0.002	GS598963	0	0	a	**LinJ06_V3.1320**	**Pteridine transporter, putative (dTPS0)**	+	-2.1 ± 0.3
								**LinJ06_V3.1330**	**Coproporphyrinogen III oxidase, putative (dTPS33)**	+	-5.8 ± 0.1
Lin231G4	-1.73	-0.8 ± 0.2	0.013	GS598964	0	0	b	LinJ31_V3.1240	Vacuolar-type proton translocating pyrophosphatase 1, putative (dTPS0)	+	
								LinJ31_V3.1250	Hypothetical protein, unknown function	N.D.	
Lin234C9	-1.77	-0.8 ± 0.3	0.038	GS598965	0	0	b	LinJ20_V3.1220	Cysteine peptidase, Clan CA, family C2, putative (dTPS41)	+	-3.3 ± 0.1
Lin242E2	-2.85	-1.5 ± 0.4	0.025	GS598966	0	0	b	**LinJ31_V3.2370**	**3'-nucleotidase/nuclease, putative (dTPS4, dTPS29)**	+	-4.6 ± 0.4
								**LinJ31_V3.2380**	**3'-nucleotidase/nuclease precursor, putative (dTPS4, dTPS29)**	+	-2.7 ± 0.6
Lin252B11	-2.12	-1.1 ± 0.3	0.028	GS598967	0	0	b	LinJ17_V3.170/200	Elongation factor 1αg(dTPS14, dTPS39, dTPS44p	N.D.	
Lin265E2	-1.89	-0.9 ± 0.3	0.042	GS598968	1e-165	0	b	**LinJ29_V3.1880**	**Paraflagellar rod protein 1D, putative (dTPS0)**	N.D.	
Lin270H10*	-1.96	-1.0 ± 0.2	0.010	GS598969	0	0	b	LinJ31_V3.1150	Monoglyceride lipase, putative (dTPS0)	+	-1.9 ± 0.0
Lin271C2*	-1.9	-0.9 ± 0.3	0.043	GS598970	0	0	b	LinJ28_V3.0090	Adenylate cyclase-like protein (dTPS9, dTPS12, dTPS23, dTPS24)	+	-2.3 ± 0.0
Lin285H1	-2.12	-1.1 ± 0.2	0.012	GS598971	0	0	b	**LinJ36_V3.6550**	**Glucose transporter lmgt2, putative (dTPS47)**	+	-8.1 ± 1.1
								**LinJ36_V3.6560**	**Glucose transporter, putative (dTPS47)**	+	-6.3 ± 1.4
Lin294G4*	-2.00	-1.0 ± 0.2	0.013	GS598972	0	0	b	LinJ31_V3.1640	Dipthine synthase, putative	-	
								LinJ31_V3.1660	Putative 3-ketoacyl-CoA thiolase-like protein (dTPS0)	+	-3.6 ± 0.5

This table contains clones that map against down-regulated genes (not hypothetical or unknown) with the combined effect of temperature increase and pH decrease (TPS). The features described are: clone number; F; base-two logarithmic scale F and SD values; *p*; GenBank GSS accession numbers; e-values; Def. according to mapping outcomes *a*, *b *or *c *(see brief explanation in the text); Id.; annotated gene function (codes for Additional file 4: Figure S4,); qRT-PCR. When a given clone overlaps with more than one annotation, stage-specific regulation is only demonstrated if the qRT-PCR result is positive (+). The asterisk indicates that the clone contains more gene sequences that have not been checked by qRT-PCR. Genes in bold are also down-regulated under TS.

**Table 3:** Up-regulated genes after temperature increase up to 37°C (day 4) in *L. infantum*.

*Clone*	*F*	*Log*_2_*F ± SD*	*P*	*GenBank GSS*	*e-value*		*Def*.	*Id*.	*Annotated Gene Function*	*qRT-PCR*	
					*Fw*	*Rv*				+/-	F ± SD
Lin11D7	2.37	1.2 ± 0.1	0.004	GS598854	-	0	c	**LinJ31_V3.0460**	**Amastin, putative**	+	
Lin17C6	1.92	0.9 ± 0.1	0.006	GS598973	0	0	b	LinJ36_V3.0640	Delta-8 fatty acid desaturase-like protein	N.D.	
Lin19D1	1.88	0.9 ± 0.3	0.028	GS598855	3e-18	0	b	**LinJ08_V3.0680**	**Amastin-like protein**	N.D.	
								**LinJ08_V3.0690**	**Amastin-like protein**	N.D.	
Lin33G2	2.29	1.2 ± 0.8	0.046	GS598857	6e-118	6e-118	b	**LinJ34_V3.4370**	**Amastin-like surface protein, putative**	N.D.	
Lin70F5	2.03	1.0 ± 0.4	0.045	GS598974	0	0	b	LinJ36_V3.7290	Delta-8 fatty acid desaturase-like protein	N.D.	
Lin77H8	2.89	1.5 ± 0.4	0.022	GS598975	3e-175	0	b	**LinJ08_V3.0690**	**Amastin-like protein**	N.D.	
Lin86H7	2.03	1.0 ± 0.2	0.005	GS598867	0	2e-101	b	**LinJ08_V3.0700**	**Amastin-like protein**	+	6.8 ± 0.9
Lin87H2	1.89	0.9 ± 0.1	0.007	GS598868	3e-15	3e-33	b	**LinJ08_V3.0690**	**Amastin-like protein**	N.D.	
Lin89D9	1.70	0.8 ± 0.3	0.040	GS598869	0	0	b	**LinJ21_V3.0770**	**ATP-binding cassette sub-family E, putative**	N.D.	
Lin90B6	1.95	1.0 ± 0.3	0.032	GS598976	0	0	a	**LinJ30_V3.0640**	**Ribosome biogenesis regulatoy protein (RRS1), putative**	+	1.9 ± 0.2
								LinJ30_V3.0650	Histidyl-tRNA synthetase, putative	N.D.	
								LinJ30_V3.0660	Hypothetical protein, conserved	N.D.	
Lin91B12	1.75	0.8 ± 0.1	0.003	GS598872	0	0	b	**LinJ34_V3.2660**	**Amastin-like surface protein**	N.D.	
Lin100B2	1.84	0.9 ± 0.3	0.034	GS598977	0	9e-27	b	LinJ27_V3.2500	Glycosomal phosphoenolpyruvate carboxykinase	N.D.	
Lin104B11	1.77	0.8 ± 0.2	0.022	GS598978	0	0	b	LinJ04_V3.0570	Spermidine synthase 1, putative	N.D.	
Lin104C10	1.82	0.9 ± 0.2	0.015	GS598979	0	0	b	**LinJ08_V3.1320**	**Amastin-like protein**	N.D.	
Lin106A1	2.43	1.3 ± 0.0	0.000	GS598980	0	0	c	LinJ06_V3.1200	Hypothetical protein, conserved	N.D.	
								**LinJ31_V3.0590**	**Amino acid transporter aATP11, putative**	+	2.4 ± 0.3
Lin109B3	1.89	0.9 ± 0.2	0.024	GS598981	0	0	b	LinJ21_V3.2130	Centromere/microtubule binding protein (cbf5), putative	N.D.	
Lin113C3	2.99	1.6 ± 0.3	0.010	GS598876	3e-74	0	a	**LinJ14_V3.1440**	**Pteridine transporter**	+	2.5 ± 0.3
								**LinJ14_V3.1450**	**Myo-inositol-1-phosphate synthase**	+	4.2 ± 0.1
Lin137A10	1.98	1.0 ± 0.3	0.039	GS598982	0	0	b	LinJ24_V3.1230	Hypothetical protein, conserved	N.D.	
								**LinJ24_V3.1240**	**Translation factor SUI1, putative**	+	1.8 ± 0.1
Lin146E3	2.52	1.3 ± 0.3	0.043	GS598882	0	0	b	**LinJ31_V3.0590**	**Amino acid transporter aATP11, putative**	+	2.4 ± 0.3
Lin162E6	1.92	0.9 ± 0.3	0.044	GS598983	0	0	a	LinJ14_V3.1430	Hypothetical protein, conserved	N.D.	
								**LinJ14_V3.1440**	**Pteridine transporter**	+	2.5 ± 0.3
								**LinJ14_V3.1450**	**Myo-inositol-1-phosphate synthase**	+	4.2 ± 0.1
Lin168A2	1.87	0.9 ± 0.2	0.017	GS598984	1e-78	0	b	LinJ22_V3.0670	Hypothetical protein	N.D.	
								**LinJ22_V3.0680**	**3'a2rel-related protein**	+	3.5 ± 0.6
Lin175D6	2.20	1.2 ± 0.4	0.023	GS598985	0	0	b	**LinJ31_V3.0460**	**Amastin, putative**	+	4.7 ± 1.2
Lin185A10	2.04	1.0 ± 0.3	0.036	GS598986	0	0	a	LinJ28_V3.0620	MAP kinase, putative	N.D.	
Lin185D7	1.75	0.0 ± 0.2	0.020	GS598987	2e-161	0	b	LinJ17_V3.0200	Elongation factor 1-alpha	N.D.	
Lin188H2	3.20	1.7 ± 0.6	0.042	GS598988	0	0	c	**LinJ08_V3.0680**	**Amastin-like protein**	N.D.	
Lin194E2	1.79	0.8 ± 0.2	0.025	GS598989	-	0	c	**LinJ08_V3.0710**	**Amastin-like protein**	+	6.8 ± 0.9
Lin206B6	2.08	1.0 ± 0.3	0.035	GS598990	7e-19	0	b	**LinJ22_V3.0680**	**3'a2rel-related protein**	+	3.5 ± 0.6
Lin207A1	1.84	0.9 ± 0.2	0.015	GS598991	0	0	b	LinJ17_V3.0170	Elongation factor 1-alpha	N.D.	
								LinJ17_V3.0180	Elongation factor 1-alpha	N.D.	
Lin210C4	1.71	1.8 ± 0.1	0.030	GS598893	0	0	b	**LinJ08_V3.0690**	**Amastin-like protein**	N.D.	
Lin224G2	1.70	0.8 ± 0.2	0.014	GS598895	0	0	b	**LinJ08_V3.0720**	**Amastin-like protein**	N.D.	
Lin235G8	2.20	1.1 ± 0.2	0.002	GS598896	0	0	b	**LinJ08_V3.1320**	**Amastin-like protein**	N.D.	
Lin245E2	2.05	1.0 ± 0.3	0.032	GS598897	0	0	b	**LinJ22_V3.0680**	**3'a2rel-related protein**	+	3.5 ± 0.6
Lin274G6	1.84	0.9 ± 0.2	0.012	GS598992	0	0	b	**LinJ08_V3.0680**	**Amastin-like protein**	N.D.	
								**LinJ08_V3.0690 **	**Amastin-like protein**	N.D.	
Lin275A8	2.10	1.1 ± 0.1	0.003	GS598900	0	4e-168	b	**LinJ08_V3.0720**	**Amastin-like protein**	N.D.	
Lin282B6	2.08	1.0 ± 0.4	0.042	GS598993	0	0	b	LinJ03_V3.0960	Elongation initiation factor 2 alpha subunit, putative	N.D.	
Lin294A11	1.72	0.8 ± 0.1	0.001	GS598902	0	0	b	**LinJ08_V3.1320**	**Amastin-like protein**	N.D.	
Lin295D9	2.99	1.6 ± 0.4	0.020	GS598903	0	0	b	**LinJ34_V3.1720**	**Amastin-like surface protein, putative**	N.D.	

This table describes clones that contain up-regulated genes under the sole influence of temperature increase (TS) that do not map with hypothetical or unknown genes. The features described are: clone number; fold change (F); base-two logarithmic scale F and standard deviation (SD) values; p-value (*p*); GenBank GSS accession numbers; e-values of forward (Fw) and reverse (Rv) end mappings against BLAST; clone definition (Def.) according to mapping outcomes *a*, *b *or *c *(see brief explanation in the text); GeneDB identifiers (Id.), the corresponding annotated gene functions; qRT-PCR results. When a given clone overlaps with more than one annotation, stage-specific regulation is only demonstrated if the qRT-PCR result is positive (+). Genes in bold are also up-regulated under TPS.

**Table 4:** Down-regulated genes after temperature increase up to 37°C (day 4) in *L. infantum*.

*Clone*	*F*	*Log*_2_*F ± SD*	*P*	*GenBank GSS*	*e-value*		*Def*.	*Id*.	*Annotated Gene Function*	*qRT-PCR*	
					*Fw*	*Rv*				+/-	F ± SD
Lin9E5	-1.77	-0.8 ± 0.3	0.033	GS598994	4e-131	0	b	LinJ35_V3.1150	Oligosaccharyl transferase-like protein	N.D.	
Lin40G12	-1.97	-1.0 ± 0.2	0.008	GS598916	2e-161	0	b	LinJ23_V3.1550	Hypothetical protein, unknown function	N.D.	
								**LinJ23_V3.1560**	**Lathosterol oxidase-like protein**	+	5.0 ± 0.7
Lin49B7	-4.38	-2.1 ± 0.0	0.000	GS598995	0	4e-64	a	**LinJ34_V3.4160**	**Phosphatidylinositol-3-kinase (tor2)-like protein**	N.D.	
Lin 60B1	-2.41	-1.3 ± 0.4	0.025	GS598996	0	4e-162	c	LinJ36_V3.7040	Hypothetical protein, conserved	N.D.	
								**LinJ31_V3.2370**	**3'-nucleotidase/nuclease, putative**	+	7.2 ± 1.0
Lin63F3	-2.23	-1.2 ± 0.4	0.043	GS598997	0	0	a	**LinJ36_V3.6550**	**Glucose transporter lmgt2, putative**	+	6.1 ± 0.8
								**LinJ36_V3.6560**	**Glucose transporter, putative**	+	6.1 ± 0.8
Lin84E8	-2.26	-1.2 ± 0.2	0.007	GS598998	0	0	a	**LinJ31_V3.2370**	**3'-nucleotidase/nuclease, putative**	+	7.2 ± 1.0
								**LinJ31_V3.2380**	**3'-nucleotidase/nuclease precursor, putative**	+	7.2 ± 1.0
Lin85H1	-1.74	-0.8 ± 0.2	0.025	GS598999	1e-57	1e-20	b	LinJ30_V3.3440	CAS/CSE importin domain protein, putative	N.D.	
Lin93H3	-2.26	-1.2 ± 04	0.030	GS599000	0	0	a	**LinJ31_V3.2370**	**3'-nucleotidase/nuclease, putative**	+	7.2 ± 1.0
								**LinJ31_V3.2380**	**3'-nucleotidase/nuclease precursor, putative**	+	7.2 ± 1.0
Lin97D1	-3.41	-1.7 ± 0.3	0.001	GS598931	0	0	a	**LinJ06_V3.1320**	**Pteridine transporter, putative**	+	2.3 ± 0.3
Lin98G10	-2.13	-1.1 ± 0.2	0.015	GS599001	0	0	b	LinJ30_V3.2770	Hypothetical protein, conserved	N.D.	
								**LinJ30_V3.2780**	**Superoxide dismutase, putative**	+	3.7 ± 0.0
Lin111C2	-2.74	-1.5 ± 0.1	0.002	GS599002	0	0	a	**LinJ09_V3.0650**	**Serine peptidase family S51, peptidase E, putative**	N.D.	
Lin150E4	-1.86	-0.9 ± 0.3	0.036	GS599003	0	8e-22	b	**LinJ13_V3.1060**	**Calmodulin, putative**	N.D.	
Lin155H12	-2.34	-1.2 ± 0.3	0.018	GS599004	0	0	a	**LinJ36_V3.0250**	**Peptidyl-prolyl cis-trans isomerase, putative**	N.D.	
Lin157D8	-2.27	-1.2 ± 0.1	0.002	GS599005	0	-	c	**LinJ31_V3.2380**	**3'-nucleotidase/nuclease precursor, putative**	+	7.2 ± 1.0
Lin179B4	-1.76	-0.8 ± 0.1	0.004	GS599006	0	0	b	LinJ07_V3.0030 LinJ07_V3.0040 LinJ07_V3.0050 LinJ07_V3.0060	Hypothetical protein, conserved Hypothetical protein, conserved Hypothetical protein, conserved	N.D. N.D. N.D. +	
									Alpha-adaptin-like protein		5.3 ± 0.4
Lin182F2	-2.23	-1.2 ± 0.1	0.008	GS598952	0	0	b	**LinJ25_V3.0740**	**Eukaryotic initiation factor 5a, putative**	N.D.	
								**LinJ25_V3.0750**	**Eukaryotic initiation factor 5a, putative**	N.D.	
Lin187C10	-4.72	-2.2 ± 0.4	0.013	GS598953	0	0	b	**LinJ06_V3.1320**	**Pteridine transporter, putative**	+	2.3 ± 0.3
Lin204A11	-1.76	-0.8 ± 0.3	0.038	GS599007	-	1e-165	c	**LinJ09_V3.0650**	**Serine peptidase, family S51, peptidase E, putative**	N.D.	
Lin210B7	-1.74	-0.8 ± 0.2	0.016	GS599008	0	0	a	LinJ32_V3.3690 LinJ32_V3.3700	DEAD/DEAH box helicase, putative	+ N.D.	3.3 ± 0.8
									Hypothetical protein, conserved		
Lin229E6	-3.30	-1.7 ± 0.3	0.005	GS598963	0	0	a	**LinJ06_V3.1320** **LinJ06_V3.1330**	**Pteridine transporter, putative** **Coproporphyrinogen III oxidase, putative**	+ +	2.3 ± 0.3 4.5 ± 0.6
Lin242E2	-2.37	-1.2 ± 0.4	0.039	GS599009	1e-137	0	a	**LinJ31_V3.2370** **LinJ31_V3.2380**	**3'-nucleotidase/nuclease, putative** **3'-nucleotidase/nuclease precursor, putative**	+ +	7.2 ± 1.0 7.2 ± 1.0
Lin255E12	-2.54	-1.3 ± 0.3	0.011	GS599010	0	0	b	LinJ28_V3.0210	Histone H2B variant	N.D.	

This table contains clones that map against down-regulated genes (not hypothetical or unknown) with the single effect of temperature increase (TS). The features described are: clone number; F; base-two logarithmic scale F and SD values; *p*; GenBank GSS accession numbers; e-values; Def. according to mapping outcomes *a*, *b *or *c *(see brief explanation in the text); Id.; annotated gene function; qRT-PCR. When a given clone overlaps with more than one annotation, stage-specific regulation is only demonstrated if the qRT-PCR result is positive (+). Genes in bold are also down-regulated under TPS.

**Table 5:** Differentially regulated genes after pH4.5 treatment (day 4) in *L. infantum*.

*Clone*	*F*	*Log*_2_*F ± SD*	p	*GenBank* *GSS*	*e-value*		*Def*.	*Id*.	*Annotated Gene Function*	*qRT-PCR*	
					*Fw*	*Rv*				+/-	F ± SD
Lin9E8	2.03	1.0 ± 0.1	0.003	GS599011	0	0	a	LinJ24_V3.0020	Clathin coat assembly protein, putative	+	7.6 ± 0.4
								LinJ24_V3.0030	Hypothetical protein, conserved	N.D.	
								LinJ24_V3.0040	60S ribosomal protein L17, putative	N.D.	
Lin10H12	2.26	1.2 ± 0.1	0.001	GS599012	0	0	a	LinJ31_V3.0860	Triacylglycerol lipase-like protein	N.D.	
								LinJ31_V3.0870	Lipase precursor-like protein	N.D.	
Lin21H10	2.46	1.3 ± 0.1	0.001	GS598910	0	0	b	LinJ26_v3.1670	Sphingolipid delta-4 desaturase, putative	N.D.	
Lin33G5	1.76	0.8 ± 0.0	0.000	GS599013				LinJ27_V3.1300	60S acidic ribosomal protein, putative	N.D.	
Lin37C10	2.74	1.5 ± 0.2	0.006	GS599014	0	0	b	LinJ33_V3.0280	RNA binding protein rggm, putative	N.D.	
Lin58C1	2.32	1.2 ± 0.2	0.001	GS599015	0	0	b	LinJ22_V3.1360	Hypothetical protein, unknown fuction	N.D.	
								LinJ22_V3.1370	60S ribosomal protein L14	N.D.	
								LinJ22_V3.1380	Dephospho-CoA kinase, putative	+	-2.9 ± 0.3
Lin63B7	1.83	0.9 ± 0.1	0.002	GS599016	1e-100	1e-103	b	LinJ15_V3.1200	60S acidic ribosomal protein P2	N.D.	
Lin66A8	2.28	1.2 ± 0.1	0.006	GS599017	0	0	a	LinJ22_V3.0470	Hypothetical protein, conserved	N.D.	
								**LinJ22_V3.0480**	**Ubiquitin-conjugating enzyme-like protein**	+	-3.1 ± 0.8
Lin80C3	3.15	1.7 ± 0.3	0.004	GS599018	0	0	b	LinJ28_V3.3250	Glucose-6-phosphate N-acetyltransferase	N.D.	
Lin 95F10	2.26	1.2 ± 0.1	0.002	GS599019	0	0	a	LinJ28_V3.2360	Ribosomal protein S29, putative	N.D.	
Lin100F8	2.12	1.1 ± 0.2	0.007	GS599020	0	2e-161	b	LinJ35_V3.3330	60S ribosomal protein L31, putative	N.D.	
								LinJ35_V3.3340	60S ribosomal protein L31, putative	N.D.	
Lin107C12	2.90	1.5 ± 0.2	0.001	GS599021	7e-130	5e-134	a	LinJ11_V3.1180	40S ribosomal protein S15a, putative	N.D.	
Lin122C5	1.93	0.9 ± 0.1	0.005	GS599022	0	0	b	LinJ30_V3.3770	CPSF-domain protein, putative	N.D.	
								LinJ30_V3.3780	60S acidic ribosomal protein P2, putative	N.D.	
								LinJ30_V3.3790	60S acidic ribosomal protein P2, putative	N.D.	
Lin135F6	2.63	1.4 ± 0.3	0.036	GS599023	0	0	b	LinJ29_V3.1920	40S ribosomal protein S15a, putative	N.D.	
								LinJ29_V3.1930	Hypothetical protein, conserved	N.D.	
Lin137A10	2.00	1.0 ± 0.2	0.019	GS599024	0	0	b	LinJ24_V3.1230	Hypothetical protein, conserved	N.D.	
								**LinJ24_V3.1240**	**Translation factor SUI1, putative**	+	-5.0 ± 0.6
Lin144F11	1.72	0.8 ± 0.2	0.032	GS599025	0	0	a	LinJ31_V3.2210	Prostaglandin F2α synthetase	N.D.	
Lin161C9	2.54	1.3 ± 0.1	0.003	GS599026	0	1e-177	b	LinJ26_V3.1670	Sphingolipid delta-4 desaturase, putative	N.D.	
Lin162A9	1.97	1.0 ± 0.2	0.024	GS599027	0	0	b	LinJ22_V3.0470	Hypothetical protein, conserved	N.D.	
								**LinJ22_V3.0480**	**Ubiquitin-conjugating enzyme-like protein**	+	-3.1 ± 0.8
Lin182F2	3.27	1.7 ± 0.2	0.009	GS599028	0	0	b	LinJ25_V3.0740	Eukaryotic initiation factor 5a, putative	N.D.	
								LinJ25_V3.0750	Eukaryotic initiation factor 5a, putative	N.D.	
Lin200H12	2.54	1.3 ± 0.1	0.005	GS599029	0	0	a	LinJ14_V3.1340	Hypothetical protein, unknown funcion	N.D.	
								LinJ14_V3. 1350	Ubiquitin/ribosomal protein S27a, putative	+	-4.8 ± 0.4
								LinJ14_V3.1360	Hypothetical protein, conserved	N.D.	
Lin218E3	1.82	0.9 ± 0.1	0.001	GS599030	0	0	b	LinJ31_V3.2210	Prostaglandin F2α synthase	N.D.	
Lin247D7	2.41	1.3 ± 0.4	0.018	GS599031	5e-109	0	a	LinJ28_V3.0090	Adenylate cyclase-like protein	+	-3.5 ± 0.6
								LinJ28_V3.0100	Hypothetical protein, conserved	N.D.	
								LinJ28_V3.0110	Proteasome beta 3 subunit, putative	N.D.	
Lin254A4	1.93	0.9 ± 0.2	0.009	GS599032	0	0	b	LinJ04_V3.1250	Actin	N.D.	
Lin254H7	1.73	0.8 ± 0.1	0.004	GS599033	0	0	b	LinJ04_V3.1250	Actin	N.D.	
Lin261F8	2.84	1.5 ± 0.2	0.007	GS599034	0	0	b	LinJ21_V3.1310	40S ribosomal protein S23, putative	N.D.	
Lin266F6	1.79	0.8 ± 0.1	0.009	GS599035	0	0	b	LinJ27_V3.0300	Acyl carrier protein, putative	N.D.	
Lin267B9	1.74	0.8 ± 0.2	0.010	GS599036	0	0	b	LinJ36_V3.0580	Hypothetical protein, conserved	N.D.	
								LinJ36_V3.0590	Ubiquitin-like protein, putative	+	-2.3 ± 0.0
								LinJ36_V3.0600	Cdc2-related kinase	N.D.	
Lin269B5	2.75	1.5 ± 0.2	0.002	GS599037	0	0	b	LinJ29_V3.2970	40S ribosomal protein S19-like protein	N.D.	
Lin282B6	2.44	1.3 ± 0.2	0.009	GS599038	0	0	a	LinJ03_V3.0960	Elongation initiation factor 2α subunit, putative	N.D.	
Lin290G8	1.80	0.8 ± 0.1	0.003	GS599039	0	0	a	LinJ17_V3.1520	Otubain cysteine peptidase, Clan CA. family C65, putative	N.D.	

Lin43G10	-5.36	-2.4 ± 0.3	0.007	GS599040	0	0	c	LinJ28_V3.3060	Heat-shock protein hsp70, putative	+	-2.1 ± 0.2
Lin130C5	-4.71	-2.2 ± 0.3	0.041	GS599041	0	0	b	LinJ36_V3.3170	Exosome complex exonuclease RRP41, putative	N.D.	
								LinJ36_V3.3180	Clathrin coat assembly protein-like protein	N.D.	
								LinJ36_V3.3190	Pre-mRNA branch-site protein p14	+	-4.7 ± 2.3
								LinJ36_V3.3200	Hypothetical protein, conserved	N.D.	
Lin173E11	-7.74	-3.0 ± 0.4	0.002	GS599042	6e-44	3e-147	b	LinJ36_V3.2280	ER-golgi transport protein erv25 precursor, putative	N.D.	
Lin177H3	-5.08	-2.3 ± 0.2	0.001	GS599043	0	4e-60	b	LinJ28_V3.3060	Heat shock protein hsp70, putative	+	-2.1 ± 0.2
Lin197E1	-2.53	-1.3 ± 0.1	0.007	GS599044	0	0	c	LinJ18_V3.0830	Periodic tryptophan protein 2-like protein	-	1.4 ± 0.4
								LinJ23_V3.1610	Acetyltransferase-like protein	+	-2.1 ± 0.2
Lin228H5	-7.90	-3.0 ± 0.4	0.012	GS599045	7e-196	0	b	LinJ21_V3.0310	Hexokinase, putative	N.D.	
Lin281H8	-2.01	-1.0 ± 0.1	0.001	GS599046	8e-136	2e-102	b	LinJ35_V3.1580	Metacaspase, putative	N.D.	
Lihsp70	-4.21	-2.0 ± 0.2	0.004	XM001470292	-	-	-	-	*L. infantum *hsp70 - DNA microarray control spot	+	-2.1 ± 0.2
Ldohsp70	-4.57	-2.2 ± 0.1	0.028	-	-	-	-	-	*L. donovani *hsp70 - DNA microarray control spot	N.D.	
Lmahsp70	-3.85	-1.9 ± 0.2	0.017	-	-	-	-	-	*L. major *hsp70 -DNA microarray control spot	N.D.	

This table contains clones that map against up- and down-regulated genes (not hypothetical or unknown) under pH decrease (PS). The features described are: clone number; F; base-two logarithmic scale F and SD values; *p*; GenBank GSS accession numbers; e-values; Def. according to mapping outcomes *a*, *b *or *c *(see brief explanation in the text); Id.; annotated gene function; qRT-PCR. When a given clone overlaps with more than one annotation, stage-specific regulation is only demonstrated if the qRT-PCR result is positive (+). Genes in bold are also up-regulated under TPS.

### Concomitant temperature increase and acidification (TPS) leads promastigotes toward AL

It has been stated that acidification and the simultaneous effect of temperature increase induce the differentiation of promastigotes to amastigotes [6,7]. In spite of the amastigote-like round cell morphology induced under these conditions, we have observed that in a fraction of the population flagella are not hidden (Figure 2). Nevertheless, it is important to take into account that we have performed the assays in standard medium in which promastigotes are cultured (RPMI supplemented with HIFBS) instead of media used for axenic amastigote culture such as Schneider's medium in order to avoid the effect of this factor and focus this study on pH and temperature influence.

**Figure 5:**
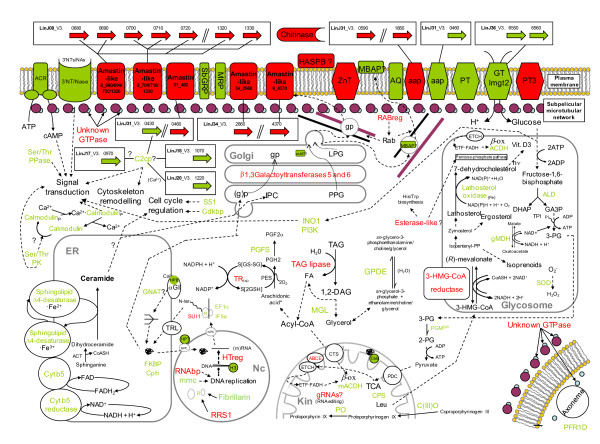
**Scheme representing differentially regulated genes under TPS and their subcellular localisation and/or functional relations**. Up-regulated genes are represented in red colour (Cy5) and down-regulated in green (Cy3). Further explanations in the *TPS expression profile *subsection, which is included in the *Results and Discussion *section.

We have observed that TPS-treated cells differentiate into AL after 4 days of stimulation (Figure 2), when control promastigotes are reaching the stationary-phase (Figure 1). As mentioned before, TPS-treated cells proliferate to a lesser extent than TS-treated ones due to the effect of pH decrease. Expression profiling by DNA microarrays has revealed a set of up- and down-regulated genes (Tables 1, 2, Additional file 3: Table S1 and S2) that are fully discussed below in the TPS expression profile section and illustrated in Figures 3 and 5. Taken as a whole, TPS induces promastigote differentiation to AL, as indicated by gp46 IFA and agreement with previous reports on the differential expression regulation of the following genes [13]: A2 gene and a set of amastin genes (up-regulated); 3'NT/Nase cluster and SbGRP encoding gene (down-regulated).

### TS alone leads to a TPS-like expression profile

**Figure 6:**
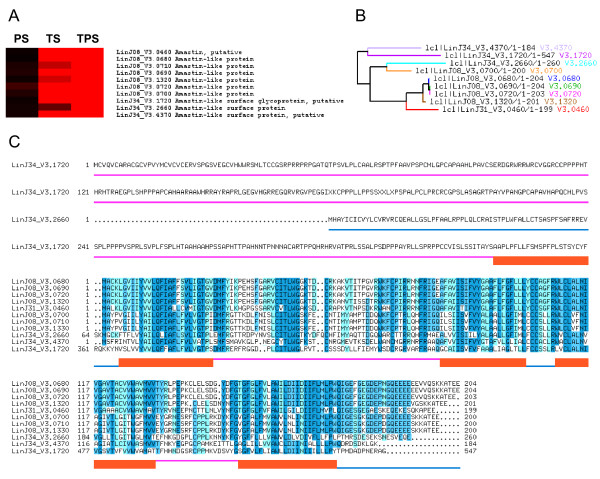
**Amino acid sequence and domain analysis of amastin genes found to be differentially regulated under TPS and TS**. (A) MEV comparison of differential regulation under TPS, TS and PS. (B) Sequence similarity tree representing distances between amastin genes found to be up-regulated under TPS and TS. (C) Amino acid sequences were aligned with CLUSTALW2 software. The darker the position is highlighted the more conserved the residue is. The boundaries of inner, transmembrane and outer domain sequences predicted with TMHMM 2.0 software are represented below sequence alignments.

TS-stimulated differentiation processes have been studied only from a morphological point of view in *L. infantum*, but not at the differential gene expression level. For the first time, we have described in this research the influence of TS on the whole transcriptome of the parasite (Tables 3, 4, Additional file 3: Table S3 and S4). Analogies between TPS and TS expression profiles have been observed, namely in the differential regulation of the following genes (Tables 1, 2, 3 and 4, in bold): up-regulation of 3'a2rel-related protein, some amastin superfamily genes (see Figure 6 and *Amastin Superfamily *subsection below), ribosome biogenesis regulatory protein (RRS1), myo-inositol-1-phosphate synthase (INO1), amino acid transporter aATP11, three conserved hypothetical protein genes and eight clones that do not map with any annotated genic sequence; and down-regulation of 3'NT/Nase, pteridine transporter (PT) LinJ06_V3.1320, glucose transporters (GT), paraflagellar rod protein 1D (PFR1D), superoxide dismutase (SOD), phosphatidylinositol-3-kinase (tor2)-like (PI3K), peptidyl-prolyl cis-trans isomerase (FKBP) LinJ36_V3.0250, calmodulin, lathosterol oxidase, one hypothetical protein of unknown function, six conserved hypothetical proteins and seven clones that do not map with any annotated gene. These clones unmapped with genes strongly suggest that gene annotations on the *L. infantum *genomic sequence are incomplete, thus highlighting the advantages of using shotgun genomic DNA microarrays and the subsequent genomic library. As pointed out above, TPS-induced *in vitro *stimulation results in a differentiation process that resembles the differentiation of promastigotes to amastigotes inside the phagocytes of the mammalian host. Despite TS itself inducing analogous differentiation events and TS-treated cells being called AL [9], the A2 gene is not up-regulated (Table 3), all cells show a large flagellum and gp46 IFA is positive under TS (Figure 2). Nevertheless, SAM and the subsequent HCL-ST analysis of clones with regard to their fold-change values have revealed significant similarities between the transcriptome under TPS and TS (Figure 4). Genes of known function with the same expression pattern under TPS and TS are highlighted in bold in Tables 1, 2, 3 and 4 (those of unknown function in Additional file 3). The specific regulation of these genes by temperature increase is directly correlated to the differentiation to the amastigote stage. To sum up, even though TS-treated cells are not differentiated to the same extent as TPS, the similarities found between TPS and TS expression profiles when contrasted with the PS profile have led us to conclude that temperature has a greater influence than pH on the differentiation process leading up to the amastigote stage.

### Acidification (PS) contributes little to the differentiation process

Some authors have considered that the induction of metacyclogenesis in promastigotes by acidic pH is a response common to a variety of *Leishmania *species [21,22]. Although there is no evidence concerning the metacyclic status of such promastigotes except for morphological considerations, proliferation seems to be inhibited by the single effect of acidification (pH 4.5-5.5) after 48 h according to [5] and our own observations. Figure 1 shows that promastigote growth is limited under these conditions, which is consistent with the generation time increase previously observed at pH 4.5 [11]. After an intermediate-term exposure to PS (day 4), two cell morphologies were observed: round and promastigote-like, both with emerging flagellum (Figure 2). Moreover, lack of A2 gene up-regulation (control gene spotted in each microarray) and an atypical gene expression profile have been found. There are some similarities in the expression profiles of TPS-obtained AL and PS-treated cells: up-regulation of triacylglycerol (TAG) lipase (TGL), translation factor SUI (TFSUI1)-also up-regulated under TS-, ubiquitin conjugating enzyme-like and five clones that do not map with any annotated gene; down-regulation of a conserved hypothetical protein and a gene still to be annotated; and the previous finding of an amastigote-specific protein induced by pH decrease [11]. In addition, 60S acidic ribosomal protein P2, 60S ribosomal protein L31 [23], ribosomal protein S29 and RNA binding protein rggm [24] are up-regulated in intracellular amastigotes according to Serial Analysis of Gene Expression (SAGE), which is due to PS (Table 5). In spite of this, the vast majority of differentially regulated genes under PS (Tables 5, Additional file 3: Table S5 and S6) have not been found to match up with those of the TPS and TS profiles. In fact, SAM output of differentially modulated genes between PS, TS and TPS was analysed by HCL-ST, which revealed that the most distant experimental group is PS (Figure 4). Moreover, there are opposite gene expression regulation events between TPS and PS: down-regulation under TPS and up-regulation under PS of glucose-6-phosphate N-acetyltransferase gene (GNAT), sphingolipid Δ4-desaturase, prostaglandin F synthetase (PGFS), eukaryotic translation initiation factor 5a (eIF5a) and two clones that do not map with any annotated sequence. Furthermore, there is also a lack of resemblance with the metacyclic promastigote profile [25], except for the up-regulation of 60S acidic ribosomal protein LinJ27_V3.1300 and some clones probably containing contig 957 guide RNA (gRNA) sequence (Additional file 3: Table S7 and S8). Considered together with the HCL-ST analysis of gene expression, these data suggest that intermediate-term exposure of promastigotes to PS leads to forms with features that do not match with any of the stages of the parasite's biological cycle (Figure 4) except for explained coincidences. Consequently, although pH has a role in differentiation, temperature is more relevant.

### TPS-induced expression profile

#### Overview: Gene Ontology term annotations

All genes identified as potentially regulated under these conditions were re-annotated with BLAST2GO to describe globally the influence of TPS on the *L. infantum *transcriptome. Despite the useful overview provided by this analysis, which has revealed the functions of some hypothetical proteins, specific genes of trypanosomatid parasites like amastins or A2 cannot be correlated to any of the terms included in the database, as they do not show any known activity. The analysis of GO molecular function terms associated with a TPS-induced profile (Figure 3A and 3B) indicates an increase in galactosyltransferase (also observed by SAGE [24]), nucleoside triphosphatase activity and amine transmembrane transporter activities and a decrease in transcripts with associated GO molecular function term annotations such as cyclase, protein kinase and calcium-related cysteine peptidase (all related to signal transduction processes), translation initiation and elongation factor and oxidoreductase activities related to electron transport. These findings at the molecular function level can be clearly described at the biological process GO term level (Figure 3C and 3D): the down-regulation of several genes related to the regulation of translational initiation, elongation and post-translational modification indicates that protein biosynthesis and modification is more active in stationary-phase promastigotes rather than in AL. The same occurs with signal transduction, prostaglandin F and porphyrin biosynthesis. Genes related to biopolymer and lipid metabolic processes, glycosylation of proteins and regulation of cellular processes are up-regulated in TPS-induced AL. Nevertheless, there are some common biological process GO terms that are up- and down-regulated simultaneously, but this refers to different genes in each case: electron transport activity is referred mainly to cytochrome b5 reductase at CC (it is involved in electron transport to the sphingolipid-Δ4-desaturase reaction), while trypanothione reductase (TR) and the ABC transporter subfamily E (ribonuclease L-inhibitor) gene (ABCE) are both related to the same term; the amino acid transport term is also present at both stages, but nucleotide sequences of the corresponding aminoacid permeases are different, which suggests that a different transporter is used in each stage.

The resulting microarray data for the TPS-induced AL expression profile analysis is discussed in the next subsections according to the iterative HCL-ST (Figure 4) and BLAST2GO-based analyses (Figure 3 and Additional file 4). Moreover, it is illustrated schematically in Figure 5 with regard to the leishmanial surface, cytoskeleton, secretory pathway, metabolic and signalling processes. Direct acyclic graphs (DAGs) (Additional file 4) have been associated with genes shown in Tables 1, 2, 3, 4 and 5 by means of custom codes assigned in brackets after the name of each gene annotation.

#### Amastin superfamily

Several proteins from the uncharacterised surface amastin superfamily have been shown to be up-regulated basically in the amastigote stage of *Trypanosoma cruzi, L. major *and *L. infantum *[26,27]. The microarray-based transcriptome analysis contained in this study has revealed that eleven amastin genes are up-regulated under TPS, ten out of these under TS but none under PS. In fact, SAM highlights significant differences in the expression pattern of the eleven amastin genes and the subsequent amastin HCL-ST analysis supports the same expression pattern except for LinJ34_V3.2660 (Figure 6A). Furthermore, these amastin genes have been reported to be up-regulated in intracellular and axenic amastigotes by microarrays [28] and SAGE [24]. According to TMHMM predictions, these amastins contain 4 transmembrane, 3 inner and 2 outer domains, except for LinJ34_V3.1720, which contains a 300 amino acid long N-terminal (N-ter) region followed by an additional short transmembrane domain. Outer domains are variable among amastin superfamily members, although they are very similar in a given amastin group or class (Figure 6B and 6C). Amastins LinJ08_V3.0680/0690 and LinJ08_V3.0700/0710 were previously found to be up-regulated in metacyclic promastigotes [25], which supports that amastin genes are not amastigote markers. The expression rate of these genes increases as the life cycle progresses.

#### A2-A2rel cluster

A2 gene cluster was first identified in *L. donovani*, where A2 transcripts are abundant in amastigotes but hardly detectable in promastigotes [29]. These molecules were proposed as virulence factors that enhance survival of the amastigote inside the macrophage [30]. It has been suggested that a balance between A2 and A2rel proteins is required for the parasite's survival [31]. *L. donovani *and *L. infantum *A2 genes were spotted onto the microarrays as amastigote-specific control genes. We have observed an increase in the corresponding transcript levels under TPS in the hybridisation analysis (Table 3). In addition, our results indicate that TPS and TS elicit the up-regulation of 3'a2rel-related transcripts in *L. infantum*.

#### DNA repair and replication, gene expression and secretory pathway

A member of minichromosome maintenance complex protein (*mmc*) 2/3/5 family (PFAM annotation PF00493) is down-regulated and an RNA binding protein (RNAbp) up-regulated in TPS-induced AL. *mmc *and RNAbp are involved in DNA replication according to GO biological process annotation. The histone H3 gene is up-regulated under TPS, as well as in intracellular amastigotes according to SAGE [23]. It is also involved in nucleosome assembly and DNA repair according to GO annotation.

With regard to gene expression and protein processing, a hypothetical transcription regulator gene (HTreg) and RRS1 are up-regulated under TPS, while nucleolar fibrillarin is down-regulated. RRS1 is also up-regulated under TS. TFSUI1 is up-regulated under TPS, TS and PS (see above). The elongation factor 1α (EF1α) is down-regulated in both TPS-induced AL and in intracellular *L. major *promastigotes as previously reported [32] and IF5a is also down-regulated by TPS, suggesting a different translation regulation mechanism under TPS selective pressure. Peptidyl-prolyl cis-trans isomerases FKBP and cyclophilin (Cph) are also down-regulated in TPS-generated AL and FKBP under TS. FKBP and Cph are involved in protein folding inside the endoplasmic reticulum (ER) and we had already found the down-regulation of both genes in metacyclic peanut lectin non-agglutinating promastigotes [25]. As a consequence, Cph and FKBP gene expression decreases throughout the parasite's life cycle. In addition, a hypothetical protein related to calcium ion and protein binding GO molecular functions (αGII-HPB) localises to the dimeric α-glucosidase-II complex according to GO cellular component analysis and is down-regulated at the level of transcript under TPS. GNAT is also down-regulated and is involved in protein oligosaccharide biosynthesis inside the ER lumen, possibly in the glucosylation/deglucosylation cycle. We have found that a Rab GTPase regulator protein (RABreg, see further explanation in the *Cytoskeleton remodelling *subsection) is up-regulated under TPS, probably promoting vesicle transport from Golgi apparatus. In addition, β-1,3-galactosyltransferase-5/6 carries out galactosylation of proteophosphoglycan and lypophosphoglycan if required. These genes have been found to be up-regulated under TPS, as it was also reported for metacyclic peanut lectin non-agglutinating promastigotes [25] and intracellular amastigotes according to SAGE [24].

#### Energetic metabolism

TPS-obtained AL down-regulate transcript levels of two glycolitic genes: fructose-1,6-bisphosphate aldolase (ALD) and 2,3-bisphosphoglycerate-independent phosphoglycerate mutase (PGM^BPI^). This agrees with the down-regulation of PGM^BPI ^protein in *L. donovani *[33] and transcripts in *L. infantum *(unpublished data) mature intracellular amastigotes. The ALD gene was also found to be down-regulated at the post-transcriptional level in *L. mexicana *mature amastigotes [17] and at the protein level in immature *L. donovani *amastigotes. By contrast, ALD protein is up-regulated in *L. donovani *mature intracellular amastigotes [33], which differs from TPS-induced AL. Down-regulation of both genes is consistent with high energy requirements in the promastigote stage. ALD and PGM^BPI ^are independent of catabolite regulation and are located in the glycosome and the cytosol respectively. Inhibition of glycolysis by ALD and PGM^BPI ^down-regulation is consistent with the down-regulation of two GTs under TPS and TS. Both genes are located in tandem in chromosome 36 and custom CLUSTALW2 alignments (Additional file 5) illustrate that their sequences are identical except for N-terminal regions (N-ter) of coded peptides. qRT-PCR analysis is consistent with the up-regulation of both GT, as well as the up-regulation of GT *lmgt2 *in *L. mexicana *[17] and *L. major *[32] intracellular amastigotes. NAD^+^ supply for glyceraldehyde-3-phosphate dehydrogenase (GAPDH) reaction is assured by the up-regulation of the glycosomal malate dehydrogenase (gMDH) gene at CC with respect to TPS. The mitochondrial precursor of acyl-CoA dehydrogenase LinJ07_V3.0150 gene (mACDH) is also down-regulated under TPS, which suggests that *β*-oxidation (*β*-ox) of fatty acids (FA) is activated under such conditions, as well as glucose uptake and glycolysis.

ABCE is up-regulated under TPS and is involved in electron transport. In fact, the only ABCE family member studied to date is a multifunctional protein that includes a metal-binding domain (PF04068) adjacent to the 4Fe-4S binding domain (PF00037), as well as two ATP-binding sites/ATPase domains typical of ABC proteins (PF00005) but it lacks transporter domains. This kind of protein has been found in pluricellular eukaryotes but not in yeast and binds directly to RNase L to prevent it from binding 5'-phosphorylated 2',5'-linked oligo-adenylates [34]. The biological role of ABC and the meaning of its up-regulation at the level of transcript in TPS-obtained AL are still unknown in *Leishmania *spp. ABCE localises to the kinetoplast according to GO cellular component annotation.

#### Lipid metabolism

TGL is post-transcriptionally up-regulated under TPS and is involved in *sn*-2 and *sn*-3 hydrolysis of TAGs. CoA can be incorporated in the released FA and enter *β*-ox, where mACDH and 3-ketoacyl-CoA thiolase (thiolase I) are down-regulated. On the other hand, monoglyceride lipase (MGL) is down-regulated under TPS. Another gene with the same regulation pattern is a hypothetical protein with a glycerolphosphodiester phosphodiesterase (GPDE) function (GO molecular function analysis), which is related to glycerol derivative metabolism. An additional destination for FA is the sphingolipid biosynthesis pathway, in which sphingolipid-Δ4-desaturase oxidises dihydroceramide to ceramide in the presence of O_2 _and Fe as cofactor (Fe^3+ ^reduced to Fe^2+^) [35]. The sphingolipid-Δ4-desaturase gene is down-regulated under TPS and is located in the ER membrane (GO cellular component analysis), as well as the cytochrome b5 reductase gene (cyt b5 reductase), which provides reduction power for desaturases through cytb5. After ceramide biosynthesis, a molecule of phosphoinositol can be added inside the Golgi apparatus resulting in inositol phosphoceramide (IPC) for anchoring inositol derivatives. Saturated acyl groups are also the precursors of polyinsaturated fatty acids like arachidonic acid, from which prostaglandins are derived. PGFS is down-regulated under TPS, while TR is up-regulated. The reaction prior to PGFS is catalysed by prostaglandin peroxide synthase (PES) and requires trypanothione in its reduced state. TR regenerates reduced molecules for PES reaction, as well as for many other redox processes. Thus, increases in PGFS and TR at different stages is not a contradictory fact, given the wide functional spectrum of the latter. PGFS is also up-regulated in procyclic promastigotes with respect to metacyclics [25] and has been associated with vector competence of procyclic promastigotes. Taken together, these data confirm that PGFS levels diminish throughout differentiation. Finally, 1,2-DAG can enter inositolphospholipid metabolism, where PI3K is down-regulated under TPS and TS. As PGFS, mACDH, thiolase I, sphingolipid Δ4-desaturase and PI3K are down-regulated, the destination of 1,2-DAG and FA excess generated by gene up-regulation of TAG lipase remains unclear.

The gene coding for 3-hydroxymethylglutaryl-CoA (HMG-CoA) reductase (HMGCR) is up-regulated under TPS. This is the rate-limiting step of sterol and isoprenoid biosynthesis. In view of this result, ergosterol biosynthesis may be increased in AL. HMGCR localises to the glycosome in *Leishmania *spp., where leucine (in trypanosomatids [36]) must be carried for priming steroid biosynthesis (reviewed in [37]). In spite of HMGCR increase in TPS-induced AL, the lathosterol oxidase gene has been found previously to be down-regulated in intracellular amastigotes ([28] and unpublished custom data) and the analysis reported in this study has revealed that the down-regulation of this gene is due to the specific influence of TPS and TS. Lathosterol oxidase yields 7-dehydrocholesterol. *Leishmania *parasites lack the enzyme cholesterol:NADP^+^ Δ^7^-oxidoreductase, that catalyses the conversion of 7-dehydrocholesterol into cholesterol (KEGG database for *L. major *[38]). Cholesterol functions are performed by ergosterol in these organisms. A question arises about the destination of 7-dehydrocholesterol in promastigotes (CC). Vitamin D3 (cholecalciferol) is synthesised by exciting 7-dehydrocholesterol with a photon (hν), that according to our gene expression results may occur inside the insect vector's gut, where promastigotes are undergoing a developmental process. Obviously, the biological meaning of this fact still remains unclear.

#### Porphyrin biosynthesis

The prosthetic heme group is required for many electron transport chain proteins (cytochromes), including cyt b5. *Leishmania *spp. does not have the ability to perform porphyrin biosynthesis *de novo*, because it lacks δ-aminolevulinate synthase, porphobilinogen synthase and deaminase and uroporphyrinogen decarboxylase. These parasites are able to acquire protophorphyrinogen IX or heme group directly from the mammalian host. Moreover, coproporphyrinogen III, protoporphyrinogen oxidases (C(III)O, PO) and a ferrochelatase-like protein are annotated in the genome of the parasite, which highlights its ability to perform heme group biosynthesis from the substrate coproporphyrinogen III. Interestingly, C(III)O and PO are located in tandem in chromosome 6 and are down-regulated under TPS according to our microarray hybridisation results. In fact, CC mimic the environment inside the gut of the phlebotominae sand-fly, where the parasites cannot acquire heme or protoporphyrin IX. In spite of this, we have not found ferrochelatase gene (located in chromosome 17) to be differentially regulated under the experimental conditions assayed. This observation is additional evidence backing the hypothesis of gene organization in DGCs depending on the post-transcriptional regulation in *Leishmania *spp (reviewed in [39]).

#### Redox homeostasis and oxidative stress

TR catalyses the reduction of reactive oxygen radical superoxide anion to hydrogen peroxide and is responsible for maintaining the glutathione orthologue trypanothione in its reduced form, essential for redox defence systems in trypanosomatids. For this reason, TR would be a useful chemotherapeutic target [40]. TRs are members of the NADPH-dependent flavoprotein oxidoreductase family and are structurally and mechanistically related to gluthathione reductase [41]. The disruption of the TR gene in *Leishmania *decreases the ability to survive oxidative stress inside macrophages [41]. The up-regulation of this gene detected under TPS is further evidence for TR demand in the amastigote stage.

#### Transport

Two different genes coding for 3'NT/Nase are located in tandem in chromosome 31 and are down-regulated under TPS and TS, as well as in intracellular amastigotes according to unpublished custom data and previous analyses (reviewed in [13]). CLUSTAL alignments show exact identity in the central region, and differences between N-ter and C-ter domains. 3'-NT/Nase is essential for *Leishmania *parasites because they are not able to synthesise purines *de novo*. It localizes to the plasma membrane, may play a role in purine acquisition and its substrates are 3'-ribonucleotides (3'AMP and 3'-IMP) and nucleic acids [42,44]. According to previous observations [45], these genes are up-regulated in promastigotes in the logarithmic phase of axenic culture and absence of expression is shown in the amastigote stage. Interestingly, we have found down-regulation of these genes under TPS and TS with respect to CC. Consequently, temperature down-regulates 3'-NT/Nase expression.

Apart from the GT genes already mentioned, aquaporin (AQ) and Zn transporter (ZnT) genes are down-regulated under TPS. The AQ gene is related to changes in cell volume and shape during the life cycle of the parasite and it also acts both as an osmotic sensor and in passive transport of solutes. It may be related to the osmotactic response shown by the promatigote stage inside the insect vector for migration to the foregut [46]. In addition, four different aminoacid permeases are differentially regulated: two are up-regulated (LinJ31_V3.0590 and LinJ31_V3.1580) and two down-regulated (LinJ27_V3.0530 and LinJ31_V3.0610). Furthermore, three different pteridine transporter genes are differentially regulated by the effect of temperature and pH: PT3 LinJ10_V3.0410 and PT LinJ14_V3.1440 are up-regulated under TPS, while PT LinJ06_V3.1320 is down-regulated, and this pattern is repeated under TS except for PT3. Trypanosomatids are auxotrophes for pteridines and therefore they depend on exogenous sources of these compounds. Finally, the vacuolar type proton-translocating pyrophosphatase gene (vH^+^-PPi) is down-regulated under TPS.

#### Signal transduction and cell cycle regulation

Protein kinase and cAMP signalling pathways have not been elucidated in *Leishmania *to date. A first approach is to find the TPS-generated down-regulation of a receptor-type adenylate cyclase-like (ACR), a serine/threonine protein phosphatase (Ser/Thr PPase) and three non-ligated cysteine peptidase/calpain C2 family (C2cp) genes. C2cp genes are also involved in cytoskeleton remodelling (see further explanation in the following section). The down-regulation under TPS of a serine/threonine protein kinase (Ser/Thr PK) may be related to the down-regulation of calmodulin, which binds calcium divalent cations after its activation by PK phosphorylation. Ser/Thr PK is also down-regulated under TS. The exact physiological functions of the calcium-calmodulin pathway have not been described in *Leishmania *either and the inositolphospholipid regulator pathway also remains uncharacterised. For this reason, there is no known biological meaning for the down-regulation of PI3K and the up-regulation of INO1 under TPS and TS found in the microarray hybridisation analysis. INO1 down-regulation has been observed in *L. infantum *axenic and intracellular amastigotes [28]. Apart from this, a cyclin dependent kinase binding protein (Cdkbp) and a serine peptidase E from the S51 family (S51) are also down-regulated and might be involved in S-phase or mitosis entry.

#### Cytoskeleton and flagellum

Several genes associated with the flagellar and paraflagellar rod structures are down-regulated under TPS: coronin, dynein heavy chain LinJ36_V3.2010 and LinJ26_V3.1000 and PFR1D, the latter also being up-regulated under TS. Dynein heavy chain LinJ26_V3.1000 down-regulation has also been described in AL [28] and intracellular amastigotes (unpublished data). Apart from that, we have found that an unknown tubulin-associated GTPase is up-regulated under TPS, as well as RABreg, an activator of prenylated RAB GTPases. An analogue of a RAB small GTPase is up-regulated in *L. major *amastigotes and may be related with pathogeny, as vesicle transport is essential for extracellular nutrient acquisition, release of virulence factors, microbicidal resistance and evasion of host immune responses [47]. In addition, calpains are involved in cytoskelleton remodelling and signal transduction in kinetoplastid parasites (reviewed in [48]). μ-calpain C2cp LinJ20_V3.1230 (see also the *Signal transduction and cell cycle regulation *subsection) is down-regulated under TPS and up-regulated in metacyclic promastigotes [25]. Moreover, *L. mexicana *[17] and *L. donovani *[33] mature intracellular amastigotes also down-regulate this gene. As a consequence, the greatest transcript levels of -calpain are reached in metacyclic promastigotes.

#### gRNAs

According to BLAST outcome mapping against GenBank database, 19 clones map against 4 different minicircle sequences (contig 200, 692, 878 and 957) (Additional file 3: Table S7) and according to the microarray hybridisation analysis, they must contain an up-regulated gene under TPS. Provided that each minicircle contains only a single gRNA gene for site-specific uridine insertion/deletion type RNA editing, 4 gRNA genes with unknown target are presumably up-regulated under TPS. The gRNA genes corresponding to contigs 878 (1 clone) and 957 (16 clones) have also been found to be up-regulated under PS (Additional file 3: Table S8) and were previously found as up-regulated in metacyclic promastigotes [25].

#### Other genes

*Leishmania *promastigotes use chitinase to break the chitinous peritrophic membrane inside the gut of the sand-fly vector [49]. Chitinase gene is up-regulated in TPS-induced AL, which is consistent with chitinase overexpression reported in amastigotes, as well as the associated enhanced lesion development observed in mice [50], suggesting an additional or different function for this gene. The microarray hybridisation analysis has also revealed that SbGRP is down-regulated in TPS-induced AL forms, as well as in *L. mexicana *[17], *L. major *[32] and *L. infantum *(unpublished data) intracellular amastigotes. In fact, a decrease in pentavalent antimonial resistance is a feature of AL, together with round morphology typical of amastigotes, the up-regulation of A2 cluster and the down-regulation of 3'NT/Nase, as reviewed previously [13].

The membrane bound acid phosphatase gene (MBAP) is down-regulated under TPS as revealed by microarray analysis. There is evidence to confirm that it is essential for cell survival, because it plays a critical role in nutrition. It is located in small vesicles between the Golgi apparatus and the flagellar pocket (secretory pathway) [51]. MBAP levels have been described as being higher in procyclic promastigotes rather than in metacyclics. It was reported that its activity is higher in virulent clones and consequently, it was supposed that it was involved in virulence in spite of the higher levels of MBAP protein found in logarithmic phase promastigotes according to [52], but further experiments have demonstrated the opposite. *L. mexicana *MBAP knockout parasites show that it is neither involved in the infection process nor required for amastigote survival in the infected host cell [51]. This supports our results concerning MBAP gene down-regulation in TPS-induced AL forms.

Hybridisation analysis has revealed the down-regulation of a hypothetical protein (CoB) with copper ion binding/transport and chaperone GO molecular functions under TPS. CoB is located in the mitochondrial lumen according to GO cellular component term analysis. Two genes from the HASP/SHERP cluster are differentially regulated by TPS: a small hydrophilic ER-associated protein (SHERP) is down-regulated by TPS; and hydrophilic surface protein (HASPB) is up-regulated under TPS, as well as in intracellular *L. donovani *promastigotes at the post-translational level [33]. In contrast, down-regulation was reported in *L. major *[16]. Despite these genes being previously presumed to be metacyclic promastigote-specific in *L. major *[16], we have reported a different pattern for HASPB in *L. infantum*. Finally, this analysis also revealed the up-regulation of an esterase-like protein, carbamoyl-phosphate synthetase (CPS), a short chain dehydrogenase and ubiquitin-conjugating enzyme-like proteins in TPS-induced AL, and the latter also under TS.

## Conclusions

Absence of gp46 expression observed by means of IFA and up-regulation of the amastigote-specific A2 gene has been found in TPS-treated cells. As a consequence, we know that TPS leads to differentiation into AL. The up-regulation of several amastin genes and the down-regulation of 3'NT/Nase and SbGRP genes under TPS and TS point to a developmental process towards amastigote differentiation by the combined effect of temperature increase and acidification and the single effect of temperature. By contrast, none of these genes have been found to be differentially regulated under PS, which suggests that pH decrease itself does not prompt amastigote differentiation in the parasite. A wider analysis of TPS-, TS- and PS-induced expression profiles throughout HCL-ST clustering analysis of gene expression supports temperature shift alone or combined with acidification as triggering differentiation towards the amastigote stage whereas acidification itself does not. In fact, we have described examples of known annotated genes taken directly from the microarray output, namely the up-regulation of RRS1, INO1 and aATP11 and the down-regulation of 3'NT/Nase, PT, GT, SOD, PI3K, FKBP, calmodulin and lathosterol oxidase. These observations have led us to conclude that temperature increase is more relevant than pH decrease in the differentiation process to the amastigote stage with regard to transcriptome variation in *L. infantum*. In addition, we have provided the first description of transcriptome variation induced by the specific influence of temperature increase and acidification.

## Methods

### Parasite cultures and RNA isolation

Cultures of *L. infantum *isolate M/CAN/ES/98/10445 (zymodeme MON-1) from early passages after axenization were grown at a starting density of 4 × 10^6 ^mid-logarithmic phase promastigotes/ml in RPMI 1640 medium supplemented with L-glutamine (Cambrex, Karlskoga, Sweden), 10% heat inactivated foetal bovine serum (HIFBS) (Cambrex) and 100 μg/ml streptomycin - 100 IU/ml penicillin (Cambrex) at 27°C/pH7.2 (CC), 37°C/pH4.5 (TPS), 37°C/pH7.2 (TS) or 27°C/pH4.5 (PS). Cell density was counted daily and promastigotes were harvested at 2000 g for 10 min on day 4. RNA isolations were performed from 2 × 10^8 ^cells/ml of TRIzol^® ^reagent (Invitrogen, La Jolla, CA) following the manufacturer's instructions. Three biological replicates of the cultures were carried out for each of the conditions described.

### gp46 IFA

Cells were fixed with acetone:methanol (1:1) at -20°C for 10 min at a density of 2 × 10^6^/5 μl drop. Then, they were incubated with purified anti-gp46 monoclonal IgG antibody at 37°C for 30 min in a hydration chamber, washed three times with PBS by mild agitation for 10 min, incubated with fluorescein isotiocyanate (FITC)-conjugated goat anti-mouse IgG antibody (Serotec, Raleigh, NC) and 0.1% Evans' Blue (Fisher, Pittsburgh, PA). Washes were repeated and preparations mounted with 90% glycerol. Negative control of the primary antibody was anti-rabbit complement factor H monoclonal IgG and the first incubation was carried out with PBS for the negative control of the secondary antibody. SIM 110 monoclonal IgG antibody against soluble leishmanial antigens (SLA) was used as a positive control. Anti-gp46, anti-rabbit factor H and SIM110 antibodies were kindly provided by Mercedes Domínguez (Centro Nacional de Microbiología, Virología e Inmunología Sanitarias, Instituto de Salud Carlos III, Majadahonda, Spain).

### *L. infantum *DNA microarray construction and hybridisation

*L. infantum *DNA microarray construction and hybridisation assays were carried out as described previously [25]. To summarize, microarrays were generated by long template PCR amplification from a complete shotgun DNA-pUC18 genomic library with m13-pUC18 primers and spotting onto epoxy-coated slides. RNA quality was assessed by capillary electrophoresis, mRNA was amplified, cDNA was synthesised and indirectly labelled and *L. infantum *DNA microarrays blocked, hybridised and washed as detailed in [25]. Hybridisation assays were performed as follows: 37°C/pH4.5 vs. 27°C/pH7.2 (TPS vs. CC), 37°C/pH7.2 vs. 27°C/pH7.2 (TS vs. CC) and 27°C/pH4.5 vs. 27°C/pH7.2 (PS vs. CC). Hybridised microarrays were scanned and fluorescence intensity was analysed for Cy3 (532 nm) and Cy5 (635 nm) with local feature background subtraction (GenePix 4100A scanner and software, Axon Instruments, Foster City, CA). LOWESS per pin algorithm was used to normalise raw data (AlmaZen software, BioAlma, Tres Cantos, Spain). After that, comparative analysis of the replicates by paired t-test and selection of spots with meaningful values of stage-specific regulation were performed as described [25].

### DNA sequencing and analysis

Clones corresponding to selected spots were sequenced and mapped following a strategy that has already been described in detail [25]. Briefly, insert ends were dideoxi-sequenced with m13-pUC18 primers and aligned against the *L. infantum *genome project sequence in General Feature Format (GFF) deposited in a GBrowse database. Forward and reverse reads were mapped to define the boundaries of the clones in the genome of *L. infantum*. Depending upon the insert length, the success of sequencing reactions of both ends and genome sequence complexity, three possibilities arose: when one pair of convergently oriented alignments separated by up to 11 Kbp were found, the clone mapping outcome was defined as type *a*; when more than one pair of alignments fulfilled those conditions, the best pair of alignments was used to define the boundaries of the clone, resulting in a type *b *outcome; and when those requirements were not fulfilled (incongruent pair of alignments or unpaired alignments), the outcome was defined as type *c*. Some of the clones were annotated by a custom Glimmer 3.0 analysis because they did not map against genes previously annotated on the *L. infantum *genome project sequence. Stage-specifically regulated genes were re-annotated and analysed with BLAST2GO to establish molecular function and biological process GO term distribution among them based on α-scores [53].

Multi-experiment SAM and the subsequent iterative hierarchical clustering-support tree analysis (HCL-ST) were carried out with TIGR's MultiExperiment Viewer 4.3 (MEV) by introducing normalised microarray hybridisation data matrixes (including medians and standard deviations of intensity and F values) of clones with significant differential regulation in each individual experiment. SAM p-value cutoff was 0.05, the same as for the previous independent t-tests for each experiment. HCL-ST was performed independently for significant and non-significant genes. ST algorithm with a jackknifing resampling option and 100 iterations for the construction and clustering of the gene expression matrix were applied in HCL-ST analysis. CLUSTALW2 was used for sequence alignments of amastin, GT and 3'NT/Nase genes differentially regulated by the effect of pH and/or temperature and CBS's TMHMM 2.0 for the prediction of transmembrane helices in these proteins.

### qRT-PCR

qRT-PCR reactions were performed to determine whether a gene overlapping with a type c sequence end is developmentally regulated or to ascertain which gene is developmentally regulated in the clones overlapping more than one gene. We described previously the procedure applied [25]. The reference gene was 18S rRNA. When there were two copies of a gene in tandem in a given clone but one of the copies lacked a segment of the 5' end or differed in a specific sequence (GT and 3'NT/Nase genes), two pairs of primers were designed for qRT-PCR. A complete list of primers used for qRT-PCR is provided in Additional file 5.

## Abbreviations

aap: amino acid permease; aATP11: amino acid transporter 11; ABCE: ABC transporter subfamily E (ribonuclease L-inhibitor) gene; ACR: receptor-type adenylate cyclase; ACT: acyl-CoA transferase; AL: amastigote-like; ALD: fructose-1,6-bisphosphate aldolase; AQ: aquaporin; CC: culture control conditions; C2cp: cysteine protease family C2; cdkbp: cyclin-dependent kinase binding protein; C(III)O: coproporphyrinogen oxidase; CoA: coenzyme A; CoB: cofactor binding protein; Cph: cyclophilin; CPS: carbamoyl phosphate synthetase; Cy: cyanin; cyt b5: cytochrome b5; DAG: direct acyclic graph; 1,2-DAG, 1,2-diacylglycerol; DGC: directional gene cluster; DHAP: dihydroxyacetone phosphate; EF1α, elongation factor 1 α; eIF5a: eukaryotic translation initiation factor 5a; ER: endoplasmic reticulum; ETCH: electron transport chain; F: Fold change; FA: fatty acid; FITC: fluorescein isotiocyanate; FKBP: FK506-binding protein; FU: Fluorescence Units; GAPDH: glyceraldehyde-3-phosphate dehydrogenase; αGII-HPB: hypothetical calcium ion binding protein from α-glycosidase II complex; gMDH: glycosomal malate dehydrogenase; GNAT: glucose-6-phosphate N-acetyltransferase; GO: Gene Ontology; gp46: 46 KDa surface glycoprotein; GPDE: glycerolphosphodiester phosphodiesterase; gRNA: guide RNA; GT: glucose transporter; H3: histone H3; HASPB: hydrophilic surface protein B; HCL-ST: Hierarchical clustering-Support Tree; HIFBS: heat inactivated foetal bovine serum; HMG-CoA: 3-hydroxymethylglutaryl-CoA; HMGCR: HMG-CoA reductase; HPT: hypothetical protein transport protein; HTreg: hypothetical transcription regulator; IFA: immunofluorescence analysis; IPC: inositol phosphoceramide; INO1: *myo*-inositol-1-phosphate synthase; LOWESS: Locally Weighted Scatter Plot Smoothing algorithm; LPG: lypophosphoglycan; mACDH: mitochondrial acyl-CoA dehydrogenase; MBAP: membrane bound acid phosphatase; MEV: Multi Experiment Viewer; MGL: monoglyceride lipase; *mmc*: minichromosome maintenance complex protein; MRP: multidrug resistance protein; NPC: nuclear pore complex; 3'NT/Nase: 3'-nucleotidase/nuclease; *β*-ox: *β*-oxidation of FA; PES: prostaglandin peroxide synthase complex; PFR1D: paraflagellar rod protein 1D; PG: phosphoglycerate; PGFS: prostaglandin F synthase; PGM^BPI^: bisphosphoglycerate-independent phosphoglycerate mutase; PI3K: phosphatidylinositol triphosphate kinase; PO: protoporphyrinogen oxidase; PPG: proteophosphoglycan; PS: pH shift; PSA2: promastigote-specific surface antigen 2; PT: pteridine transporter; qRT-PCR: relative quantitative real time PCR; R: set of ribosomal proteins; RABreg: Rab GTPase regulator; RNAbp: RNA-binding protein; RRS1: ribosome assembly protein; S51: serine peptidase A family S51; SAGE: Serial Analysis of Gene Expression; SAM: Serial Analysis of Microarrays; Ser/Thr PK: serine/threonine protein kinase; SbGRP: sodium stibogluconate resistance protein; Ser/Thr PPase: Ser/Thr protein phosphatase; SHERP: small hydrophilic ER-associated protein; SLA: soluble leishmanial antigen; SOD: superoxide dismutase; TAG: triacylglycerol; TCA: tricarboxylic acid cycle; TFSUI1: translation factor SUI1; TGL: TAG lipase; TPS: temperature-pH shift; TS: temperature shift; TR: trypanothione reductase; vH^+^-PPi: vacuolar-type proton-translocating pyrophosphatase; ZnT: Zn transporter.

## Authors' contributions

PJA, AA, ASG, MM, VP and VL contributed to the design of shotgun genomic DNA microarrays. PJA and ASG constructed the genomic library and PJA, AA, ASG and MM contributed to the microarray construction. PJA, AA, MM and EG contributed to the optimisation of the hybridisation procedure. The experimental design of biological sample preparation and expression profile analysis of temperature and acidification was carried out by PJA, AT and VL. PJA and AT contributed to gp46 IFA. Microarray hybridisation data acquisition and analysis was performed by PJA and AA with the assistance of MM, EG and VP. Sequence analysis, clone assembly and mapping against *L. infantum *genome, gene annotations with *Glimmer *software, minicircle sequence analyses, GO annotations and BLAST2GO analysis were performed by MJG. qRT-PCR analyses were performed by PJA with the assistance of AT. PJA, AA and VL contributed to the interpretation of data. The manuscript was drafted by PJA, AA and VL. All authors revised the manuscript thoroughly and made important contributions to the intellectual content of this manuscript. VL approved the version to be published.

## Supplementary Material

Additional file 1 **Electropherograms of total RNA samples**. Figure S1. 18S, 23Sα and 23Sβ spikes, absence of DNA contamination and RNA degradation. Click here for file

Additional file 2 **Scatter plots of normalised and contrasted microarray hybridisation data**. Figure S2. Spots that fulfill criteria to be considered as differentially regulated are highlighted. Click here for file

Additional file 3 **Differentially regulated hypothetical and unknown genes and unresolved clones**. Tables S1, S2, S3, S4, S5, S6, S7 and S8. Tables S7 and S8 describe clones containing minicircle sequences. Click here for file

Additional file 4 **DAGs (BLAST2GO output)**. Figures. S3 and S4. GO codes for functions directly annotated on differentially regulated genes found in this study. Each GO code is associated to a custom code to find annotations on genes in Tables 1, 2, 3, 4 and 5. Click here for file

Additional file 5 **3'NT/Nase and GT alignments and oligonucleotides for qRT-PCR**. Figure S6 (GT and 3'NT/Nase alignments) and Additional file 3: Table S9 (oligonucleotides for qRT-PCR). Click here for file

Additional file 6 **Fold change clusters of differentially regulated genes including clone names**. Figure S5. Supplementary information for Figure 4 identifying the profiles with the clone numbers. Click here for file
