# Supplementary material for: Temperature increase prevails over acidification in gene expression modulation of amastigote differentiation in Leishmania infantum
Source: BMC Genomics. 2010 Jan 14;11:31. doi: 10.1186/1471-2164-11-31 (PMC2845110; doi:10.1186/1471-2164-11-31)

ADDITIONAL FILE 1

Figure S1. Electropherograms of total RNA samples obtained after TPS, TS and PS treatments. Fluorescence units (FU) are given on y axis and time in seconds on abscissa. The first spike corresponds to RNA 6000 Nano Marker (Agilent Technologies) and 18S and 23S (α, β) spikes to ribosomal RNAs. (A) 27 ºC/pH7.2 (CC). (B) 37 ºC/pH7.2 (TS). (C) 27 ºC/pH4.5 (PS). (D) 37 ºC/pH4.5 (TPS).

A. 27 ºC/pH7.2

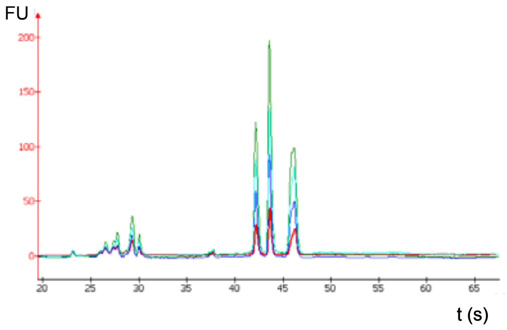

B. 37 ºC/pH7.2

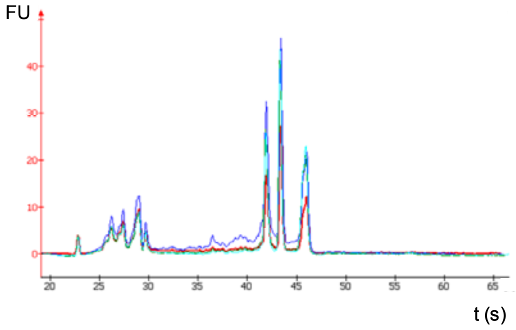

C. 27 ºC/pH4.5

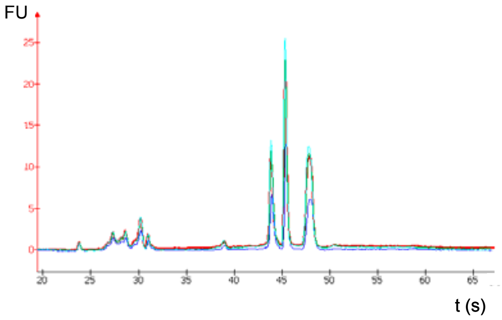

D. 37 ºC/pH4.5

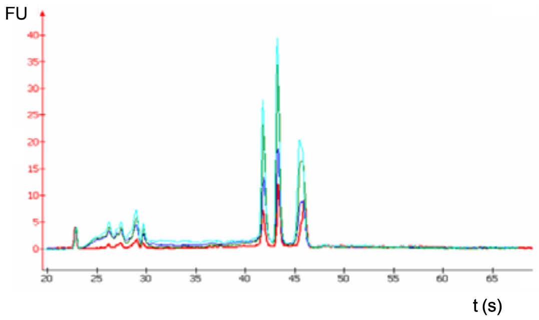

Supplement: Additional file 1 — Electropherograms of total RNA samples. Figure S1. 18S, 23Sα and 23Sβ spikes, absence of DNA contamination and RNA degradation. [file 1471-2164-11-31-S1.PDF]
