# Supplementary material for: Temperature increase prevails over acidification in gene expression modulation of amastigote differentiation in Leishmania infantum
Source: BMC Genomics. 2010 Jan 14;11:31. doi: 10.1186/1471-2164-11-31 (PMC2845110; doi:10.1186/1471-2164-11-31)

ADDITIONAL FILE 2

Figure S2. Average M/A scatter plots of three replicate microarray hybridisation analyses for each of the conditions assayed (TPS, TS and PS). M=($log_2Ri-log_2Gi$) and A=[($log_2Ri+log_2Gi$)/2], where R and G are respectively red (Cy5) and green (Cy3) intensity values. Red spots correspond to selected DNA fragments containing a gene up-regulated at least 1.7 times and green spots represent those down-regulated at least 1.7 times in each condition referred to control promastigotes (CC). Further criteria for spot selection are detailed in Results and Discussion section.

A. TPS vs. CC

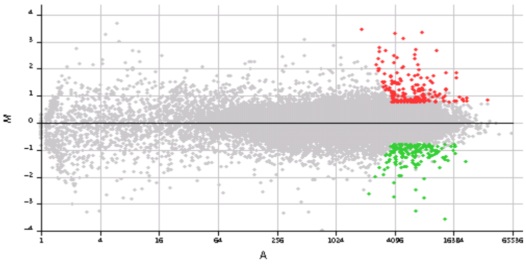

B. TS vs. CC

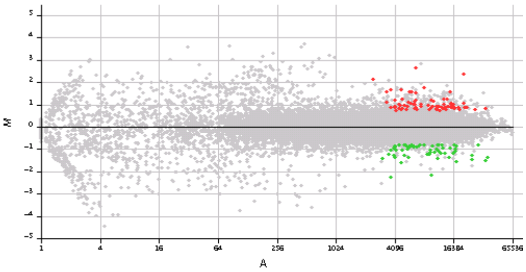

C. PS vs. CC

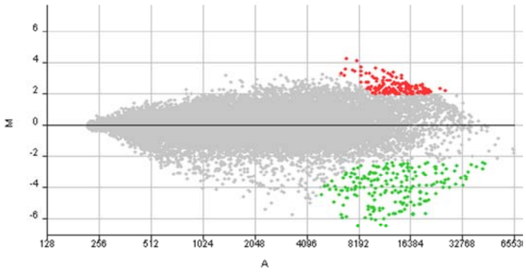

Supplement: Additional file 2 — Scatter plots of normalised and contrasted microarray hybridisation data. Figure S2. Spots that fulfill criteria to be considered as differentially regulated are highlighted. [file 1471-2164-11-31-S2.PDF]
