# Supplementary material for: Temperature increase prevails over acidification in gene expression modulation of amastigote differentiation in Leishmania infantum
Source: BMC Genomics. 2010 Jan 14;11:31. doi: 10.1186/1471-2164-11-31 (PMC2845110; doi:10.1186/1471-2164-11-31)
Supplement: Additional file 3 — Differentially regulated hypothetical and unknown genes and unresolved clones. Tables S1, S2, S3, S4, S5, S6, S7 and S8. Tables S7 and S8 describe clones containing minicircle sequences. [file 1471-2164-11-31-S3.PDF]

ADDITIONAL FILE 3.

Table S1. Genes coding for hypothetical and unknown proteins that are differentially regulated under TPS. F < -1.7 indicates gene down-regulation and F > 1.7 up-regulation.

Clone	*F*	*$Log_2F \pm SD$*	*p*	*GenBank*	*e-value*		*Def.*	*Id.*	*Annotated Gene Function*	*qRT-PCR*	
					Fw	*Rv*				+/-	F ± SD
Lin21E6	2.91	1.5 ± 0.0	0.000	GS599047	0	0	b	LinJ20_V3.1060	Hypothetical protein, unknown function	N.D.	
**Lin44H5**	1.71	0.8 ± 0.2	0.023	GS599048	0	0	b	LinJ31_V3.0090	Hypothetical protein, conserved	N.D.	
								LinJ31_V3.0100	Hypothetical protein, conserved	N.D.	
Lin45D9	1.83	0.9 ± 0.2	0.020	GS599049	0	0	b	LinJ20_V3.0430	Hypothetical protein, conserved	N.D.	
Lin56E9	2.74	1.5 ± 0.1	0.001	GS599050	3e-52	2e-120	b	LinJ06_V3.0830	Hypothetical protein, conserved	N.D.	
Lin59C10	1.80	0.8 ± 0.20	0.018	GS599051	0	1e-180	b	LinJ36_V3.2950	Hypothetical protein, conserved	N.D.	
								LinJ36_V3.2960	Hypothetical protein, conserved	N.D.	
Lin66F8	1.92	0.9 ± 0.2	0.017	GS598865	0	3e-132	a	LinJ33_V3.2470	Succinyl-CoA:3-ketoacid-CoA transferase, mitochondrial precursor, putative (3-oxoacid-CoA transferase)	-	1.4 ± 0.2
								LinJ33_V3.2480	Hypothetical protein, conserved	N.D.	
Lin71E8	1.82	0.9 ± 0.3	0.032	GS599052	0	0	b	LinJ26_V3.1780	Hypothetical protein	N.D.	
Lin76G6	1.97	1.0 ± 0.1	0.005	GS599053	0	0	b	LinJ31_V3.2730	Hypothetical protein, unknown function	N.D.	
Lin77G12	1.82	0.9 ± 0.3	0.042	GS599054	0	0	b	LinJ32_V3.1720	Hypothetical protein, conserved	N.D.	
Lin99C10	1.99	1.0 ± 0.3	0.023	GS599055	0	0	b	LinJ02_V3.0470	Hypothetical protein, conserved	N.D.	
Lin103F2	3.30	1.7 ± 0.2	0.004	GS599056	0	0	b	LinJ32_V3.2450	Hypothetical protein, unknown function	N.D.	
Lin106B1	1.77	0.8 ± 0.2	0.015	GS599057	0	0	b	LinJ36_V3.5150	Hypothetical protein, conserved	N.D.	
Lin134A3	2.40	1.3 ± 0.3	0.013	GS599059	0	0	b	LinJ04_V3.0630	Hypothetical protein, conserved	N.D.	
								LinJ04_V3.0640	Hypothetical protein	N.D.	
**Lin135E9**	1.77	0.8 ± 0.3	0.042	GS599060	0	0	b	**LinJ34_V3.0060**	**Hypothetical protein, conserved**	N.D.	
								LinJ34_V3.0070	Ascorbate-dependent peroxidase, putative	-	-1.2 ± 0.3
Lin151D9	1.76	0.8 ± 0.2	0.011	GS599061	0	1e-102	b	LinJ30_V3.3210	Hypothetical protein, conserved	N.D.	
								LinJ30_V3.3220	Hypothetical protein, conserved	N.D.	
								LinJ30_V3.3230	Hypothetical protein, conserved	N.D.	
Lin154G1	1.80	0.9 ± 0.2	0.013	GS599062	0	3e-120	b	LinJ30_V3.3210	Hypothetical protein, conserved	N.D.	
								LinJ30_V3.3220	Hypothetical protein, conserved	N.D.	
								LinJ30_V3.3230	Hypothetical protein, conserved	N.D.	
Lin156B2	1.82	0.9 ± 0.2	0.025	GS598883	0	0	b	LinJ33_V3.2960	Hypothetical protein, conserved	N.D.	
Lin181E2	1.99	1.0 ± 0.3	0.035	GS599063	0	0	c	LinJ22_V3.1460	Hypothetical protein, conserved	N.D.	
Lin183A3	1.75	0.8 ± 0.1	0.010	GS598886	0	0	b	LinJ24_V3.2250	Hypothetical protein, conserved	N.D.	
Lin200D12	1.80	0.8 ± 0.2	0.021	GS599064	0	0	b	LinJ25_V3.0650	Hypothetical protein, conserved	N.D.	
								LinJ25_V3.0660	Hypothetical protein, conserved	N.D.	
Lin220H6	1.85	0.9 ± 0.1	0.007	GS599065	0	0	a	LinJ31_V3.2340	Hypothetical protein, conserved	N.D.	
Lin223F2	1.73	0.8 ± 0.3	0.044	GS598894	0	0	b	LinJ13_V3.0330	Unknown	N.D.	
Lin232F6	1.83	0.9 ± 0.1	0.006	GS599066	0	0	b	LinJ17_V3.0020	Hypothetical protein, conserved	N.D.	
								LinJ17_V3.0030	Hypothetical protein, conserved	N.D.	
								LinJ17_V3.0040	Hypothetical protein, conserved	N.D.	
Lin254B10	2.24	1.2 ± 0.5	0.045	GS599067	0	0	b	LinJ19_V3.1200	Hypothetical protein, conserved	N.D.	
								LinJ19_V3.1210	Hypothetical protein, conserved	N.D.	
Lin6A1	-1.74	-0.8 ± 0.2	0.015	GS599068	0	2e-130	b	LinJ18_V3.0810	Hypothetical protein, conserved	N.D.	
Lin3H4	-1.98	-1.0 ± 0.0	0.001	GS599069	5e-72	1e-125	b	LinJ23_V3.0420	Hypothetical protein, conserved	N.D.	
Lin11F2	-2.07	-1.1 ± 0.2	0.013	GS599070	0	0	b	LinJ25_V3.2090	Hypothetical protein, conserved	N.D.	
Lin13F5	-1.77	-0.8 ± 0.3	0.031	GS599071	0	0	b	LinJ09_V3.1540	Hypothetical protein, conserved	N.D.	
								LinJ09_V3.1550	Hypothetical protein, conserved	N.D.	
								LinJ09_V3.1560	Hypothetical protein, conserved	N.D.	
Lin15G8	-1.92	-0.9 ± 0.3	0.024	GS599072	4e-76	3e-86	b	LinJ23_V3.0420	Hypothetical protein, conserved	N.D.	
Lin16F5	-1.84	-0.9 ± 0.2	0.011	GS599073	1e-180	0	b	LinJ24_V3.0590	Hypothetical predicted transmembrane protein	N.D.	
Lin31E6	-1.72	-0.8 ± 0.3	0.035	GS599074	0	3e-120	b	LinJ25_V3.0790	Hypothetical protein, conserved	N.D.	
**Lin58A11**	-2.00	-1.0 ± 0.4	0.043	GS599075	8e-176	0	b	**LinJ35_V3.3190**	**Hypothetical protein, conserved**	N.D.	
Lin60E5	-1.80	-0.8 ± 0.3	0.035	GS598921	0	0	b	LinJ26_V3.0970	Hypothetical protein, conserved	N.D.	
Lin61D3	-1.92	-0.9 ± 0.1	0.005	GS599076	0	0	b	LinJ31_V3.0330	Hypothetical protein, conserved	N.D.	
Lin70D3	-2.07	-1.0 ± 0.4	0.042	GS599077	0	0	a	LinJ31_V3.1210	Hypothetical protein, unknown function	N.D.	
**Lin74F6**	-1.88	-0.9 ± 0.1	0.005	GS599078	0	0	a	LinJ29_V3.1820	Hypothetical protein, conserved	N.D.	
								LinJ29_V3.1830	Hypothetical protein, conserved	N.D.	
Lin78F3	-3.15	-1.7 ± 0.1	0.002	GS599079	0	0	b	LinJ26_V3.0970	Hypothetical protein, conserved	N.D.	
								LinJ26_V3.0980	Hypothetical protein, conserved	N.D.	
Lin97F6	-1.80	-0.8 ± 0.3	0.029	GS598932	0	0	b	LinJ26_V3.0460	Hypothetical protein, conserved	N.D.	
Lin105B4	-2.39	-1.3 ± 0.2	0.009	GS599080	0	0	b	LinJ14_V3.1110	Unknown	N.D.	
								LinJ14_V3.1120	Hypothetical protein, conserved	N.D.	
Lin105B8	-5.59	-2.5 ± 0.4	0.010	GS599081	0	0	b	LinJ14_V3.1110	Unknown	N.D.	
								LinJ14_V3.1120	Hypothetical protein, conserved	N.D.	
Lin112D11	-1.94	-1.0 ± 0.2	0.021	GS599082	1e-54	0	b	LinJ31_V3.2430	Hypothetical protein, conserved	N.D.	
Lin122H8	-1.74	-0.8 ± 0.3	0.036	GS599083	0	0	b	LinJ32_V3.1600	Hypothetical protein, conserved	N.D.	
								LinJ32_V3.1610	Hypothetical protein, conserved	N.D.	
								LinJ32_V3.1620	Hypothetical protein, unknown function	N.D.	
Lin124H4	-2.89	-1.5 ± 0.5	0.001	GS599084	0	0	b	LinJ23_V3.0700	Hypothetical protein, conserved	N.D.	
Lin128A3	-1.71	-0.8 ± 0.2	0.013	GS599085	0	0	b	LinJ08_V3.0430	Hypothetical protein, conserved	N.D.	
Lin148G1	-1.70	-0.8 ± 0.1	0.002	GS599086	0	0	b	LinJ30_V3.2310	Hypothetical protein, conserved	N.D.	
								LinJ30_V3.2320	Hypothetical protein, conserved	N.D.	
								LinJ30_V3.2330	Hypothetical protein, conserved	N.D.	
**Lin155G12**	-2.81	-1.5 ± 0.2	0.004	GS599087	7e-133	2e-19	a	LinJ35_V3.3770	Hypothetical protein, conserved	N.D.	
								LinJ35_V3.3780	Hypothetical protein, conserved	N.D.	
Lin158A10	-2.40	-1.3 ± 0.2	0.008	GS598950	0	0	b	LinJ23_V3.0870	Hypothetical protein, conserved	N.D.	
Lin167B9	-2.70	-1.4 ± 0.5	0.040	GS599088	1e-60	1e-14	a	LinJ30_V3.2310	Hypothetical protein, conserved	N.D.	
								LinJ30_V·.2320	Hypothetical protein, conserved	N.D.	
								LinJ30_V3.2330	Hypothetical protein, conserved	N.D.	
**Lin169B8**	-2.79	-1.5 ± 0.2	0.009	GS599089	0	0	b	LinJ24_V3.2320	Hypothetical predicted multi-pass transmembrane protein	N.D.	
								LinJ24_V3.2330	Hypothetical protein, conserved	N.D.	
Lin186A8	-2.63	-1.4 ± 0.1	0.003	GS599090	0	0	b	LinJ25_V3.2090	Hypothetical protein, conserved	N.D.	
Lin193H8	-2.71	-1.4 ± 0.4	0.024	GS599091	0	0	b	LinJ06_V3.1350	Hypothetical protein, unknown function	N.D.	
Lin194B7	-1.80	-0.8 ± 0.2	0.016	GS599092	0	0	b	LinJ23_V3.1830	Hypothetical protein, unknown function	N.D.	
Lin202H7	-2.10	-1.1 ± 0.2	0.013	GS599093	1e-97	5e-106	a	LinJ32_V3.2130	Hypothetical protein, conserved	N.D.	

**Lin209B12**	-2.07	-1.1 ± 0.2	0.013	GS599094	0	0	a	LinJ35_V3.4170	Hypothetical protein, conserved	N.D.	
								LinJ35_V3.4180	Hypothetical protein, conserved	N.D.	
								LinJ35_V3.4190	Hypothetical protein, conserved	N.D.	
Lin230F8	-3.72	-1.9 ± 0.7	0.038	GS599095	0	0	b	LinJ06_V3.1350	Hypothetical protein, unknown function	N.D.	
Lin243B8	-1.80	-0.8 ± 0.1	0.010	GS599096	0	0	b	LinJ06_V3.1350	Hypothetical protein, unknown function	N.D.	
Lin244F12	-2.16	-1.1 ± 0.3	0.021	GS599097	0	0	b	LinJ17_V3.0970	Hypothetical protein, conserved	N.D.	
Lin273D8	-2.16	-1.1 ± 0.2	0.013	GS599098	0	0	b	LinJ33_V3.1070	Hypothetical protein, conserved	N.D.	
								LinJ33_V3.1080	Hypothetical protein, conserved	N.D.	
Lin280B2	-1.81	-0.9 ± 0.2	0.018	GS599099	0	0	b	LinJ32_V3.0360	Hypothetical protein, conserved	N.D.	
								LinJ32_V3.0370	Hypothetical protein, conserved	N.D.	
Lin294B2	-1.73	-0.8 ± 0.2	0.024	GS599058	0	0	b	LinJ29_V3.2200	Hypothetical protein, conserved	N.D.	

Table S2. Unresolved clones for TPS. These clones fulfil spot selection requirements (see Materials and methods section in the article) but correspond to minicircle sequences. They do not map against already annotated genes in the *L. infantum* genome project sequence (custom Glimmer annotations are indicated whenever predicted) or clone sequence assembly outcome is *c* and no qRT-PCR assay has been performed to find out which gene(s) are differentially regulated. F < -1.7 indicates gene down-regulation and F > 1.7 up-regulation.

Clone	*F*	*Log_2F ± SD*	*p*	*GenBank*	*e-value*		*Def.*	*Id.*	*Annotated Gene Function*	*qRT-PCR*	
					Fw	*Rv*				+/-	F ± SD
Lin8C12	2.30	1.2 ± 0.4	0.044	GS599100	0	-	c			N.D.	
Lin10E6	1.76	0.8 ± 0.2	0.018	GS599101	3e-101	-	c			N.D.	
Lin13C3	1.78	0.8± 0.0	0.000	GS599102	0	-	c	LinJ18_V3.1050	5-oxoprolinase, putative	N.D.	
Lin19B1	2.56	1.4 ± 0.4	0.026	GS599103	0	0	c	LinJ02_V3.0580	Hypothetical protein, conserved	N.D.	
Lin49H10	1.71	0.8 ± 0.1	0.008	GS599104	0	-	c	LinJ28_V3.1590	Target SNARE, putative	N.D.	
Lin77H11	1.71	0.8 ± 0.3	0.050	GS599105	0	0	c			N.D.	
Lin82G7	2.40	1.3 ± 0.4	0.035	GS599106	0	0	b	LinJ24_V3.0410	Hypothetical protein, conserved	N.D.	
								LinJ24_V3.0420	Cysteine peptidase, Clan CA, family C12, putative	N.D.	
								LinJ24_V3.0430	Hypothetical protein, conserved	N.D.	
Lin102C9	2.09	1.1 ± 0.2	0.013	GS599107	0	0	b	LinJ35_V3.0580	SNF-7-like protein, conserved	N.D.	
								LinJ35_V3.0590	Hypothetical protein, conserved	N.D.	
Lin128A4	1.96	1.0 ± 0.4	0.042	GS599108	0	0	b	LinJ12_V3.0840	Hypothetical protein, conserved	N.D.	
								LinJ12_V3.0850	Arginine N-methyltransferase-like protein	N.D.	
Lin137D12	1.73	0.8 ± 0.1	0.009	GS599109	-	0	c	LinJ14_V3.0380	Hypothetical protein, conserved	N.D.	
Lin139F3	1.75	0.8 ± 0.3	0.031	GS599110	-	0	c	LinJ29_V3.2450	Hypothetical protein, conserved	N.D.	
Lin201H8	2.83	1.5 ± 0.1	0.001	GS599111	0	-	c	LinJ36_V3.3410	Hypothetical protein, conserved	N.D.	
Lin212H6	2.18	1.1 ± 0.2	0.016	GS599112	0	0	b	LinJ28_V3.1290	Hypothetical protein, conserved	N.D.	
								LinJ28_V3.1300	Copine-i-like protein	N.D.	
Lin228C8	1.94	1.0 ± 0.1	0.006	GS599113	0	-	c	LinJ33_V3.0850	Hypothetical protein, conserved	N.D.	
Lin279H12	2.07	1.0 ± 0.2	0.012	GS599114	0	-	c			N.D.	
Lin20E9	-2.33	-1.2 ± 0.1	0.004	GS599115	0	0	c			N.D.	
Lin26A9	-1.84	-0.9 ± 0.1	0.005	GS599116	9e-65	3e-163	a	LinJ26_V3.2290	Nitrilase, putative	N.D.	
								LinJ26_V3.2300	Hypothetical protein, conserved	N.D.	
Lin46H3	-2.16	-1.1 ± 0.2	0.014	GS599117	5e-137	0	c	LinJ27_V3.1950	Branched-chain amino acid aminotransferase, putative	N.D.	
								LinJ27_V3.1960	Hypothetical protein, conserved	N.D.	
								LinJ35_V3.3620	Hypothetical protein, conserved	N.D.	
Lin66G1	-2.05	-1.0 ± 0.3	0.025	GS599118	0	-	c	LinJ34_V3.0820	Serine/theonine-protein phosphatase PP1, putative	N.D.	
Lin87D5	-1.88	-0.9 ± 0.0	0.000		3e-52	7e-93	a	LinJ33_V3.3350	Cation transporter, putative	N.D.	
								LinJ33_V3.3360	Beta prime cop protein, putative	N.D.	
Lin97D2	-2.53	-1.3 ± 0.2	0.010	GS599119	0	1e-153	c	LinJ31_V3.1560	Protein kinase, putative	N.D.	
Lin102E2	-2.05	-1.0 ± 0.2	0.014	GS599120	3e-166	0	c	LinJ18_V3.0720	Hypothetical protein, conserved	N.D.	
Lin113B12	-1.81	-0.9 ± 0.1	0.005	GS599121	0	0	b	LinJ27_V3.0560	Hypothetical protein, conserved	N.D.	
								LinJ27_V3.0570	Hypothetical protein, conserved	N.D.	
								LinJ27_V3.0580	Reductase, putative	N.D.	
Lin122B2	-2.78	-1.5 ± 0.3	0.018	GS599122	0	0	b	LinJ19_V3.1490	Oxidoreductase-like protein	N.D.	
								LinJ19_V3.1500	Hypothetical protein, conserved	N.D.	
Lin126F1	-1.71	-0.8 ± 0.3	0.042	GS599123	0	0	b	LinJ35_V3.2030	Ankyrin repeat protein, putative	N.D.	
								LinJ35_V3.2040	60S ribosomal protein L32	N.D.	
Lin288D2	-2.06	-1.0 ± 0.1	0.001	GS599124	0	0	c	LinJ24_V3.1470	Kinesin, putative	N.D.	
								LinJ31_V3.3250	Phosphatidylethanolaminen-methyltransferase-like protein	N.D.	

Table S3. Genes coding for hypothetical and unknown proteins that are differentially regulated under TS. F < -1.7 indicates gene down-regulation and F > 1.7 up-regulation.

Clone	*F*	*Log$_2$F ± SD*	*p*	*GenBank*	*e-value*		*Def.*	*Id.*	*Annotated Gene Function*	*qRT-PCR*	
					Fw	*Rv*				+/-	F ± SD
Lin21C1	1.87	0.9 ± 0.1	0.009	GS599125	0	0	b	LinJ23_V3.0100	Hypothetical protein, conserved	N.D.	
								LinJ23_V3.0110	Hypothetical protein, unknown function	N.D.	
Lin38F6	1.94	1.0 ± 0.3	0.031	GS599126	0	0	a	LinJ20_V3.0850	Cytochrome c oxydase assembly factor-like protein	-	1.2 ± 0.3
								LinJ20_V3.0860	Hypothetical protein, conserved	N.D.	
**Lin44H5**	2.18	1.1 ± 0.1	0.005	GS599127	0	0	b	LinJ31_V3.0090	Hypothetical protein, conserved	N.D.	
								LinJ31_V3.0100	Hypothetical protein, conserved	N.D.	
Lin49G11	1.84	0.9 ± 0.1	0.002	GS599128	0	0	b	LinJ18_V3.0030	Hypothetical protein, conserved	N.D.	
								LinJ18_V3.0040	Major facilitator superfamily protein, putative	-	-1.3 ± 0.1
								LinJ18_V3.0050	Hypothetical protein, conserved	N.D.	
Lin59F12	1.81	0.9 ± 0.3	0.045	GS599129	0	0	a	LinJ12_V3.0500	Hypothetical protein, conserved	N.D.	
Lin70H12	1.70	0.8 ± 0.3	0.048	GS599130	7e-16	7e-24	a	LinJ31_V3.1110	Hypothetical protein, conserved	N.D.	
Lin77G12	1.87	0.9± 0.0	0.001	GS599131	0	0	b	LinJ32_V3.1720	Hypothetical protein, conserved	N.D.	
Lin78F3	-3.15	-1.7 ± 0.1	0.002	GS599078	0	0	b	LinJ26_V3.0970	Hypothetical protein, conserved	N.D.	
								LinJ26_V3.0980	Hypothetical protein, conserved	N.D.	
Lin89A11	2.00	1.0 ± 0.3	0.022	GS599132	0	0	a	LinJ15_V3.0340	Hypothetical protein, conserved	N.D.	
Lin93A7	2.36	1.2 ± 0.5	0.043	GS599133	3e-67	3e-104	b	LinJ21_V3.0790	Hypothetical protein, conserved	N.D.	
Lin95A3	2.24	1.2 ± 0.4	0.038	GS599134	3e-64	2e-74	b	LinJ21_V3.0790	Hypothetical protein, conserved	N.D.	
Lin111C6	1.74	0.8 ± 0.3	0.034	GS599135	0	0	b	LinJ08_V3.1010	Hypothetical protein, conserved	N.D.	
Lin112B12	2.98	1.6 ± 0.2	0.004	GS599136	0	0	b	LinJ29_V3.0530	Hypothetical protein, conserved	N.D.	
								LinJ29_V3.0540	Hypothetical protein, conserved	N.D.	
Lin135E7	1.77	0.8 ± 0.1	0.010		0	0	a	LinJ13_V3.0250	Hypothetical protein, conserved	N.D.	
								LinJ13_V3.0260	N-acetyltransferase subunit ARD1, putative	-	-1.4 ± 0.1
**Lin135E9**	1.81	0.9 ± 0.2	0.026	GS599137	0	0	a	**LinJ34_V3.0060**	**Hypothetical protein, conserved**	N.D.	
								LinJ34_V3.0070	Ascorbate-dependent peroxidase, putative	-	1.0 ± 0.3
Lin152D8	2.29	1.2 ± 0.4	0.027	GS599138	0	0	b	LinJ33_V3.1660	Ribulose-5-phosphate 3-epimerase, putative	-	1.1 ± 0.0
								LinJ33_V3.1670	Hypothetical protein, conserved	N.D.	
Lin225C2	1.91	0.9 ± 0.2	0.018	GS599139	0	0	a	LinJ28_V3.1610	Hypothetical protein, conserved	N.D.	
								LinJ28_V3.1620	Hypothetical protein, conserved	N.D.	
Lin273G6	1.92	0.9 ± 0.2	0.011	GS599140	0	0	a	LinJ35_V3.4530	Smf-snRNP core complex protein, putative	-	1.4 ± 0.3
								LinJ35_V3.4540	Hypothetical protein, conserved	N.D.	
Lin284F10	2.00	1.0 ± 0.2	0.013	GS599141	0	0	b	LinJ15_V3.1570	Hypothetical protein, conserved	N.D.	
Lin312C2	1.71	0.8 ± 0.2	0.028	GS599142	0	0	b	LinJ27_V3.0120	Hypothetical protein, conserved	N.D.	
								LinJ27_V3.0130	Hypothetical protein, conserved	N.D.	
Lin16H8	-1.82	-0.9 ± 0.3	0.048	GS599143	0	0	a	LinJ34_V3.1740	Hypothetical protein, conserved	N.D.	
Lin43F3	-2.66	-1.4 ± 0.3	0.015	GS599144	2e-105	2e-105	a	LinJ32_V3.0500	Hypothetical protein, conserved	N.D.	
**Lin58A11**	-1.78	-0.8 ± 0.2	0.017	GS599145	8e-176	5e-165	b	**LinJ35_V3.3190**	**Hypothetical protein, conserved**	N.D.	
**Lin74F6**	-1.75	-0.8 ± 0.2	0.027	GS599146	0	1e-134	a	LinJ29_V3.1820	Hypothetical protein, conserved	N.D.	
								LinJ29_V3.1830	Hypothetical protein, conserved	N.D.	
**Lin155G12**	-2.20	-1.1 ± 0.2	0.012	GS599087	2e-154	3e-12	b	LinJ35_V3.3770	Hypothetical protein, conserved	N.D.	
								LinJ35_V3.3780	Hypothetical protein, conserved	N.D.	
**Lin169B8**	-2.69	-1.4 ± 0.2	0.008	GS599147	0	2e-145	b	LinJ24_V3.2320	Hypothetical multi-pass transmembrane protein	N.D.	
								LinJ24_V3.2330	Hypothetical protein, conserved	N.D.	
Lin170G5	-1.76	-0.8 ± 0.3	0.050	GS599148	8e-139	1e-100	b	LinJ36_V3.0380	Hypothetical protein, conserved	N.D.	
								LinJ36_V3.0390	Hypothetical protein, conserved	N.D.	
Lin193H8	-2.17	-1.1 ± 0.1	0.001	GS599149	0	0	a	LinJ06_V3.1350	Hypothetical protein, unknown function	N.D.	
Lin198G3	-2.58	-1.4 ± 0.1	0.003	GS599150	0	0	a	LinJ18_V3.1640	Hypothetical protein, conserved	N.D.	
**Lin209B12**	-2.07	-1.1 ± 0.2	0.013	GS599151	0	0	a	LinJ35_V3.4170	Hypothetical protein, conserved	N.D.	
								LinJ35_V3.4180	Hypothetical protein, conserved	N.D.	
								LinJ35_V3.4190	Hypothetical protein, conserved	N.D.	
Lin212A4	-1.74	-0.8 ± 0.0	0.000	GS599152	0	0	a	LinJ30_V3.2770	Hypothetical protein, conserved	N.D.	
Lin296C4	-1.72	-0.8 ± 0.2	0.022	GS599153	0	0	b	LinJ29_V3.0630	Hypothetical protein, conserved	N.D.	

Table S4. Unresolved clones for TS. These clones fulfil spot selection requirements (see Materials and methods section in the article) but correspond to minicircle sequences. They do not map against already annotated genes in the *L. infantum* genome project sequence (custom Glimmer annotations are indicated whenever predicted) or clone sequence assembly outcome is *c* and no qRT-PCR assay has been performed to find out which gene(s) are differentially regulated. F < -1.7 indicate gene down-regulation and F > 1.7 up-regulation.

Clone	*F*	*Log$_2$F ± SD*	*p*	*GenBank*	*e-value*		*Def.*	*Id.*	*Annotated Gene Function*	*qRT-PCR*	
					Fw	*Rv*				+/-	F ± SD
Lin13C3	1.78	0.8± 0.0	0.000	GS599154	0	-	c	LinJ18_V3.1050	5-oxoprolinase, putative	N.D.	
Lin32E6	1.76	0.8 ± 0.1	0.008	GS599155	0	0	c	LinJ31_V3.3250	Phosphatidylethanolaminen-methyltransferase-like protein	N.D.	
								LinJ14_V3.1430	Hypothetical protein, conserved	N.D.	
Lin93A7	2.36	1.2 ± 0.5	0.043	GS599133	2e-102	3e-104	c			N.D.	
										N.D.	
Lin101D5	1.94	1.0 ± 0.2	0.010	GS599156	0	7e-167	c			N.D.	
										N.D.	
Lin279H12	2.07	1.0 ± 0.2	0.012	GS599157	0	-	c			N.D.	
Lin298F2	1.74	0.8 ± 0.3	0.032	GS599158	1e-106	-	c	LinJ32_V3.1440	Hypothetical protein, conserved	N.D.	
Lin2G12	-1.85	-0.9 ± 0.0	0.001	GS599159	6e-140	-	c	LinJ28_V3.1590	Target SNARE, putative	N.D.	
								LinJ28_V3.1600	Target SNARE, putative	N.D.	
								LinJ28_V3.1610	Hypothetical protein, conserved	N.D.	
Lin10D9	-1.72	-0.8 ± 0.0	0.001	GS599160	0	0	c	LinJ36_V3.0890	Hypothetical protein, conserved	N.D.	
Lin17D4	-1.79	-0.8 ± 0.3	0.039	GS599161	2e-117	-	c	LinJ09_V3.0070	Endonuclease III, putative	N.D.	
Lin22D12	-1.83	-0.9 ±0.3	0.037	GS599162	-	0	c			N.D.	
Lin35A12	-2.10	-1.1 ± 0.4	0.043	GS599163	0	0	a	LinJ30_V3.2370	Zinc-finger protein, conserved	N.D.	
								LinJ30_V3.2380	ADP-ribosylation factor-like protein	N.D.	
Lin99G11	-2.54	-1.3 ± 0.0	0.000	GS599164	-	0	c	LinJ30_V3.2380	ADP-ribosylation factor-like protein	N.D.	
Lin102E2	-1.96	-1.0 ± 0.2	0.018	GS599165	0	0	c	LinJ18_V3.0720	Hypothetical protein, conserved	N.D.	
Lin163F5	-1.99	-1.0 ± 0.3	0.022	GS599166	0	0	b	LinJ30_V3.2380	ADP-ribosylation factor-like protein	N.D.	
								LinJ30_V3.2390	Hypothetical protein, conserved	N.D.	

Table S5. Genes coding for hypothetical and unknown proteins that are differentially regulated under PS. F < -1.7 indicates gene down-regulation and F > 1.7 up-regulation.

Clone	*F*	*Log_2F ± SD*	*p*	*GenBank*	*e-value*		*Def.*	*Id.*	*Annotated Gene Function*	*qRT-PCR*	
					Fw	*Rv*				+/-	F ± SD
Lin56D12	2.97	1.6 ± 0.3	0.003	GS599167	0	0	a	LinJ07_V3.0140	Hypothetical protein, conserved	N.D.	
Lin61C12	1.71	0.8 ± 0.0	0.004	GS599168	0	0	b	LinJ35_V3.0190	Hypothetical protein, conserved	N.D.	
Lin62D4	2.19	1.1 ± 0.1	0.009	GS599169	0	0	b	LinJ29_V3.1940	Hypothetical protein, conserved	N.D.	
Lin83E8	2.04	1.0 ± 0.0	0.001	GS599170	0	0	a	LinJ35_V3.0140	Hypothetical protein, conserved	N.D.	
								LinJ35_V3.0150	Hypothetical protein, conserved	N.D.	
								LinJ35_V3.0160	Hypothetical protein, conserved	N.D.	
Lin84E12	2.29	1.2 ± 0.2	0.019	GS599171	0	0	a	LinJ35_V3.0140	Hypothetical protein, conserved	N.D.	
								LinJ35_V3.0150	Hypothetical protein, conserved	N.D.	
								LinJ35_V3.0160	Hypothetical protein, conserved	N.D.	
Lin87A10	2.28	1.2 ± 0.1	0.003	GS599172	0	0	a	LinJ29_V3.2870	Hypothetical protein, conserved	N.D.	
Lin92D4	2.20	1.1 ± 0.1	0.006	GS599173	0	0	a	LinJ26_V3.1140	Hypothetical protein, conserved	N.D.	
								LinJ26_V3.1150	Hypothetical protein, conserved	N.D.	
Lin96H11	1.89	0.9 ± 0.1	0.001	GS599174	2e-151	1e-137	a	LinJ26_V3.1570	Hypothetical protein, unknown function	N.D.	
Lin100C10	1.72	0.8 ± 0.0	0.003	GS599175	0	0	b	LinJ10_V3.1350	Hypothetical protein	N.D.	
								LinJ10_V3.1360	Hypothetical protein, conserved	N.D.	
								LinJ10_V3.1370	Hypothetical protein, conserved	N.D.	
Lin105C1	1.89	0.9 ± 0.1	0.025	GS599176	0	0	a	LinJ22_V3.0610	Hypothetical protein, conserved	N.D.	
Lin117G9	1.88	0.9 ± 0.1	0.006	GS599177	0	0	a	LinJ26_V3.1720	Hypothetical protein, conserved	N.D.	
Lin155H9	1.88	0.9 ± 0.1	0.008	GS599178	0	1e-72	b	LinJ28_V3.1140	Hypothetical protein, conserved	N.D.	
Lin194A1	1.93	0.9 ± 0.0	0.001	GS599179	0	0	b	LinJ35_V3.5110	Hypothetical protein, unknown function	N.D.	
Lin206E10	1.70	0.8 ± 0.1	0.024	GS599180	0	0	a	LinJ07_V3.0020	Hypothetical protein, conserved	N.D.	
Lin240C7	1.90	0.9 ± 0.0	0.001	GS599181	6e-124	2e-130	b	LinJ26_V3.2200	Hypothetical protein, conserved	N.D.	
Lin244H6	2.26	1.2 ± 0.0	0.003	GS599182	0	0	b	LinJ22_V3.0620	Hypothetical protein, conserved	N.D.	
Lin255B11	1.91	0.9 ± 0.1	0.002	GS599183	4e-97	7e-93	a	LinJ31_V3.2830	Hypothetical protein, conserved	N.D.	
Lin267H4	1.73	0.8 ± 0.1	0.003	GS599184	0	0	b	LinJ17_V3.0280	Hypothetical protein, conserved	N.D.	
								LinJ17_V3.0290	Hypothetical protein, conserved	N.D.	
Lin268E7	1.91	0.9 ± 0.1	0.004	GS599185	0	0	b	LinJ26_V3.2300	Hypothetical protein, conserved	N.D.	
Lin270A3	2.19	1.1 ± 0.1	0.036	GS599186	0	0	b	LinJ09_V3.0020	Hypothetical protein, conserved	N.D.	
								LinJ09_V3.0030	Hypothetical protein, conserved	N.D.	
Lin276F12	2.45	1.3 ± 0.1	0.004	GS599187	0	0	b	LinJ34_V3.0730	Hypothetical protein, conserved	N.D.	
								LinJ34_V3.0740	Hypothetical protein, conserved	N.D.	
Lin284D12	2.48	1.3 ± 0.1	0.001	GS599188	0	0	b	LinJ27_V3.0130	Hypothetical protein, conserved	N.D.	
								LinJ27_V3.0140	Hypothetical protein, conserved	N.D.	
Lin88A1	-3.51	1.8 ± 0.1	0.004	GS599189	0	2e-176	b	LinJ27_V3.2470	Hypothetical protein, conserved	N.D.	
Lin95D8	-5.40	2.4 ± 0.2	0.010	GS599190	4e-165	2e-105	b	LinJ27_V3.2470	Hypothetical protein, conserved	N.D.	
Lin181H11	-3.18	1.7 ± 0.1	0.001	GS599191	0	0	b	LinJ18_V3.0720	Hypothetical protein, conserved	N.D.	
Lin201C8	-2.14	1.1 ± 0.1	0.006	GS599192	0	0	a	LinJ30_V3.1540	Hypothetical protein, conserved	N.D.	

Table S6. Unresolved clones for PS. These clones fulfil spot selection requirements (see Materials and methods section in the article) but correspond to minicircle sequences. They do not map against already annotated genes in the *L. infantum* genome project sequence (custom Glimmer annotations are indicated whenever predicted) or clone sequence assembly outcome is *c* and no qRT-PCR assay has been performed to find out which gene(s) are differentially regulated. F < -1.7 indicates gene down-regulation and F > 1.7 up-regulation.

Clone	*F*	*Log_2F ± SD*	*p*	*GenBank*	*e-value*		*Def.*	*Id.*	*Annotated Gene Function*	*qRT-PCR*	
					Fw	*Rv*				+/-	F ± SD
Lin10A7	1.70	0.8 ± 0.0	0.002	GS599193	0	0	b	LinJ28_V3.0050	Dual-specificity protein phosphatase, putative	N.D.	
								LinJ28_V3.0060	Calmodulin-like protein	N.D.	
								LinJ28_V3.0070	RNA polymerase B subunit RPB8, putative	N.D.	
Lin13G4	1.92	0.9 ± 0.1	0.009	GS599194	0	0	c	LinJ36_V3.0350	Hypothetical protein, conserved	N.D.	
								LinJ29_V3.0990	Aspartic peptidase, Clan AD, family A22B, putative	N.D.	
Lin16E1	1.77	0.8 ± 0.1	0.050	GS599195	-	0	c	LinJ35_V3.0180	Hypothetical protein, conserved	N.D.	
								LinJ35_V3.0190	Hypothetical protein, conserved	N.D.	
Lin21D12	1.96	0.9 ± 0.1	0.001	GS599196	0	0	b	LinJ29_V3.2450	Hypothetical protein, conserved	N.D.	
								LinJ29_V3.2460	Hypothetical protein, conserved	N.D.	
								LinJ29_V3.2470	Metallo-peptidase, Clan-MH, family M20	N.D.	
Lin24H3	1.84	0.9 ± 0.1	0.024	GS599197	0	-	c	LinJ29_V3.2310	GTP-binding protein, putative	N.D.	
Lin33C4	2.54	1.3 ± 0.1	0.002	GS599198	0	0	c	LinJ22_V3.0340	40S ribosomal protein S15, putative	N.D.	
Lin34D12	1.84	0.9 ± 0.1	0.003	GS599199	0	0	a	LinJ33_V3.0940	dnaJ chaperone-like protein	N.D.	
								LinJ33_V3.0950	Hypothetical protein, conserved	N.D.	
								LinJ33_V3.0960	40S ribosomal protein S3, putative	N.D.	
Lin37A10	4.84	2.3 ± 0.1	0.004	GS599200	0	-	c	LinJ32_V3.3560	Hypothetical protein, conserved	N.D.	
Lin45E7	2.28	1.2 ± 0.2	0.006	GS599201	0	0	b	LinJ30_V3.3380	Hypothetical protein, conserved	N.D.	
								LinJ30_V3.3390	60S ribosomal protein L9, putative	N.D.	
Lin48E3	1.93	0.9 ± 0.1	0.008	GS599202	0	0	a	LinJ28_V3.1030	Oxidoreductase-like protein	N.D.	
								LinJ28_V3.1040	Hypothetical protein, conserved	N.D.	
								LinJ28_V3.1050	40S ribosomal protein S14	N.D.	
Lin50E2	2.34	1.2 ± 0.2	0.002	GS599203	5e-20	0	b	LinJ07_V3.0540	Hypothetical protein, conserved	N.D.	
								LinJ07_V3.0550	60S ribosomal protein L7a, putative	N.D.	
Lin53D8	1.94	0.9 ± 0.1	0.005	GS599204	0	0	b	LinJ30_V3.3700	Hypothetical protein, conserved	N.D.	
								LinJ30_V3.3710	60S ribosomal protein L15	N.D.	
Lin54C2	2.07	1.0 ± 0.0	0.001	GS599205	0	0	a	LinJ24_V3.2410	Mitogen-activated protein kinase	N.D.	
								LinJ24_V3.2420	Hypothetical protein, conserved	N.D.	
								LinJ24_V3.2430	Hypothetical protein, conserved	N.D.	
Lin61F2	1.74	0.8 ± 0.0	0.003	GS599206	0	0	b	LinJ15_V3.0220	60S ribosomal protein L13a	N.D.	
								LinJ15_V3.0230	Hypothetical protein, conserved	N.D.	
								LinJ15_V3.0240	Protein phosphatase 1, catalytic subunit, putative	N.D.	
Lin93D4	2.00	1.0 ± 0.1	0.008	GS599207	4e-165	0	b	LinJ29_V3.0950	ADP-ribosylation factor 3	N.D.	
								LinJ29_V3.0960	Hypothetical protein, conserved	N.D.	

Lin96H7	1.91	0.9 ± 0.0	0.001	GS599208	0	0	b	LinJ31_V3.3310	Hypothetical protein, unknown function	N.D.	
								LinJ31_V3.3320	Histone H4	N.D.	
Lin100E4	1.70	0.8 ± 0.1	0.005	GS599209	0	0	a	LinJ28_V3.2280	Dynein light chain lc6, flagellar outer arm, putative	N.D.	
								LinJ28_V3.2290	A/G-specific adenine glycosylase, putative	N.D.	
Lin117A9	3.54	1.8 ± 0.4	0.042	GS599210	0	-	c			N.D.	
Lin135B3	1.92	0.9 ± 0.1	0.004	GS599211	0	0	b	LinJ30_V3.0750	Hypothetical protein, conserved	N.D.	
								LinJ30_V3.0760	Co-chaperone GrpE, putative	N.D.	
								LinJ30_V3.0770	Unknown	N.D.	
Lin178D3	2.28	1.2 ± 0.2	0.006	GS599212	0	0	c	LinJ06_V3.1240	Hypothetical protein, conserved	N.D.	
								LinJ29_V3.2470	Aspartyl aminopeptidase metallo-peptidase, Clan MH, family M20	N.D.	
Lin181G9	1.89	0.9 ± 0.0	0.001	GS599213	0	0	c	LinJ06_V3.1240	Hypothetical protein, conserved	N.D.	
								LinJ29_V3.2450	Hypothetical protein, conserved	N.D.	
								LinJ29_V3.2460	Hypothetical protein, conserved	N.D.	
Lin231C8	1.82	0.9 ± 0.1	0.004	GS599214	0	0	a	LinJ36_V3.3920	Hypothetical protein, conserved	N.D.	
								LinJ36_V3.3930	60S ribosomal protein L34, putative	N.D.	
Lin238F10	2.43	1.3 ± 0.0	0.003	GS599215	0	0	b	LinJ33_V3.1410	Cysteine conjugate beta-lyase, aminotransferase-like protein	N.D.	
								LinJ33_V3.1420	Syntaxin-like protein	N.D.	
								LinJ33_V3.1430	Hypothetical protein, conserved	N.D.	
Lin239C6	2.33	1.2 ± 0.3	0.046	GS599216	1e-128	0	b	LinJ35_V3.1540	Reiske iron-sulfur protein precursor, putative	N.D.	
								LinJ35_V3.1550	Hypothetical protein, conserved	N.D.	
Lin248E6	1.98	1.0 ± 0.1	0.001	GS599217	0	0	c	LinJ02_V3.0270	ABC1 transporter, putative	N.D.	
								LinJ09_V3.1400	Hypothetical protein, conserved	N.D.	
Lin265C7	1.85	0.9 ± 0.1	0.008	GS599218	0	0	a	LinJ35_V3.3840	60S ribosomal protein L23, putative	N.D.	
								LinJ35_V3.3850	Hypothetical protein, conserved	N.D.	
								LinJ35_V3.3860	Hypothetical protein, conserved	N.D.	
Lin276E12	1.76	0.8 ± 0.0	0.001	GS599219	6e-161	0	a	LinJ36_V3.0550	Hypothetical protein, conserved	N.D.	
								LinJ36_V3.0560	Protein phosphatase 2C-like protein	N.D.	
								LinJ36_V3.0570	Small nuclear ribonucleoprotein	N.D.	
Lin284F11	1.77	0.8 ± 0.1	0.009	GS599220	1e-165	0	b	LinJ35_V3.1540	Reiske iron-sulfur protein precursor, putative	N.D.	
								LinJ35_V3.1550	Hypothetical protein, conserved	N.D.	
Lin288C8	1.94	0.9 ± 0.1	0.007	GS599221	0	1e-122	b	LinJ27_V3.1120	Histone H1, putative	N.D.	
								LinJ27_V3.1130	Carboxypeptidase, putative	N.D.	
Lin298H9	2.59	1.4 ± 0.1	0.013	GS599222	0	0	a	LinJ35_V3.3960	Hypothetical protein, conserved	N.D.	
Lin303D2	2.62	1.4 ± 0.1	0.006	GS599223	0	0	b	LinJ07_V3.0420	Homoserine dehydrogenase-like protein	N.D.	
								LinJ07_V3.0430	Acetylornithine deacetylase-like protein	N.D.	
Lin308A8	2.64	1.4 ± 0.2	0.036	GS599224	0	0	b	LinJ30_V3.0700	40S ribosomal protein S30, putative	N.D.	
								LinJ30_V3.0710	40S ribosomal protein S30, putative	N.D.	
								LinJ30_V3.0720	NUDC-like protein	N.D.	
								LinJ30_V3.0730	Hypothetical protein, conserved	N.D.	
								LinJ30_V3.0740	CDC16, putative	N.D.	
Lin102G3	-3.15	1.7 ± 0.1	0.001	GS599225	0	0	b	LinJ24_V3.0200	Transcription elongation factor, putative	N.D.	
								LinJ24_V3.0210	Hypothetical protein, unknown function	N.D.	
Lin198B2	-1.92	0.9 ± 0.1	0.008	GS599226	-	5e-171	c	LinJ11_V3.0200	Hypothetical protein, conserved	N.D.	
Lin228F6	-3.47	1.8 ± 0.2	0.036	GS599227	0	0	a	LinJ32_V3.1740	Hypothetical protein, conserved	N.D.	
Lin228H3	-1.84	0.9 ± 0.2	0.024	GS599228	3e-163	0	a	LinJ31_V3.3250	Phosphatidylethanolaminen-methyltransferase-like protein	N.D.	
								LinJ31_V3.3260	Methylcrotonyl-CoA carboxylase biotinylated subunit protein-like protein	N.D.	
Lin290B4	-2.40	1.3 ± 0.1	0.002	GS599229	0	6e-124	c	LinJ27_V3.2470	Hypothetical protein, conserved	N.D.	

Table S7. Clones that probably contain up-regulated gRNA genes from minicircle sequences under TPS.

Clone	*F*	*Log_2F ± SD*	*p*	*GenBank*	*Content*
Lin13F11	1.87	0.9 ± 0.3	0.044	GS599230	Contig 957. Possible minicircle sequence.
Lin100B6	1.80	0.8 ± 0.2	0.024	GS599231	Contig 692. Possible minicircle sequence.
Lin133H11	1.79	0.8 ± 0.3	0.048	GS599232	Contig 200. Possible minicircle sequence.
Lin129G10	1.75	0.8 ± 0.3	0.031	GS599233	Contig 957. Possible minicircle sequence.
Lin199G2	1.95	1.0 ± 0.2	0.016	GS599234	Contig 957. Possible minicircle sequence.
Lin228A8	1.77	0.8 ± 0.2	0.026	GS599235	Contig 957. Possible minicircle sequence.
Lin233A12	1.77	0.8 ± 0.2	0.045	GS599236	Contig 957. Possible minicircle sequence.
Lin243H9	1.79	0.8 ± 0.4	0.048	GS599237	Contig 957. Possible minicircle sequence.
Lin245A6	1.99	1.0 ± 0.0	0.000	GS599238	Contig 957. Possible minicircle sequence.
Lin245C8	1.73	0.8 ± 0.2	0.028	GS599239	Contig 957. Possible minicircle sequence.
Lin248E7	1.82	0.9 ± 0.3	0.048	GS599240	Contig 957. Possible minicircle sequence.
Lin251A4	1.71	0.8 ± 0.2	0.026	GS599241	Contig 878. Possible minicircle sequence.
Lin266B7	1.71	0.8 ± 0.3	0.040	GS599242	Contig 957. Possible minicircle sequence.
Lin269A8	2.31	1.2 ± 0.1	0.003	GS599243	Contig 957. Possible minicircle sequence.
Lin276A11	1.73	0.8 ± 0.2	0.019	GS599244	Contig 957. Possible minicircle sequence.
Lin276B2	1.78	0.8 ± 0.2	0.014	GS599245	Contig 957. Possible minicircle sequence.
Lin276C2	1.75	0.8 ± 0.3	0.047	GS599246	Contig 957. Possible minicircle sequence.
Lin278E6	2.10	1.1 ± 0.3	0.022	GS599247	Contig 878. Possible minicircle sequence.
Lin310H6	1.95	1.0 ± 0.4	0.049	GS599248	Contig 692. Possible minicircle sequence.

Table S8. Clones that probably contain up-regulated gRNA genes from minicircle sequences under PS.

Clone	*F*	*$Log_2F \pm SD$*	*P*	*GenBank*	*Content*
Lin24H3	6.28	2.7 ± 0.4	0.021	GS599197	Contig 957. Possible minicircle sequence.
Lin91D8	2.18	1.1 ± 0.0	0.000	GS599249	Contig 957. Possible minicircle sequence.
Lin137H1	2.70	1.4 ± 0,1	0.002	GS599250	Contig 957. Possible minicircle sequence.
Lin166C12	3.24	1.7 ± 0.1	0.001	GS599251	Contig 957. Possible minicircle sequence.
Lin210F10	12.41	3.6 ± 0.3	0.002	GS599252	Contig 957. Possible minicircle sequence.
Lin233A12	7.58	2.9 ± 0.2	0.001	GS599253	Contig 957. Possible minicircle sequence.
Lin239F3	8.92	3.2 ± 0.2	0.005	GS599254	Contig 878. Possible minicircle sequence.
Lin245F3	4.41	2.1 ± 0.1	0.001	GS599255	Contig 957. Possible minicircle sequence.
Lin269A8	4.69	2.2 ± 0.1	0.003	GS599256	Contig 957. Possible minicircle sequence.
Lin274D10	3.98	2.0 ± 0.3	0.014	GS599257	Contig 957. Possible minicircle sequence.
Lin276A11	1.81	0.9 ± 0.1	0.002	GS599258	Contig 957. Possible minicircle sequence.
Lin280H3	2.68	1.4 ± 0.2	0.003	GS599259	Contig 957. Possible minicircle sequence.
Lin285B2	1.76	0.8 ± 0.0	0.000	GS599260	Contig 957. Possible minicircle sequence.
Lin287B7	2.04	1.0 ± 0.1	0.001	GS599261	Contig 957. Possible minicircle sequence.
Lin305B6	6.36	2.7 ± 0.2	0.004	GS599262	Contig 957. Possible minicircle sequence.
Lin310D6	3.85	1.8 ± 0.0	0.000	GS599263	Contig 957. Possible minicircle sequence.
Lin312F1	3.08	1.5 ± 0.2	0.017	GS599264	Contig 957. Possible minicircle sequence.
