# Supplementary material for: Temperature increase prevails over acidification in gene expression modulation of amastigote differentiation in Leishmania infantum
Source: BMC Genomics. 2010 Jan 14;11:31. doi: 10.1186/1471-2164-11-31 (PMC2845110; doi:10.1186/1471-2164-11-31)

ADDITIONAL FILE 4

Figure S3. GO combined molecular function DAG of genes up-regulated under TPS. Adapted from the corresponding DAG created with BLAST2GO. In this graph, each node represents a GO molecular function with an alpha score, both inside a box. Thick-lined boxes represent GO terms directly associated with the analysed set of genes, while thin-lined boxes correspond to GO terms that are not directly annotated for any of the genes but are parents in the ontology of the specific GO terms annotated for them. Each GO number appears above the thick-lined boxes to the right of a code preceded by uTPS, which relates the node with gene annotations in Table 1.

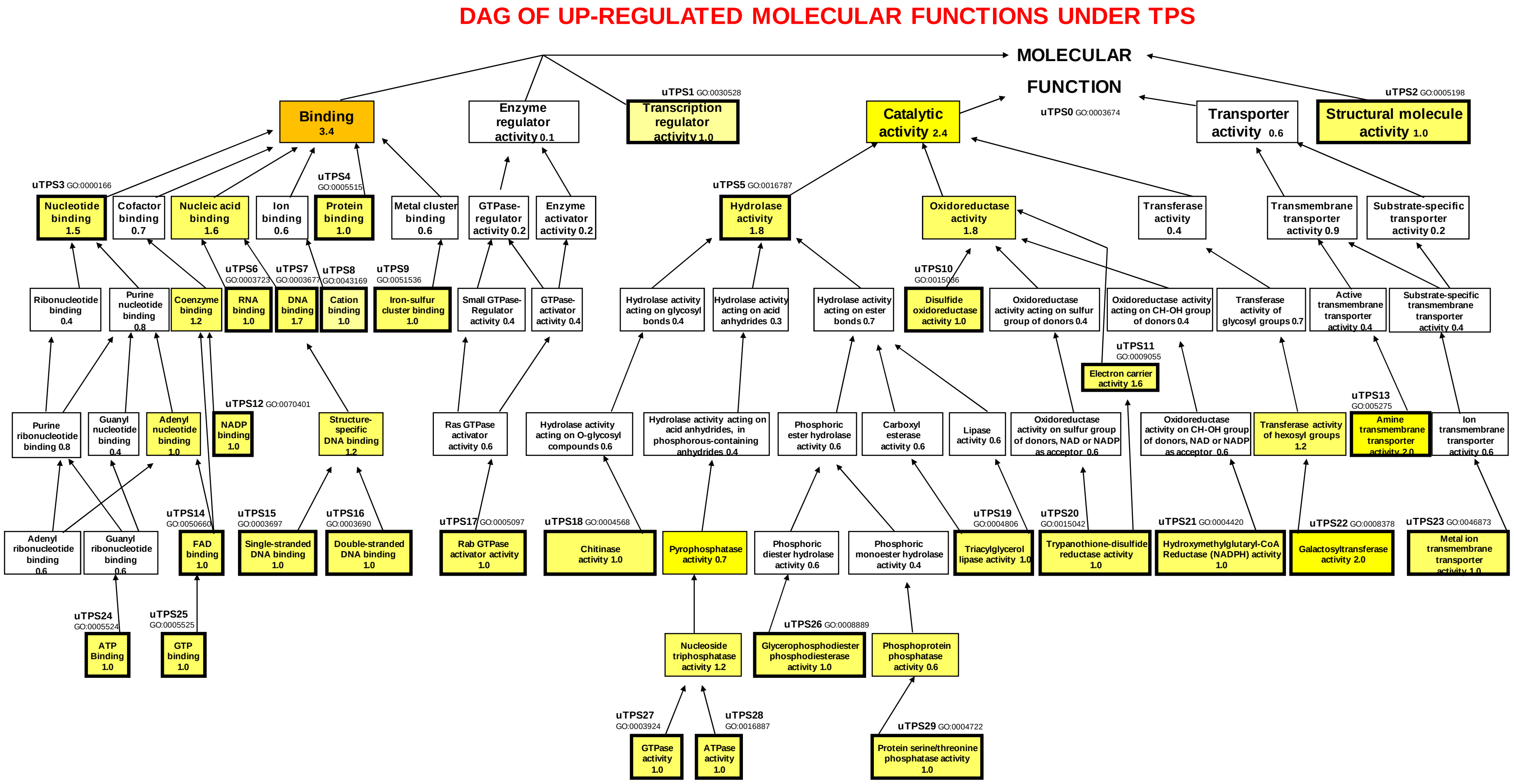

Figure S4. GO combined molecular function DAG of genes down-regulated under TPS. Adapted from the corresponding DAG created with BLAST2GO. In this graph, each node represents a GO molecular function with an alpha score, both inside a box. Thick-lined boxes represent GO terms directly associated with the analysed set of genes, while thin-lined boxes correspond to GO terms that are not directly annotated for any of the genes but are parents in the ontology of the specific GO terms annotated for them. Each GO number appears above the thick-lined boxes to the right of a code preceded by dTPS, which relates the node with gene annotations in Table 2.

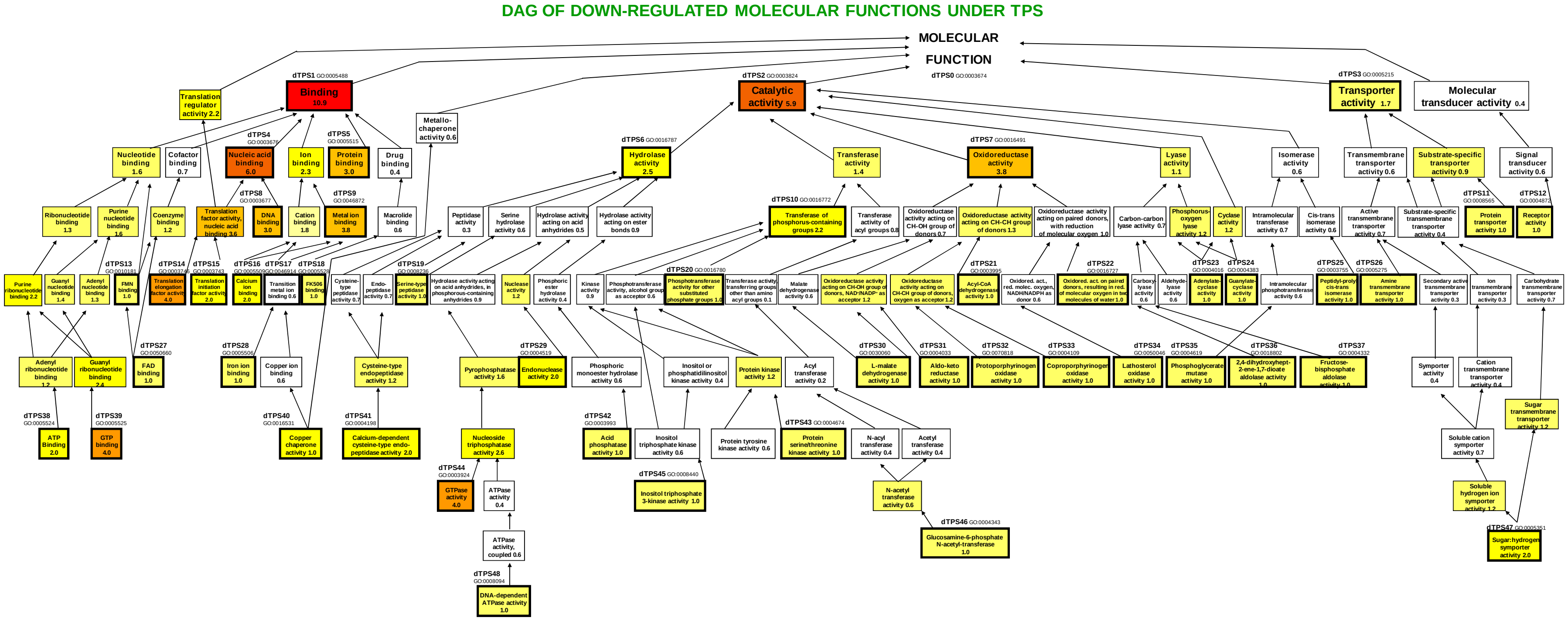

Supplement: Additional file 4 — DAGs (BLAST2GO output). Figures. S3 and S4. GO codes for functions directly annotated on differentially regulated genes found in this study. Each GO code is associated to a custom code to find annotations on genes in Tables 1, 2, 3, 4 and 5. [file 1471-2164-11-31-S4.PDF]
