# Supplementary material for: Temperature increase prevails over acidification in gene expression modulation of amastigote differentiation in Leishmania infantum
Source: BMC Genomics. 2010 Jan 14;11:31. doi: 10.1186/1471-2164-11-31 (PMC2845110; doi:10.1186/1471-2164-11-31)

ADDITIONAL FILE 5

Figure S5. Clusters of genes differentially regulated under TPS, TS and PS. Fourteen clusters have been observed according to the gene expression profile. In this additional figure, clone identification has been included for each box. Differential regulation of genes corresponding to these clones are described in tables 1, 2 and 3, and in the additional file 3.

Clones with significant differences between experimental groups (SAM)

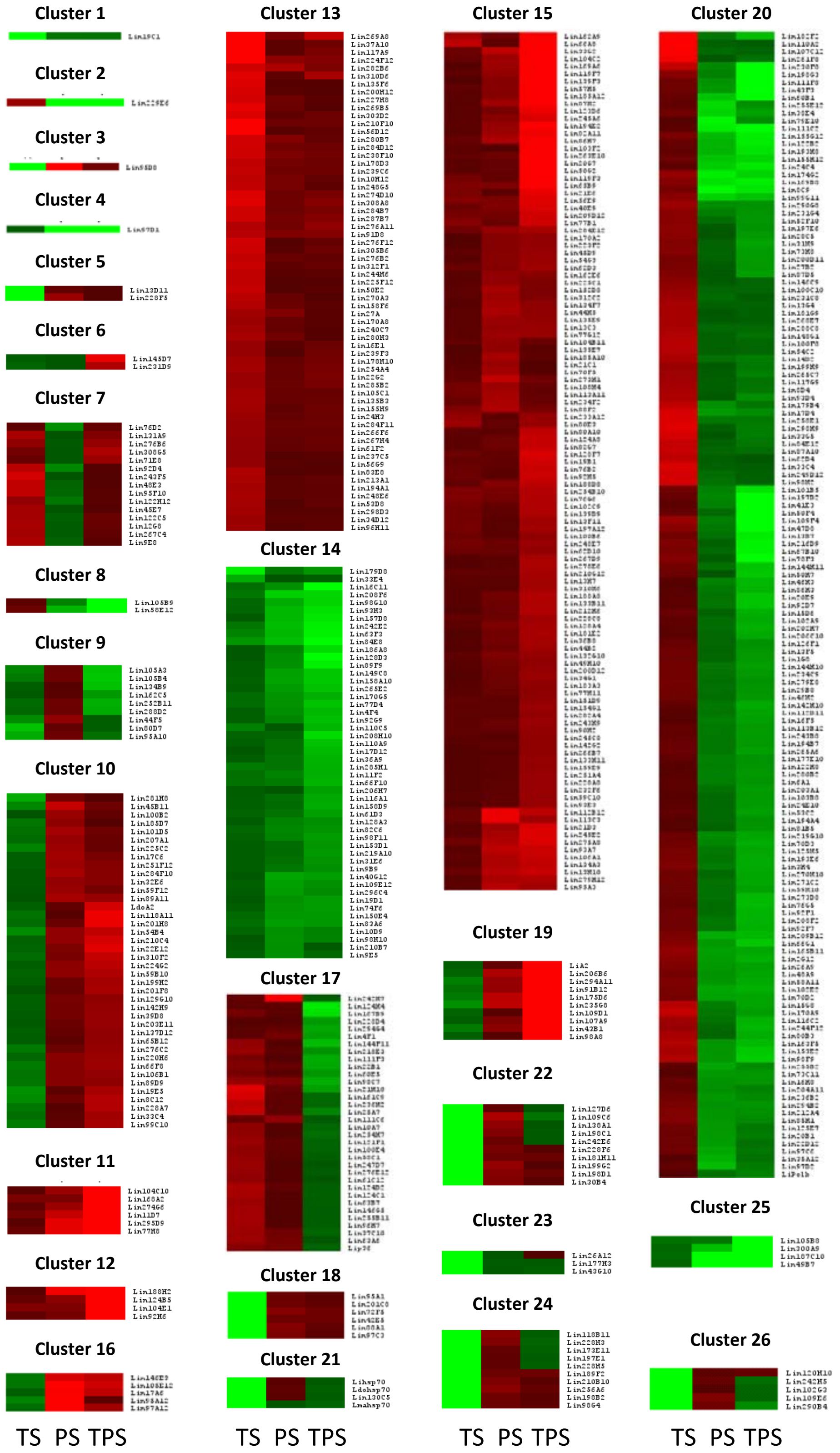

Clones without significant differences between experimental groups (SAM)

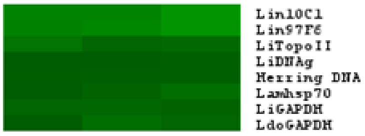

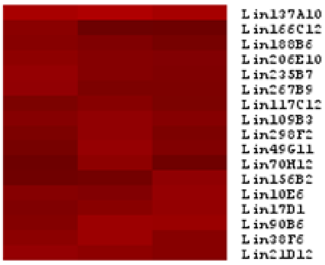

Supplement: Additional file 5 — 3'NT/Nase and GT alignments and oligonucleotides for qRT-PCR. Figure S6 (GT and 3'NT/Nase alignments) and Additional file 3: Table S9 (oligonucleotides for qRT-PCR). [file 1471-2164-11-31-S5.PDF]
