# Supplementary material for: Temperature increase prevails over acidification in gene expression modulation of amastigote differentiation in Leishmania infantum
Source: BMC Genomics. 2010 Jan 14;11:31. doi: 10.1186/1471-2164-11-31 (PMC2845110; doi:10.1186/1471-2164-11-31)
Supplement: Additional file 6 — Fold change clusters of differentially regulated genes including clone names. Figure S5. Supplementary information for Figure 4 identifying the profiles with the clone numbers. [file 1471-2164-11-31-S6.PDF]

## ADDITIONAL FILE 6

**Figure S6. Amino acid sequence alignments of gene copies in tandem that differ in the N-terminus or internal regions.** (A) Glucose transporters. (B) 3’ nucleotidase/nuclease.

**A**

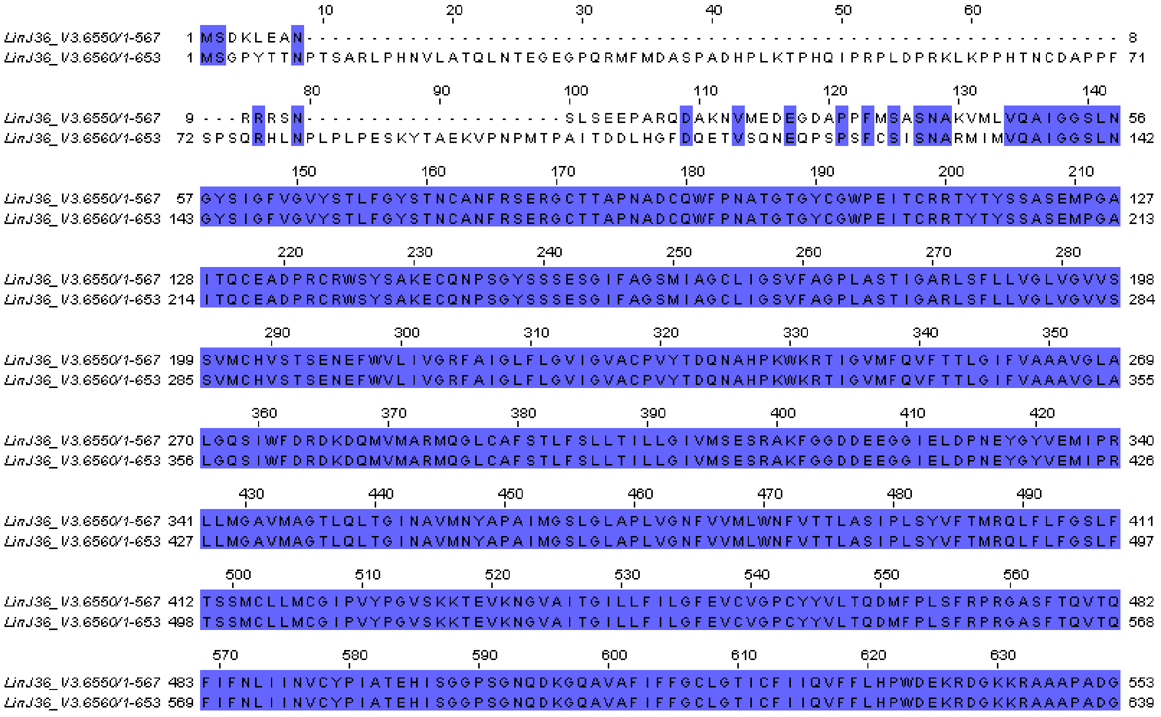

**B**

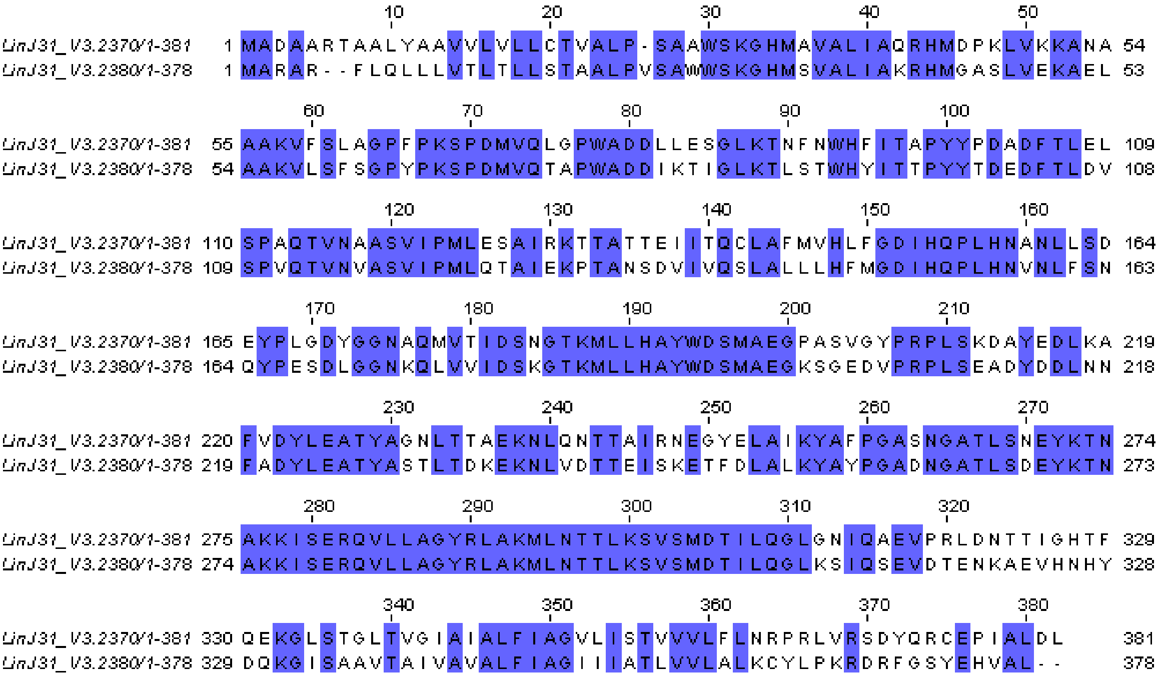

**Table S9**. **Primers for qRT-PCR reactions for TPS, TS, PS.** Annealing temperature for each oligonucleotide pair was optimized by temperature gradients checked by agarose gel electrophoresis, ensuring the absence of primer dimmers and unspecific sequences together with melting curve analysis. Sequence specificity was checked by product sequencing. Thermal cycling was: 95 ºC for 5’ + 40x (95 ºC for 30’’ + 50-60ºC for 30’’ + 72ºC for 30’’ data acquisition) + 72 ºC for 5’ + melting curve [95 ºC for 1’ + 80x (55→95 ºC for 10’’ (+0.5 ºC/step) with data acquisition)].

| Annotation | Annotated gene function | Forward (5'-3') | Reverse (5'-3') |
|---|---|---|---|
| LinJ31_V3.0460 | Amastin, putative | GCATTTCCGCATAGGCCAGA | CAGAGCCAGCGGAGCAAAGA |
| LinJ24_V3.1240 | Translation factor SUI1, putative | GTTGATGCGGCGGAAGTTAA | ACCGTCGAGGCCATTGTGAA |
| LinJ22_V3.0480 | Ubiquitin-conjugating enzyme-like protein | GACGAAGAGCTGGCCGAGAT | TTCAGCAGCGCCACGATCAT |
| LinJ33_V3.2470 | Succinyl-CoA:3-ketoacid-CoA transferase, mitochondrial precursor, putative | CGGCCACATGAACCTGACGAT | GCGAACACCTCTCCACAAGCT |
| LinJ05_V3.0350 | Trypanothione reductase | ACGGCGAGGTTCTGGGTGTT | TCCGATGGTGCTGTGGAAGT |
| LinJ17_V3.1150 | Esterase-like protein | TCGGCAGCTTCACCGATGGT | TGACTCACCATGCGCTGCTT |
| LinJ08_V3.0700/10 | Amastin-like protein | GGTCAGGCGTTCGCTGTCAT | CGGAAGCCAGAGCAGCAGTA |
| LinJ30_V3.0640 | Ribosome biogenesis regulatory protein (RRS1), putative | CGAGGTGAAGCCTGGGTACA | CGCTTGTTGTGCTCCTCCTT |
| LinJ28_V3.2060 | Zinc transporter, putative | GAGCTTGCGCTGATGCTTGT | GTGCAGGTCGTGCGAGAAGT |
| LinJ31_V3.0590 | Amino acid transporter aATP11, putative | GCTTTTTGTGCCGAACGTGAA | CCACCCTCCAGCGTACATCA |
| LinJ14_V3.1440 | Pteridine transporter | GCCGCTGACGAAGCCACTGA | TCGCGCTTCGTGTCGTTGAA |
| LinJ14_V3.1450 | Myo-inositol-1-phosphate synthase | CGCCTCACAGCGCATTTGTCT | ACCGGGACAACCGAGGAAGA |
| LinJ30_V3.0630 | Nitrate reductase, putative | GGCCGCACGTCTGGGTACTT | CGCGTCAGAGGAGATGGAGT |
| LinJ22_V3.0680 | 3’a2rel-related protein | ACGCGGTACAGCGAAAGGT | CAGCGTCGAGGTTCAGCCAA |
| LinJ31_V3.0430 | Cysteine peptidase, Clan CA, family C2, putative | TTGCCCAATACCCGCATTTT | TGTACGCCACATCCGAGGAA |
| LinJ23_V3.1400 | Coronin, putative | CACAGCGGTGCATCTGGTGT | GACTGAGTAGGGCCTTTCCA |
| LinJ22_V3.1310 | I/6 autoantigen-like protein | CGAGGAGGTGGACGCTCTCT | CGCACACCCAGGTCACCATA |
| LinJ35_V3.1230 | Short chain dehydrogenase, putative | CACCAAGTCCGCTGTCAACAT | TGCCTCCGTAGATTGAGCA |
| LinJ26_V3.1000 | Dynein heavy chain, putative | TCTTTCCGCAAGGGTTTCTCA | TCCTCCGGCAGCTCCTAACA |
| LinJ23_V3.1560 | Lathosterol oxidase-like protein | TCCACCACGAACTGTCCAACT | CTCGTACACGGGGTCCACATA |
| LinJ06_V3.1330 | Coproporphyrinogen III oxidase, putative | CCGGCGCAAGAACACACCATA | GACTGGAGGCCGAACTTCGT |
| LinJ06_V3.1340 | Protoporphyrinogen oxidase-like protein | GAAGGCGAATGCAGCAGAGAA | GCCGCTGACATGCCGACTAT |
| LinJ28_V3.2380 | 2,3-bisphosphoglycerate-independent phosphoglycerate mutase-like protein | GCTACAGGCGGGACGCATAAT | GAGTGGAATGATCCGCGATGA |
| LinJ28_V3.2390 | Cyclin dependent kinase-binding protein, putative | ATCGACCCGACAGAGCTGCAT | CGTCTCGGCGTTGTTGAAACT |
| LinJ31_V3.2370 | 3’-nucleotidase/nuclease, putative | GGCTGAGGTGCACAACCACT | GGGCGACGTGCTCATAGGAA |
| LinJ31_V3.2380 | 3’nucleotidase/nuclease precursor, putative | CAACACCACCATTGGGCACA | TAAATCCAGTGCGATCGGCT |
| LinJ36_V3.6550 | Glucose transporter lmgt2, putative | TGACGATCCTGCTTGGGATT | CAGGCCAAGACTGCCCATGA |
| LinJ36_V3.6560 | Glucose transporter, putative | CGGCAAGGCTCCCTCATAAT | CGAGGAATCTGGTGCGGTGT |
| LinJ06_V3.1320 | Pteridine transporter, putative | CTGGGTGACGCGATTGTGTA | GCTGTTGAAGCCAGCCAAGA |
| LinJ30_V3.2780 | Superoxide dismutase, putative | CCCTATCGGCAGCGACTTGA | GCCGCAAGTGGGTAGTCGTA |
| LinJ09_V3.0650 | Serine peptidase family S51, peptidase E, putative | GCAATACCTCGCCCACTTCA | CAAACGGATCCAGCACCACT |
| LinJ07_V3.0150 | Acyl-CoA dehydrogenase, mitochondrial precursor, putative | GGGGGAACCGTTAGCTTTGA | GGCCGCGTAGACGTACTGCT |
| LinJ07_V3.0170 | Maoc family protein | GATTGCCGAAGTCACCCAGTT | TCGCTCGGGTTCTTAGGGTCT |
| LinJ07_V3.0940 | Cytochrome b5-like protein | TGCACAACCTCGTGCTGGAT | GCCGGCGTAGTCAAAGATGA |
| LinJ31_V3.1240 | Vacuolar-type proton translocating pyrophosphatase 1, putative | ATGGCTATCTCGGCCTCCAA | GCCGGGCCTGAGGTATCCT |
| LinJ18_V3.1080 | Vacuolar protein sorting complex subunit, putative | AGATTGAGACGCGGATGGAAT | CGTTAGCAAATCGGGGTCGTT |
| LinJ19_V3.0710 | Glycosomal malate dehydrogenase | ACGGGTAAACCGCTGGTGTA | ACTCGCTGCCCTTCACGATA |
| LinJ19_V3.0090 | Fibrillarin, putative | ATCAAGGCGAACTGCATCGA | AGAAGCTGAAGGATTCCGGC |
| LinJ31_V3.1640 | Dipthine synthase, putative | CCTGGAGGTGGAGGGCTACA | GTAGCAGGGGCGATGGTGAA |
| LinJ31_V3.1660 | Putative 3-ketoacyl-CoA thiolase-like protein | GGCTGAAAAGGCGGGGTACT | CTTTTTGCCCGAACTCCGAA |
| LinJ32_V3.3690 | DEAD/DEAH box helicase, putative | AACGTGCGTGGACTGGATCT | ATGCCGCTTCTCCACACGAA |
| LinJ24_V3.0020 | Clathin coat assembly protein. putative | GGGCGTGGCAAGGCTAATTA | GGCGGATCTCTGCGAAGAAA |
| LinJ22_V3.1380 | Dephospho-CoA kinase. Putative | GCAGCGTATCGGTAGCCAGA | CAACACACGCGCACACAACT |
| LinJ14_V3. 1350 | Ubiquitin/ribosomal protein S27a. putative | ACGCAGGGCAACCTCTTCTT | TTCGTGAAGATGCGCTTCTT |
| LinJ28_V3.0090 | Adenylate cyclase-like protein | TACCTCTTCCGCAGCGTTGA | CAGGCCGACATCGCTCACTA |
| LinJ36_V3.0590 | Ubiquitin-like protein. putative | AGCAACCCCCAGTTTATGCA | AGCTTCCCATCATTGCCATT |
| LinJ28_V3.3060 | Heat-shock protein hsp70. putative | GAGGCGGCGAAGGAGGAGTA | CCGCCCATGCTCTGGTACAT |
| LinJ36_V3.3190 | Pre-mRNA branch-site protein p14 | GGGTCATCCTCCATCACGAA | GTGCGCCGACTGCATTATTT |
| LinJ18_V3.0830 | Periodic tryptophan protein 2-like protein | CTCCTGCTCCACTCCAACGA | ATGAAAGAGGCTGCGGTGAA |
| LinJ23_V3.1610 | Acetyltransferase-like protein | CACGGTGCCCTTCACCAAAA | CGGGCAGTGCTCCATGATGT |
| LinJ32_V3.0460 | 40S ribosomal subunit protein S2 | CGGCTACTGGGGCAACAAGA | ACGGCGACGGAACCACACTT |
| LinJ32_V3.0470 | Prostaglandin F synthase, putative | GAGCCGCACCACCTTGATGA | GCGCCTTCTGCTGGAAGTGA |
| LinJ15_V3.0170 | Protein phosphatase 2C | GAGGTAGCGCAGCAGCTTGT | GGCCTTTCGCTTTCTTGTCGT |
| LinJ15_V3.0180 | Serine/Threonine protein kinase, putative | CCGAAGCCCAGAGGTGATACT | CCCGAGAGTGGCTTCGATCT |
| LinJ24_V3.1510 | Multi drug resistance protein-like | CGGCGAGGCGGACCTTTATA | CGGCGACCTCACATTCTTGA |
| LinJ24_V3.1380 | Translation initiation factor IF2, putative | CGACTCGCTTGAATGCCAACT | GCGAGGTACGCACCAATGGA |
| LinJ21_V3.0800 | 60S ribosomal protein L36 | CGCGCTGTGAAGGCTATCAT | CTTGCCGACACGCAGAAACT |
| LinJ32_V3.3110 | Nucleoside diphosphatase kinase b | GACTCACAGCCTGGCACGAT | CTCATCCGCCTTGAACCAAAA |
| LinJ32_V3.3120 | DNA replication licensing factor | GGAGCGCGATGCTGAGCTTT | TGGGTCAACGTAGGGGTGAA |
| LinJ32_V3.3130 | ATP-binding protein-like protein | CTAGAGGTTGCGGAGCCAAA | AGTAACTTGCCGTAGACGCG |
| LinJ30_V3.0550 | Glycosyltransferase family 28 protein, putative | CCGTGTGGTCGAAGCGTTGT | CACCGTGTGGTCGAAGCGTT |
| LinJ30_V3.0560 | Nuclear cap binding protein | CACGGACTCGACGGGTCACT | GCAGCCCACATCCCAGCTAA |
| LinJ30_V3.0570 | Hypothetical protein, conserved | ATGCCGCTGTGCTTCAACTT | GGCGATGAAGGGAATCATGA |
| LinJ35_V3.2030 | Ankyrin repeat protein, putative | GCCCCGTCCTCATCGAGGTA | CCTCGCCCATCAGCAGCAGT |
| LinJ35_V3.2040 | 60S ribosomal subunit protein L32 | CCGATCCAAAACGTGCAGGA | ACGATGGCCTTGCGAGACCT |
| LinJ19_V3.0170 | Mitogen activated protein kinase | CCTGCAGCACCCGTATTTCA | GCCCACATTCGACATCGACT |
| LinJ30_V3.0790 | Hypothetical protein, conserved | CCGAGCCACCAACAAGCGTCA | TGATGCCCCAACTGTCCGAAT |
| LinJ30_V3.0800 | 4-methyl-5-(betahydroxyethyl)thiazole monophosphate synthesis protein | ATGCCAGGAGCCGTCCATCT | CAGCAGCCCCATAGGAGCAA |
| LinJ13_V3.1450 | Alpha tubulin, putative | ACGTACCGCCAGCTGTTCAA | CTGGAGACCCGTGCAGTTGT |
| LinJ27_V3.0950 | Hypothetical protein-similar to OGlcNac transferase | GATCGGGCTGCTCGAAGGAT | GATCCACGAGGGCGACGCTA |
| LinJ34_V3.1150 | Amastin-like surface protein | GCGTCGGCTGTCATCTCCAT | GCCCAAGGTGAAGATGCCAA |
| LinJ32_V3.1400 | Cleavage and polyadenylation specificity factor-like protein | CAGGAGGAGCTGAACGCCAT | GCACCGCAAAGAGAATGGAA |
| LinJ14_V3.1500 | Phosphoglycan beta-1,3-galactosyltransferase | TCCGCGCCTCTTCTTCCTTT | CCTTCACGGCCTCCGCATA |
| LinJ31_V3.1980 | Transcription-like protein nupm1, putative | TCGAGCGCAGCAATCTGGAA | TCCACCACGGACGGCATATT |
| LinJ23_V3.0230 /40 | ABC transporter, putative | GGGAGCGGGTTCATCCTGAT | TGCAGCCGGTGCGCGATCGT |
| LinJ25_V3.2570 | Phosphoglycan beta-1,3-galactosyltransferase 4 | TAGACCGTCGCCTCATCCAA | TGCATGAGCAAGCTCGCATA |
| LinJ34_V3.2190 | Glycosyltransferase-like protein | TGGCGTACTCGAAGCCGGTT | GCCGCACTTTGACGGGTCTT |
| LinJ34_V3.2200 | DnaJ-like protein | AACACCTCGGCGACCACTTT | GCGAGAGACACCTAATTGAA |
| LinJ29_V3.2420 | Enoyl-CoA hydratase/isomerase-like protein | GTGGGTTCTCTTGGGCAACA | CGGCATCAGCTCCTCGTGTT |
| LinJ24_V3.0910 | DNA polymerase theta, polymerase domain, putative | AAGTCCCTCATCGCCGAGAA | AGCAGGGAGGTAAGGCAGAAA |
